# Effect of postoperative systemic prednisolone on short-term and long-term outcomes in chronic rhinosinusitis with nasal polyps: A multi-centered randomized clinical trial

**DOI:** 10.3389/fimmu.2023.1075066

**Published:** 2023-03-08

**Authors:** Sarina K. Mueller, Olaf Wendler, Susanne Mayr, Maximilian Traxdorf, Werner Hosemann, Heidi Olze, Helmut Steinhart, Susanne Wiegand, Afshin Teymoortash, Thomas Kuehnel, Stephan Hackenberg, Thomas Hummel, Petra Ambrosch, Azita Fazel, Bernhard Schick, Hanns-Wolf Baenkler, Michael Koch, Harald Buerner, Konstantinos Mantsopoulos, Philipp Grundtner, Angela Nocera, Abbas Agaimy, Benjamin Bleier, Heinrich Iro

**Affiliations:** ^1^ Department of Otolaryngology, Head and Neck Surgery, Friedrich-Alexander-Universität Erlangen-Nürnberg, Erlangen, Germany; ^2^ Department of Otolaryngology, Head and Neck Surgery, Helios Hanseklinikum Stralsund, Stralsund, Germany; ^3^ Department of Otolaryngology, Head and Neck Surgery, Universitätsklinikum Berlin, Berlin, Germany; ^4^ Department of Otolaryngology, Head and Neck Surgery, Marienhospital Stuttgart, Stuttgart, Germany; ^5^ Department of Otolaryngology, Head and Neck Surgery, Phillips Universität Marburg, Marburg, Germany; ^6^ Department of Otolaryngology, Head and Neck Surgery, Universitätsklinikum Leipzig, Leipzig, Germany; ^7^ Department of Otolaryngology, Head and Neck Surgery, Universitätsklinikum Regensburg, Regensburg, Germany; ^8^ Department of Otorhinolaryngology, Head and Neck Surgery, Universitätsklinikum Aachen, Aachen, Germany; ^9^ Department of Otolaryngology, Head and Neck Surgery, Smell and Taste Clinic, Universitätsklinikum Carl Gustav Carus Dresden, Dresden, Germany; ^10^ Department of Otolaryngology, Head and Neck Surgery, Christian-Albrechts-Universität (CAU) Kiel, Kiel, Germany; ^11^ Department of Otolaryngology, Head and Neck Surgery, Universitätsklinikum des Saarlandes, Homburg, Germany; ^12^ Department of Rheumatology and Immunology, Friedrich-Alexander-Universität Erlangen-Nürnberg, Erlangen, Germany; ^13^ Department of Otolaryngology Harvard Medical School, Massachusetts Eye and Ear Infirmary, Boston, MA, United States; ^14^ Department of Pathology, Friedrich-Alexander-Universität Erlangen-Nürnberg, Erlangen, Germany

**Keywords:** nasal polyp (NP), prednisolone, chronic rhinosinusitis, postoperative, long-term, randomized controlled trial

## Abstract

**Introduction:**

The objective of this study was to determine whether postoperative additive systemic steroid administration in chronic rhinosinusitis with nasal polyps (CRSwNP) impacted selected endoscopic, subjective and objective outcome measures.

**Methods:**

This was a prospective, randomized, double-blind, placebo-controlled, noninferiority multicenter trial of n=106 patients with CRSwNP. All patients underwent primary functional endoscopic sinus surgery (FESS) followed by topical nasal steroids. Patients were randomized to a systemic steroid or placebo for 1 month. Patients were followed up for 2 years over 9 time points. The primary outcome measures were the differences between groups with respect to the nasal polyp score (NPS) and sinonasal quality of life (SNQoL). Secondary outcome measures included interactions with respect to the Lund-Kennedy score (LKS), sinonasal symptoms, general quality of life (GQoL), 16-item odor identification test scores, recurrence rates, need for revision surgery and mucus biomarker levels.

**Results:**

106 patients were randomized to either the placebo or the systemic steroid group (n=53 per group). Postoperative systemic steroids were not superior to placebo with respect to all primary (p= 0.077) and secondary outcome measures (p>0.05 for all). Reported adverse events were similar between the two groups.

**Conclusion:**

In conclusion, the addition of postoperative systemic steroids after primary FESS did not confer a benefit over topical steroid nasal spray alone with respect to NPS, SNQOL, LKS, GQOL, sinonasal symptoms, smell scores, recurrence rates, the need for revision surgery or biomarkers over a short-term follow-up of up to 9 months and a long-term follow-up of up to 24 months in CRSwNP patients. Functional endoscopic surgery did, however, show a strong effect on all outcome measures, which remained relatively stable up to the endpoint at 2 years.

## 1 Background

Chronic rhinosinusitis (CRS) is a common disorder, with an estimated 15% of the general population being affected based on symptomatology (1). The socioeconomical burden of the disease in Europe is estimated to be €1501 per patient/year of total direct costs (outpatient department visits, hospitalization) and €5659 per patient/year of indirect costs (missed workdays, decreased productivity), more than than 20 billion US$ for joint direct and indirect costs in the US and £2.8 billion per million inhabitants in the UK (2–4). CRS with nasal polyps (CRSwNP) is known to have a significant impact on quality of life, greater in some respects than in other chronic diseases such as lower back pain or chronic obstructive pulmonary disease (COPD) (5–7). Amongst nasal saline rinses, nasal corticosteroids remain the first-line conservative treatment option for CRSwNP. Previous studies have shown a positive impact on specific and general quality of life (SNQOL, GQOL) and the nasal polyp score (NPS) in patients with CRS (1, 8, 9). The effect of a course of systemic corticosteroids has been proven for CRSwNP patients in the preoperative phase, in combination with or without local corticosteroid treatment, and results in a significant reduction in the total symptom and nasal polyp score (1). Literature also shows that the combination of systemic and topical steroids may be superior to topical steroids alone (10). For medically refractory patients, endoscopic sinus surgery remains the gold standard treatment. The majority of patients with nasal polyps also continue medical therapy including topical steroids after surgery. However, it is still unclear whether there is an added benefit from systemic steroids in the perioperative period. Four previous randomized controlled trials (RCT) (11–14) have already been published on this topic. However, only a couple of parameters were investigated and follow-up data did not exceed 6 months. Consequently, up to now there is no strong evidence for or against additive systemic steroids in the postoperative period. And although the EPOS 2020 (1) summarizes that no RCT showed a benefit of postoperative additive steroids, they are still commonly used in the postoperative setting due to the lack of detailed data. Furthermore, no previous RCT has examined a multitude of parameters including recurrence rates, the need for revision surgery, olfactory changes using the 16-item smell identification test or mucus biomarker levels. To address these shortcomings, our group conducted a multicenter, randomized, placebo-controlled clinical trial with one of the largest sample sizes to date. The objective of this RCT was to determine whether postoperative systemic steroid administration in CRSwNP impacted selected short-term (surgery to 9 months) and long-term (12-24 months) primary and secondary outcome measures.

## 2 Methods

This was a prospective, randomized, double-blind, placebo-controlled multicenter trial of n=106 CRSwNP patients that was approved by the institutional review boards of the Friedrich-Alexander University Erlangen‐Nürnberg (FAU), Philipps University Marburg, Christian-Albrecht University Kiel (CAU), Charité Berlin, Marienhospital Stuttgart and the University of Regensburg. Patients were recruited between 2005 and 2012 (IRB No. 3201). Molecular biological experiments for determining potential non-invasive biomarkers and analyzing the clinical results were conducted between 2013 and 2020 (IRB Nos. No: 4_20; No: 269_17). All patients consented to the study.

### 2.1 Inclusion criteria

CRSwNP was defined using the ICAR : RS criteria (15). Patient demographics were assessed. All included CRSwNP patients were between 18 and 80 years of age and did not have a history of previous sinus surgery. Prior to surgery, all patients had been treated with saline rinses and a topical steroid spray (200 micrograms of mometasone furoate) twice daily for at least three months and with systemic steroids as well as antibiotics as needed. If the patients were recalcitrant to the topical steroid and nasal rinses, a computed tomography (CT) was performed. For all patients showing a Lund-Kennedy score ≥ 1 (LKS) and a Lund-Mackay score ≥ 10 (LMS), functional endoscopic sinus surgery (FESS) was recommended. Before surgery, all patients underwent a 4-week washout period for all systemic steroids, topical steroids and antibiotics.

### 2.2 Exclusion criteria

Exclusion criteria included primary ciliary dysfunction, autoimmune disease, cystic fibrosis, or immunodeficiency. Additionally, patients <18 and >80 were excluded, as were pregnant patients and patients with significant cognitive impairment or a poorly controlled psychiatric disorder.

### 2.3 Outcome measures

#### 2.3.1 Primary outcome measures

##### 2.3.1.1 Nasal polyp score and sinonasal quality of life

The primary outcome measures included the nasal polyp score as an endoscopic measure and the rhinosinusitis disability index (RSDI) as a patient-reported outcome measure (PROM). Differences between the two groups were assessedat all time points. The endoscopic nasal polyp score was assessed at each time point with a 30° rigid endoscope (Karl Storz, Tuttlingen, Germany) for each nostril separately. The highest score per side was graded. Each nostril was scored from 0-4 (0=no polyps, 1= small polyps confined to the middle meatus, 2= blocked middle meatus 3= polyps extending beyond middle meatus, 4=large polyps causing almost complete nasal obstruction). Simultaneously, endoscopic pictures were taken for documentation purposes. At all time points, the endoscopy was recorded in a standardized fashion and was interpreted by a central investigator.

The RSDI was used for the assessment of sinonasal-specific quality of life. The RSDI is a validated 30-item Likert-type scale instrument containing three subscales assessing the impact of sinusitis on physical, functional, and emotional domains (range: 0–120) (16). Higher total and subscale RSDI scores represent a worse impact of sinus disease (0=no impact, 120=highest impact). The questionnaires were given to the patients at the beginning of each visit and were filled out by the patients.

### 2.4 Secondary outcome measures

Secondary outcome measures included a change in Lund-Kennedy score, recurrence rates, need for revision surgery (<2 years), sinonasal symptoms, general quality of life, biomarker levels, and smell scores.

### 2.5 Endoscopy

Endoscopic Lund-Kennedy scores were also assessed with a 30° endoscope for each nostril separately. Lund-Kennedy scores range from 0 to 20. Scoring includes the assessment of polyps (0=no polyps, 1=middle meatus, 3=beyond middle meatus), discharge (0=no discharge, 1= thin discharge, 2=thick or purulent discharge) and edema/scarring/crusting (0=absent, 1=mild, 2=severe). The highest score per side was graded. At all time points, the endoscopy was recorded in a standardized fashion and was interpreted by a central investigator.

### 2.6 Recurrence rates and need for revision surgery

Recurrence rates were defined in two different ways: 1) as an increase in NPS ≥ 2 in ≥ 2 consecutive time points within the 2-year follow-up. This definition was chosen in order to distinguish between edematous mucosa in the postoperative phase and a recurrence of nasal polyps; 2) as an increase in RSDI (16, 17) from the preoperative value. All patients who needed revision surgery within the two years of follow-up were assessed and counted as revision surgery.

### 2.7 Sinonasal symptoms

Sinonasal symptoms including nasal congestion, anterior rhinorrhea, posterior rhinorrhea, sneezing, tearing, facial pain or pressure, headache were assessed and graded from 0-3 (0=no problems, 1=mild problems, 2=moderate problems, 3=severe problems). The sense of smell was graded from 0-2 (0=normal, 1=mild problems, 2=not possible).

### 2.8 Patient-reported quality of life: General quality of life

For patient-reported outcomes, standard general quality of life questionnaires were used. The questionnaires were given to the patients at the beginning of each visit and were filled out by the patients.

For the assessment of the general quality of life, the SF-36 was used (18, 19). It is one of the most widely used generic measures of health-related quality of life. There is no single overall score for the SF-36; instead it generates 8 subscales and two summary scores. The 8 subscales are physical functioning, role limitations due to physical problems, bodily pain, general health perceptions, vitality, social functioning, role limitations due to emotional problems, and mental health. The scoring ranges from 0-100, whereas higher total and subscale SF-36 scores represent a better health situation (0=highest health limitation, 100=no health limitation).

### 2.9 Olfaction testing

For olfaction testing, a 16-item smell identification test using the Sniffin´ Sticks was used (20). In the Sniffin´ Sticks screening, test patients received 16 odors presented consecutively. Patients were asked to identify the odors from flashcards with four verbal descriptors each (“forced choice”). The test was graded from 0-16, with a higher score indicating a better sense of smell (anosmia was defined as a score ≤7) (21).

### 2.10 Mucus biomarkers

As mucus-derived biomarkers, CST1, CST2, PAPP-A, Periostin, SerpinE1, SerpinF2, MMP9 and IgE were assessed. Those biomarkers had been identified and validated in previous studies of our group (22–28). This combination of biomarkers was chosen due to previous validation by other groups MMP9, IgE (29, 30) or our own group (CST1, CST2, PAPP-A, Periostin, SerpinE1, SerpinF2) (26, 27, 31, 32).

### 2.11 Biomarker analysis

#### 2.11.1 Mucus collection technique

Mucus samples were taken at each time point. At time point -2, samples were taken before the washout period. At time point 0, samples were taken after the 4-week washout period. At all other time points, samples were taken prior to antibiotic or steroid administration. Samples were taken by applying a polyvinyl alcohol sponge (PVA, Medtronic, Minneapolis, MN) to the anterior internal valve, taking care not to abrade the mucosa or contaminate the sponge with blood. The sponges were weighed prior to and after insertion into the nose. Then, the sponge was soaked with 3 ml of PBS. After 10 minutes, the sponges were centrifuged for 10 minutes at 2000 xg. The eluate was frozen immediately into aliquots at -80°C and was stored at -80°C until analysis.

#### 2.11.2 protein biomarker analysis using enzyme-linked immunosorbent assay

Eight proteins including CST1, CST2, PAPP-A, Periostin, SerpinE1, SerpinF2, MMP9 and IgE were analyzed from mucus at all 10 time points based on our prior studies (24–28). After thawing of the aliquots, CST2, PAPP-A, Periostin, SerpinE1, SerpinF2, MMP9 and IgE were quantified in the nasal mucus using enzyme-linked immunosorbent assay (ELISA) and normalized to total protein (BCA-Assay, Life Technologies, Darmstadt, Germany). All ELISAs were performed according to manufacturer protocols and are displayed in detail in the appendix.

### 2.12 Baseline testing

Baseline values were defined as the parameters assessed at the first visit. These parameters were assessed after the 4-week washout period. LMS were only assessed at the first visit. The LMS assesses the opacity of the paranasal sinuses and the osteomeatal complex, grading each side from 0-12 (0=no abnormality, 1=partial opacification, 2=complete opacification, total score 0-24).

### 2.13 Allergy and aspirin intolerance testing

All patients underwent a 4-week washout period for all systemic steroids, topical steroids and antibiotics. Diagnostic criteria for asthma, AERD (aspirin-associated respiratory disease) and allergic rhinitis were based on clinical history, skin prick and the peripheral blood aspirin intolerance test (33). The peripheral blood aspirin intolerance test measures the relation between prostaglandins (PGE2) and leukotrienes (functional eicosanoid test=FET). The FET was graded as follows: FET-0 = normal; FET-1= modified; FET-2 = abnormal; FET-3 = severe abnormal eicosanoid pattern.

### 2.14 Surgery

Functional endoscopic sinus surgery was performed following the same surgical technique (protocol of Wigand (complete functional endoscopic sinus surgery with opening of the maxillary sinus, the anterior/posterior ethmoid sinus, the sphenoid sinus and the frontal sinus (Draf IIa) (34)). A septoplasty and turbinate reduction was additionally performed if needed.

### 2.15 Postoperative medication regimes and randomization

After FESS, the packing was removed on the second postoperative day. Patients were treated with topical corticosteroid nasal spray (200 micrograms mometasone furoate twice daily) for 3 months after removal of the packing. Patients were randomized to either a systemic steroid (n=53) or a placebo (n=53) from postoperative day 1. The steroid group received a steroid taper starting from 80 mg prednisolone. The dose was tapered down to 5 mg by day 14 and the 5-mg dose was continued for 14 additional days. The placebo group received lactose monohydrate tablets. The systemic steroid and the placebo were identical in appearance and were dispensed by staff members who were blinded to the content and the randomization. Both groups received tablets of 25 mg and 5 mg. Randomization was performed according to a 1:1 randomization pattern calculated by our statistician. A patient diary and a bi-weekly telephone consultation asking about current medication and symptoms were used to ensure patient compliance. Rescue medication (oral/topical steroids as well as antibiotics) was allowed if medically indicated.

### 2.16 Patient follow-up

All patients were followed up over a 2-year time period at 9 different time points. Patients were recruited and then had a 4-week washout period for all systemic and topical steroids as well as antibiotics. The first time point was preoperatively on the day of surgery. The remaining 8 postoperative time points were at 3 weeks, 1.5, 3, 6, 9, 12, 18, and 24 months after FESS.

### 2.17 Side effect and current medication evaluation

At each visit, therapy-related side effects were evaluated (e.g. heart rate, oxygen level, measurement of blood pressure, blood sugar level, signs of infection during endoscopy). Safety assessments included adverse and severe adverse events (AEs, SAEs) as defined in the study protocol.

### 2.18 Additional effect of surgery

The effect of surgery was evaluated between the timepoints 0 (preoperative) and 0.75 (first postoperative time point measured). Differences in all primary and secondary outcome measures were evaluated.

### 2.19 Subgroup analysis moderate/severe eosinophilia group

Tissue eosinophil counts were assessed by the Department of Pathology, University of Erlangen-Nürnberg on the polyp tissue collected during surgery (0=no eosinophils, 1=mild eosinophilia, 2=moderate eosinophilia, 3=severe eosinophilia). In detail, 0 equals no eosinophils, 1 equals scattered eosinophils distributed in the tissue, 2 equals smaller accumulations of eosinophils at individual sites in the tissue and 3 equals large clusters of eosinophils covering the entire tissue. In order to determine whether tissue eosinophilia levels have an impact on primary and secondary outcome measures, we performed a subgroup analysis comparing all patients graded into the moderate and severe eosinophilia group with the rest of the included patients.

### 2.20 Statistical analysis

To test whether the time-related changes within primary and secondary outcome measures were dependent on patient group, a linear mixed model was conducted for each parameter. “Time” (0, 0.75, 1.5, 3, 6, 9, 12, 18 and 24 months) and “group” (steroid vs. placebo) were defined as fixed factors, with the variable of interest being labeled as repeated measurement. “Patient ID” was chosen as the random effect (modeling random intercepts).

By calculating a linear mixed model (LMM), missing values of a patient did not lead to the patient’s exclusion from data analyses. The interaction of “group x time” was defined as the target effect. In case of a significant interaction, pairwise comparisons were conducted to test for significant differences between groups at each point in time (resulting in 9 post-hoc independent t-tests). All pairwise comparisons were Holm corrected to avoid alpha inflation (correction for multiple comparisons).

Continuous variables were tested for normal distribution *via* their histogram and the Kolmogorov Smirnov test. In case of normal distribution, variables were presented as mean and standard deviation and group differences were tested with independent t-tests. In case of non-normally distributed data, variables were presented as median and interquartile range and differences between groups were tested using the Mann-Whitney U-Tests.

Effect sizes are provided in terms of R^2^ for the interaction, by Cohen’s d at t-test level and by Phi at Chi^2^-test level (for reference: d = 0.2 = small effect; 0.5 = moderate effect, 0.8 = strong effect; and R^2^ as an indicator of the amount of variance explained by the interaction effect, Phi=0.1 = small effect; Phi = 0.3 = moderate effect; Phi = 0.5 = strong effect).

Categorical data are displayed as absolute and relative frequencies (N/%) and compared *via* Chi^2^-Tests/Fishers Z. Data were analyzed with R v. 4.0.3 and SPSS (SPSS Statistics 26, Armonk, NY: IBM Corp.).

The effect of surgery on the primary and secondary outcome measures was calculated as the change between timepoint 0 and 0.75.

#### 2.20.1 Sample size estimation

The sample size was estimated for the primary outcome measure of this study (NPS), modeling the interaction term “group x time” with alpha = 0.05 and 1-ß = 0.8. It was based on Harissa et al. (2006) who investigated changes in the nasal specific subscores of the RSOM in patients with sinonasal polyposis, who received either oral prednisolone (50 mg, N = 20) or a placebo (N = 20) over a period of 14 days, with no other treatment being given. In this double-blind RCT, strong decreases in symptoms from pre to post were reported for the intervention group (64%, p < 0.001, with d = 1.4), while there was no significant decrease in the control group (11%, p > 0.05, with d = 0.35). While no significant difference between groups was found before treatment started (p > 0.05, with r = 0.04), a substantial difference was found after 14 days (p < 0.001, with d = 1.71). With no interaction term being reported, we concluded, based on the reported means/SDs and t-Tests, that the effect size of the observed interaction (time x group) would have been at least medium-sized. Therefore, to detect at least medium-sized effects, 12 participants per group would have been required.

Based on the assessed study population of 53 participants per group, we were able to detect effect sizes of at least $\text{Et}{\text{a}}_{p}^{2}=0.019$ , equaling a small effect.

Sample size calculation was conducted in G*Power (3.1.9.4).

## 3 Results

**Figure 1:**
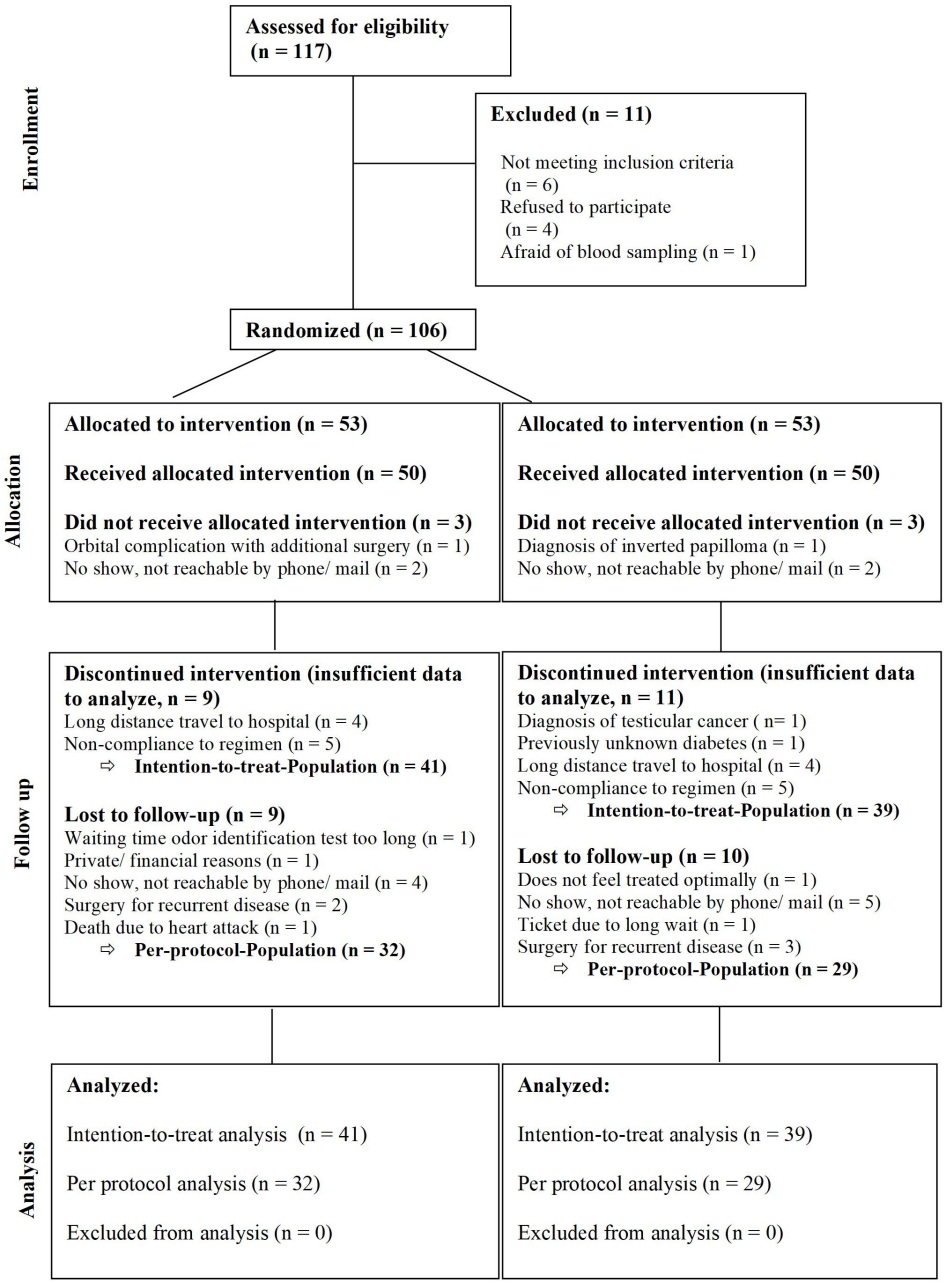
CONSORT diagram of the study.

A total of 106 patients were randomized to receive prednisolone as systemic steroid (n=53, steroid=S) or a placebo (n=53, P). 80 patients completed the medication regimen. This included 41 patients in the steroid and 39 patients in the placebo group (intention-to-treat group). 61 patients completed the 24 months follow-up (per protocol group) (**Figure 1**). All results displayed here will be the results of the intention-to-treat group. Patient demographic and baseline characteristics were well balanced between the steroid and the placebo group with no significant differences (**Tables 1**, **2**). Additionally, an analysis comparing the baseline characteristics and demographics for the intention-to-treat (ITT) and the dropout group was performed for the steroid and for the placebo group separately (**Tables 3A–D**). For the steroid group, gender (more males in ITT), asthma (more asthmatics in the ITT), and nasal congestion (worse scores in the dropout group) were significantly different. In the placebo group, the symptoms nasal congestion (worse scores in the dropout group) and headache (worse scores in the ITT group) were statistically different. For all other parameters, there was no statistical significance. The effect sizes were mostly small or moderate.

**Table 1:** Patient demographics of the intention-to-treat group (n=80) showing no significant differences between the steroid and the placebo group.

Characteristics n (%)	Steroid (n = 41)	Placebo (n = 39)	p-value
**Mean age in years (± SD)**	48.54 ± 10.91	50.90 ± 13.44	0.39
**Gender**			
Male	35/41 (85.4)	29/39 (85.7)	0.22
Female	6/41 (14.6)	10/39 (14.3)	0.22
**Race**			
Caucasian	41/41 (100)	39/39 (100)	1.0
**Comorbidity**			
Asthma	15/41 (36.6)	11/39 (28.2)	0.42
Environmental allergy	19/41 (46.3)	19/39 (48.7)	0.83
AERD	5/41 (12.1)	10/39 (25)	0.12
**Eosinophilia in histology (score ± SD)**	1.93 ± 0.95	1.93 ± 0.94	0.99
**Sinus symptoms per year (score ± SD)**	1.18 ± 1.12	1.18 ± 0.98	0.99
**Smoking (score ± SD)**	0.82 ± 0.86	0.67 ± 0.78	0.46

**Table 2:** Baseline testing for all primary and secondary outcome measures as well as for the Lund-Mackay score (intention-to-treat group, n=80).

Outcome measure	Steroid (n = 41)	Placebo (n = 39)	p-value
Primary			
**Nasal Polyp Score**	4.59 ± 2.45	5.33 ± 2.29	0.16
**SNQoL-RSDI**	68.0 ± 23.41	63.0 ± 23.78	0.33
RSDI Functional	22.0 ± 7.64	21.0 ± 7.67	0.47
RSDI Emotional	19.0 ± 8.42	17.0 ± 7.95	0.43
RSDI Physical	24.0 ± 8.29	21.0 ± 8.39	0.21
Secondary			
Sinonasal symptoms			
Nasal Congestion	1.73 ± 0.78	1.97 ± 0.82	0.18
Anterior rhinorrhea	1.66 ± 0.91	1.79 ± 0.81	0.50
Posterior rhinorrhea	1.46 ± 0.98	1.37 ± 0.91	0.66
Sense of smell	1.37 ± 0.79	1.29 ± 0.91	0.72
Sneezing	1.51 ± 0.78	1.41 ± 0.79	0.55
Headaches	0.88 ± 0.87	1.29 ± 1.01	0.06
GQoL SF-36			
SF-36 Physical Functioning	84.76 ± 15.65	83.47 ± 18.63	0.94
SF-36 Role Physical	71.95 ± 36.74	78.29 ± 30.85	0.41
SF-36 Pain Index	68.98 ± 23.31	73.50 ± 22.05	0.38
SF-36 General Health Perception	59.73 ± 18.97	61.24 ± 18.82	0.72
SF-36 Vitality	57.44 ±22.48	64.21 ± 20.91	0.17
SF-36 Social Functioning	80.18 ± 19.05	84.54 ± 19.70	0.33
SF-36 Role Emotional	74.79 ± 39.29	83.33 ± 32.65	0.29
SF-36 Mental Health index	75.71 ± 16.19	72.22 ± 16.94	0.36
Biomarker			
CST-2	310.59 ± 347.73	338.32 ± 448.43	0.8
PAPP-A	110.97 ± 92.78	109.08 ± 76.28	0.9
SerpinE1	214.0 ± 202.62	142.88 ± 125.12	0.12
Periostin	181.97 ± 322.80	156.22 ± 175.66	0.75
SerpinF2	2846.11 ± 1765.30	2544.08 ± 1601.45	0.49
CST-1	15.90 ± 20.91	11.30 ± 12.38	0.33
MMP-9	138.30 ± 107.25	84.40 ± 126.98	0.09
IgE	13.46 ± 19.29	10.52 ± 16.36	0.54
**16-item odor identification**	6.0 ± 2.0	5.86 ± 3.22	0.98
**Lund-Kennedy-Score**	6.93 ± 2.79	7.22 ± 3.02	0.66
**Lund-Mackay Score**	14.94 ± 4.75	14 ± 4.36	0.41

There were no significant differences between the steroid and the placebo group, SNQOL, sinonasal quality of life; GQOL, general quality of life; RSDI, rhinosinusitis.

**Table 3:** Table comparing the baseline demographics and baseline characteristics for the intention-to-treat (n=80) and the dropout (n=26) groups (A) demographics for the steroid group (B) demographics for the placebo group (C) further characteristics for the steroid group (D) further characteristics for the placebo group.

Characteristics n (%)	intention-to-treat (ITT) n=41	dropouts (DO) n=14	p-value ITT vs. DO	effect size phi
**Gender**				
Male	35/41 (85.4)	7/14 (50)	0.007	0.363
Female	6/41 (14.6)	7/14 (50)	0.007	0.363
**Race**				
Caucasian	41/41 (100)	14/14 (100)	1	
**Comorbidity**				
Asthma	15/41 (36.6)	1/14 (7.1)	0.036	0.282
Environmental allergy	19/41 (46.3)	3/14 (21.4)	0.100	0.222
AERD	5/41 (12.1)	2/14 (14.3)	0.839	0.027
B) Placebo				
Characteristics n (%)	intention-to-treat (ITT) n=39	dropouts (DO) n=12	p-value ITT vs. DO	effect size phi
Gender				
Male	29/39 (85.7)	9/12 (75)	0.964	0.006
Female	10/39 (14.3)	3/12 (25)	0.964	0.006
**Race**				
Caucasian	39/39 (100)	12/12 (100)	1	
**Comorbidity**				
Asthma	11/39 (28.2)	5/12 (41.6)	0.379	0.123
Environmental allergy	19/39 (48.7)	4/12 (33.3)	0.349	0.131
AERD	10/39 (25)	2/12 (16.7)	0.522	0.090
C) Steroid				
Characteristics n (%)	intention-to-treat (ITT) n=41	dropouts (DO) n=14	p-value ITT vs. DO	effect size d
Mean age in years (± SD)	48.54 ± 10.91	44.29 ± 10.49	0.989	0.004
Eosinophilia in histology (score ± SD)	1.93 ± 0.95	1.57 ± 1.30	0.305	0.391
NPS	4.59 ± 2.45	4 ± 2.87	0.856	0.050
LKS	6.93 ± 2.79	7 ± 3.82	0.930	0.024
16-item odor identification test	6.0 ± 2.0	8 ± 3.67	0.394	0.283
RSDI	68.0 ± 23.41	59 ± 23.82	0.916	0.030
RSDI Functional	22.0 ± 7.64	19 ± 7.06	0.809	0.069
RSDI Emotional	19.0 ± 8.42	16 ± 8.22	0.857	0.051
RSDI Physical	24.0 ± 8.29	21 ± 9.31	0.983	0.006
SF-36: Physical Functioning	84.76 ± 15.65	78 ± 25.44	0.586	0.155
SF-36: Role-Physical	71.95 ± 36.74	77 ± 31.39	0.891	0.039
SF-36: Pain Index	68.98 ± 23.31	72 ±24.75	0.346	0.272
SF-36: General Health Perceptions	59.73 ± 18.97	64 ± 19.23	0.892	0.038
SF-36: Vitality	57.44 ±22.48	58 ± 19.10	0.386	0.249
SF-36: Social Functioning	80.18 ± 19.05	82 ± 25.83	0.433	0.225
SF-36: Role Emotional	74.79 ± 39.29	97 ± 9.24	0.075	0.540
SF-36: Mental health index	75.71 ± 16.19	68 ± 16.61	0.334	0.279
nasal congestion	1.73 ± 0.78	2 ± 0.67	0.032	0.711
anterior rhinorrhea	1.66 ± 0.91	2 ± 0.90	0.324	0.298
posterior rhinorrhea	1.46 ± 0.98	1 ± 1.12	0.839	0.063
sneezing	1.51 ± 0.78	1 ± 1.14	0.293	0.319
headache	0.88 ± 0.87	1 ± 0.99	0.862	0.051
sense of smell	1.37 ± 0.79	1 ± 0.79	0.069	0.582
D) Placebo				
Characteristics n (%)	intention-to-treat (ITT) n=39	dropouts (DO) n=12	p-value ITT vs. DO	effect size d
Mean age in years (± SD)	50.90 ± 13.44	43.27 ± 13.40	0.109	0.504
Eosinophilia in histology (score ± SD)	1.93 ± 0.94	1.71 ± 1.38	0.689	0.159
NPS	5.33 ± 2.29	5 ± 3.18	0.645	0.137
LKS	7.22 ± 3.02	7 ± 4.2	0.061	0.603
16-item odor identification test	5.86 ± 3.22	6 ± 3.25	0.830	0.102
RSDI	63.0 ± 23.78	77 ± 23.92	0.388	0.287
RSDI Functional	21.0 ± 7.67	27 ± 7.34	0.098	0.585
RSDI Emotional	17.0 ± 7.95	21 ± 9.73	0.542	0.200
RSDI Physical	21.0 ± 8.39	25 ± 7.98	0.639	0.153
SF-36: Physical Functioning	83.47 ± 18.63	67 ± 27.33	0.447	0.239
SF-36: Role-Physical	78.29 ± 30.85	54 ± 43.74	0.912	0.034
SF-36: Pain Index	73.50 ± 22.05	62 ± 23.88	0.934	0.026
SF-36: General Health Perceptions	61.24 ± 18.82	53± 22.33	0.795	0.080
SF-36: Vitality	64.21 ± 20.91	57 ± 24.53	0.960	0.016
SF-36: Social Functioning	84.54 ± 19.70	77 ± 21.87	0.764	0.093
SF-36: Role Emotional	83.33 ± 32.65	56 ± 43,42	0.669	0.133
SF-36: Mental health index	72.22 ± 16.94	75 ± 20.01	0.810	0.078
nasal congestion	1.97 ± 0.82	2 ± 0.63	0.021	0.952
anterior rhinorrhea	1.79 ± 0.81	2 ± 0.82	0.312	0.360
posterior rhinorrhea	1.37 ± 0.91	1 ± 1.14	0.738	0.116
sneezing	1.41 ± 0.79	1 ± 1.13	0.217	0.480
headache	1.29 ± 1.01	1 ± 0.84	0.032	0.867
sense of smell	1.29 ± 0.91	2 ± 0.70	0.081	0.667

### 3.1 Primary outcome measures

**Figure 2:**
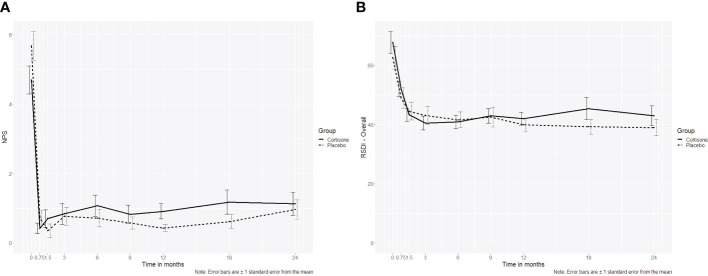
Line plot of **(A)** the nasal polyp score (NPS) over the 2-year follow-up showing no interaction between the steroid and the placebo group (p=0.507), **(B)** Rhinosinusitis Disability Index (RSDI, p= 0.433) over the 2-year follow-up showing no interaction between the steroid and the placebo group; the dotted line represents the placebo group, the continuous line represents the steroid group.

For the NPS a significant interaction “group x time” (p = 0.026) was found. Still at post-hoc level the groups did not differ significantly at any time point at either the short-term or the long-term follow-up (p ≥ 0.055 if uncorrected/p ≥ 0.498 if corrected for multiple comparison). The RSDI as a sinonasal-specific patient-reported outcome measure did not differ between the two groups over time either (p=0.462) (**Figures 2A, B**).

### 3.2 Secondary outcome measures

**Figure 3:**
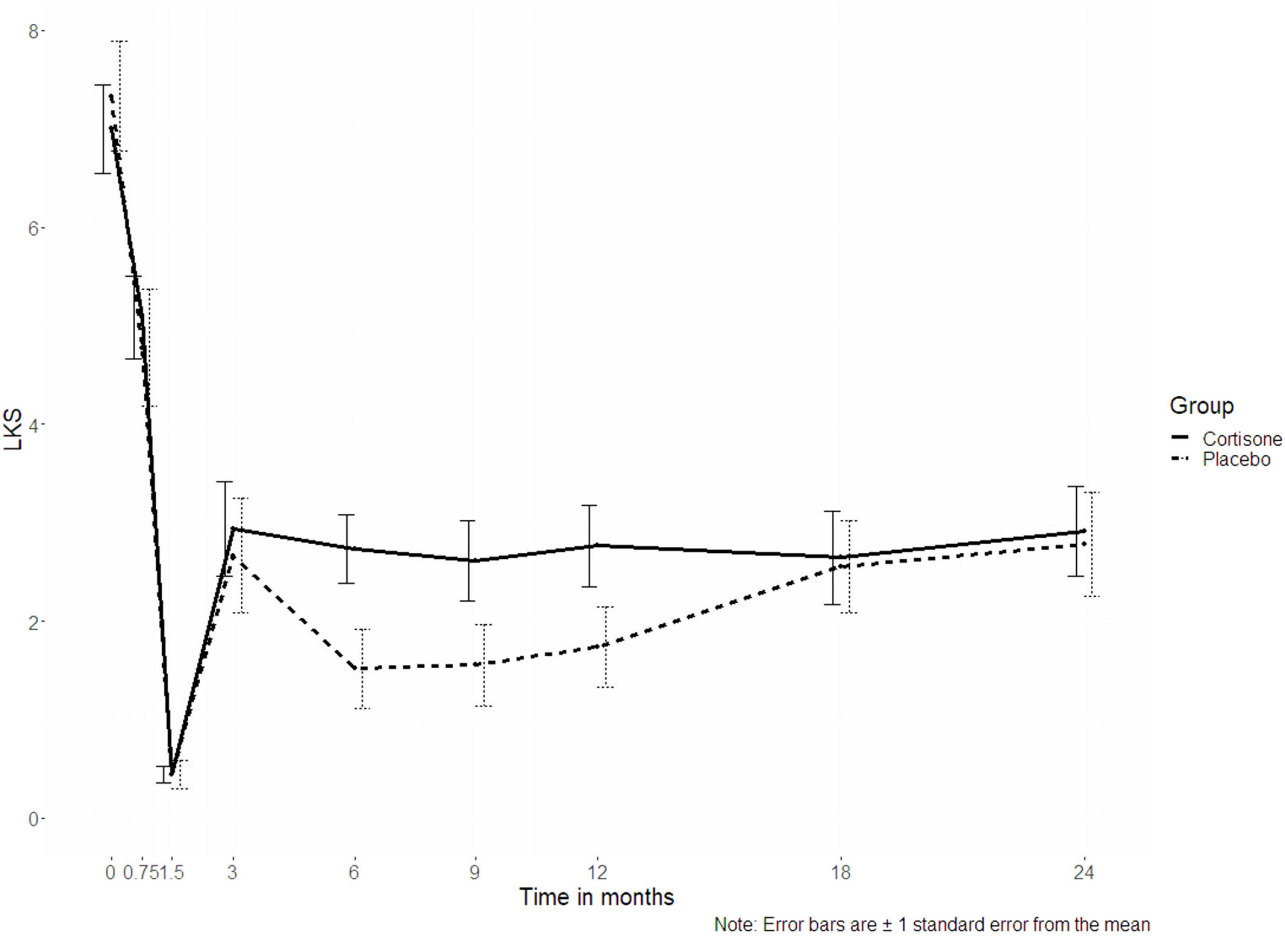
Line plots of the Lund-Kennedy score (LKS, p = 0.507); the dotted line represents the placebo group, the continuous line represents the steroid group.

Concerning the endoscopic secondary outcome measure assessed by the Lund-Kennedy score, no significant interaction “group x time” (p = 0.507) could be seen over all time points (**Figure 3**).

**Figure 4:**
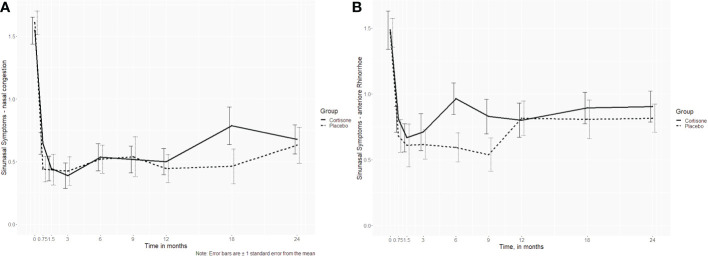
Line plots of the sinonasal symptoms **(A)** nasal congestion (p=0.621) and **(B)** anterior rhinorrhea (p=0.737) over the 2-year follow-up showing no interaction between the steroid and the placebo group; the dotted line represents the placebo group, the continuous line represents the steroid group.

**Figure 5:**
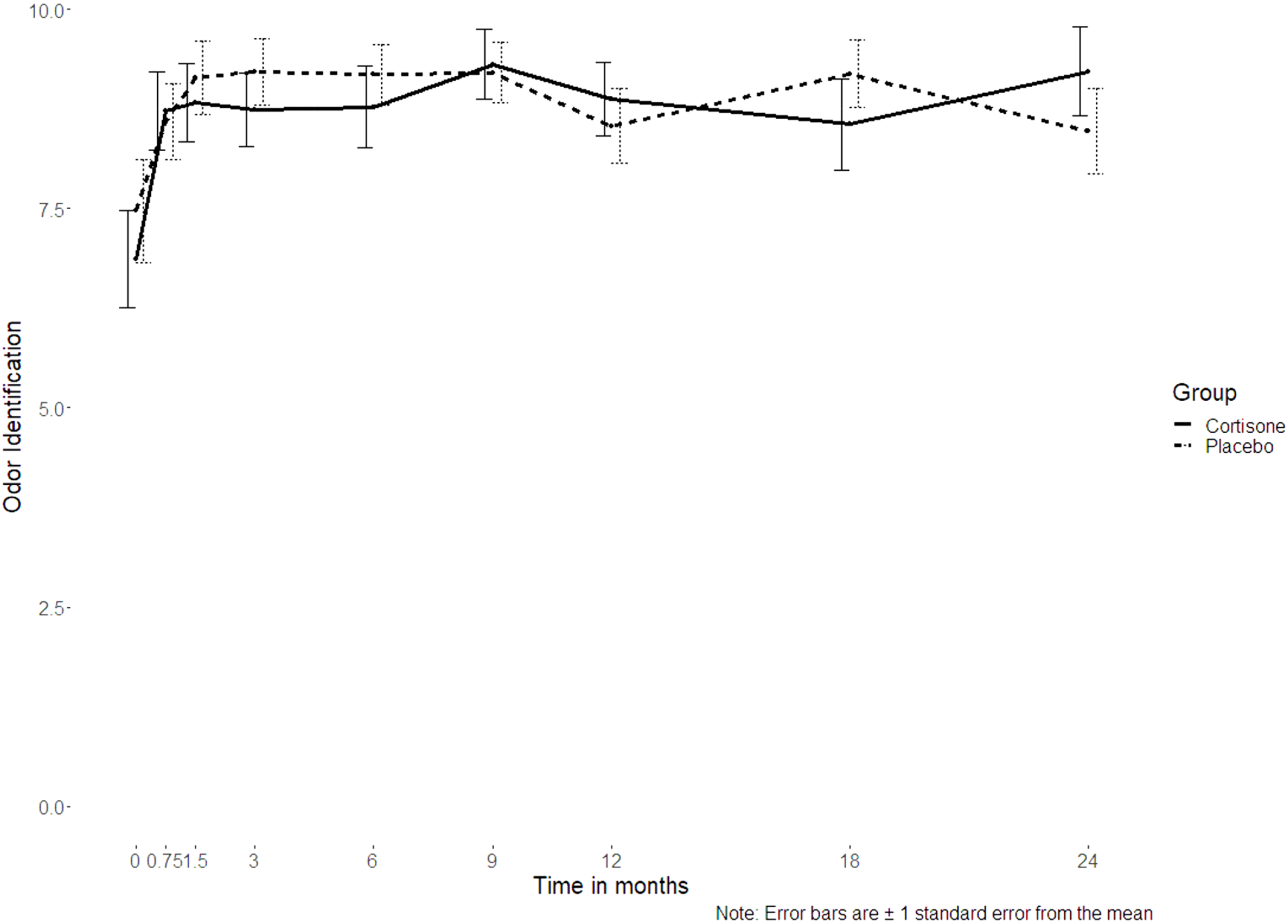
Line plot of the 16-item odor identification test scores assessed using Sniffin’ Sticks over the 2-year follow-up showing no interaction between the steroid and the placebo group (p=0.380); the dotted line represents the placebo group, the continuous line represents the steroid group.

For the sinonasal symptoms (nasal congestion, anterior rhinorrhea, posterior rhinorrhea, sneezing, sense of smell) there was no significant interaction between the groups either (p ≥ 0.253, **Figures 4A, B**). Only for headache was a significant interaction found (p = 0.027), with no differences between the groups at post-hoc level (p ≥ 0.061 if uncorrected/p ≥ 0.547 if corrected for multiple comparisons). Nor did the 16-item odor identification test scores (Sniffin´ Sticks) differ significantly between the groups (p=0.380) (**Figure 5**).

This was also true for most of the biomarkers (CST1, CST2, PAPP-A, Periostin, SerpinE1, SerpinF2, MMP9, IgE, Il-4 and Il-5, with p ≥ 0.078) as well as the general quality of life measures (SF-36: physical functioning p=0.762, role physical p=0.287, pain index p=0.027, general health perceptions p=0.959, vitality p=0.431, social functioning p=0.159, role emotional p=0.566, and mental health p=0.422) that did not differ significantly between the two groups over time. Although only Serpin E1 showed a significant interaction group x time (p = 0.042), no significant group differences were found at any time point (uncorrected: p ≥ 0.060, corrected: p ≥ 0.542. For all details, please see the *supplemental material)*.

Additionally, neither group differed regarding recurrence rates over time, either defined after the NPS (with 7/41 (17.1%) recurrences in the steroid and 7/39 (17.9%) recurrences in the placebo group (p=0.92)) or the RSDI (with 6/41 (14.6%) recurrences and the steroid and 10/39 (25.6%) recurrences in the placebo group (p = 0.22). Eight patients underwent revision surgery for recurrence within the 2-year follow-up. Here again, the need for revision surgery was not significantly different between the groups (steroid group: 2, placebo group: 7, p=0.064).

### 3.3 Additional effect of surgery

Whereas there was no significant difference between the groups, there was a significant change in all primary and secondary outcome measures concerning the effect of surgery (p<0.05 for all between timepoint 0 and 0.75). The effect of the surgical treatment was maintained over the 2-year follow-up (see *supplemental material)*.

### 3.4 Side effects and adverse events

Over the course of the study, there was one patient from the steroid group who suffered hyperglycemia (0.94%), which was counted as a mild adverse event according to the study protocol (steroid group). Two patients received antibiotics as rescue medication due to orbital complications during the follow-up time of 2 years (steroid group:1, placebo:1). The symptoms resolved completely.

### 3.5 Dropouts

A total of n=106 patients were included in the trial and only 80 patients (75.5%) completed the medication regime of 1 month of systemic steroid and 3 months of intranasal steroids. Only n=61 patients of those (76.25%) completed the long-term follow-up. All patients were contacted and asked for reasons for the dropout. The main reasons for dropout were long distance to the test center and the time-consuming study visits (please compare CONSORT diagram **Figure 1** for details).

### 3.6 Subgroup analysis of the severe eosinophilia group

**Figure 6:**
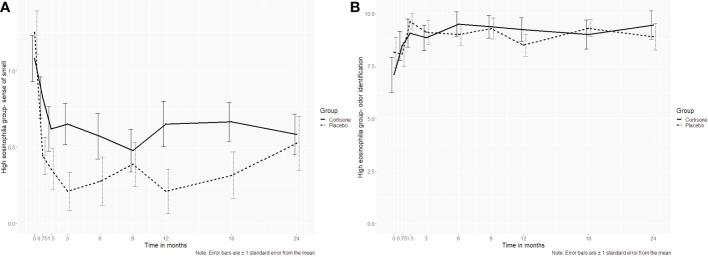
Line plots of the subgroup analysis (high eosinophilia in tissue group) for **(A)** the sinonasal symptom sense of smell and **(B)** the 16-item odor identification test assessed using Sniffin´ Sticks. For the sinonasal symptom sense of smell, a significant interaction (p=0.034) was found with significant differences at the time points 0.75, 3 and 12 months (p=0.042; p= 0.020; p=0.040 respectively). However, after correction for multiple hypotheses testing, the significant differences did not hold. The objective counterpart, the 16-item identification test, did not show a significant difference either (p=0.168). .

A total of n=66 patients (n=29 steroid group, n=35 placebo group) were graded with severe and moderate eosinophilia. There were differences between the steroid and the placebo group concerning all primary and secondary endpoints (p>0.05). For the sinonasal symptom sense of smell, there was a significant interaction (p=0.034) with significant differences in favor of the placebo group at the time points 0.75, 3 and 12 months (p=0.042; p= 0.020; p=0.040 respectively). However, after correction for multiple hypotheses testing, the significant difference did not remain. These results were also underlined by the 16-item identification test scores using Sniffin´ Sticks, where no significant interaction was found (p=0.168)(**Figures 6A, B**).

## 4 Discussion

CRS is associated with adult-onset asthma, significant morbidity, decreased health-related quality of life (HRQoL) (35–39) and a substantial economic burden (2–4, 40). Disease control is poor, even under maximal therapy, with 20% to 80% recurrence rates depending on follow-up duration (41–43). In the preoperative phase, the literature is clear about the value of systemic steroids, which among other things can be used to decrease polyp size and prevent asthma exacerbation. Endoscopic sinus surgery remains the gold standard for refractory cases (1, 8, 9, 15). Previous studies on the effect of systemic steroids in the immediate postoperative phase after FESS (up to 6 months of follow-up) have shown no benefit regarding endoscopic scores and sinonasal symptoms. However, no randomized placebo-controlled trial (RCT) has investigated the impact of postoperative systemic steroids on the 16-item smell identification test, recurrence rates, the need for revision surgery and selected mucus biomarkers. Additionally, no study has investigated a follow-up lasting more than 6 months. This RCT addressed these shortcomings and provided one of the largest sample sizes analyzing the effect of postoperative systemic steroids on the NPS and RSDI (as primary outcome measures) and the LKS, sinonasal symptoms, GNQOL, smell scores, recurrence rates, the need for revision surgery and selected protein biomarkers (as secondary outcome measures).

Several high-quality studies on preoperative topical steroids for CRSwNP show that they are safe and efficient, independent of the steroid and dosage used (44–50). For topical steroids, there was an improvement in SNOT-22 (sinonasal outcome test 22), although the effect was smaller than the minimal clinically important difference (MCID). For the sinonasal symptoms, there was a larger improvement, as was the case for the nasal polyp score. Postoperatively, it could be seen that topical steroids prevented polyp recurrence (51). This proven effect on polyp recurrence raised an ethical concern regarding depriving patients of their postoperative topical steroid spray. Hence, all patients in our study were treated postoperatively with a topical steroid spray for three months. However, we hypothesized, due to the limited distribution of the topical spray to all anatomical sites (52), that the effect of the systemic steroid could still be measured adequately.

Systemic steroid administration is widely used due to the reduction in inflammation, polyp size and the improvement in olfaction. Previous randomized controlled trials (1, 10, 53–56) showed an effect of systemic steroids on disease severity, smell and nasal polyp score. However, this effect had diminished at a follow-up time of 10-12 weeks. Previous randomized controlled studies that directly examined the postoperative effect on systemic steroids were conducted by Shen et al, 2019 (13), Dautremont et al, 2014 (12), Arancibia et al, 2020 (11) and Chang et al, 2021 (14).

In 2019, Shen et al. included patients (n=82) with topical steroid spray (mometasone furoate 100 µg) who were randomized postoperatively between an oral prednisolone course (15 mg twice a day for 2 weeks) and a placebo. At 1, 3 and 6 months, there was no difference in the SNOT-22 score. Concerning the LKS there was a trend towards lower scores at 6 months (p=0.05) in the prednisolone group. For a subgroup with high eosinophils there were significantly lower scores in the prednisolone group at 3 months and a trend at 6 months. However, these results were not corrected for multiple hypotheses testing. Dautremont et al, 2014 included n=36 postoperative patients after FESS. They combined budenoside irrigations (1 mg in 240 ml of saline) and a spacer (soaked with 2 ml of triamcinolone 40 mg/ml) with a randomization to prednisolone 30 mg (daily for 7 days) and a placebo. After their follow-up at 1 week, 3 weeks and 2 months, there were no significant differences in mean LKS or in the SNOT-22 scores.

Arancibia et al (11), included n=70 CRSwNP patients into their trial. A total of n=35 patients were randomized to topical steroids *via* nasal douching plus an oral steroid taper, n=35 patients were randomized to the control group and received only nasal douching. Topical and oral steroids were administered for four weeks. For oral steroids, prednisolone was tapered down from 30 mg. Outcome measures included a total 5-item symptom score, polyp size score, Barcelona Smell Test 24 and Medical Outcome Study Short Form-36 questionnaire for QoL. This study used only a follow-up of 4 weeks. After this time, there were no significant differences in any of the outcome measures between the two groups.

In the recent randomized controlled trial of n=72 patients by Chang et al, 2021 (14) the authors found a postoperative systemic prednisolone taper to be of no benefit in their follow-up of 6 months concerning SNOT-22 and Lund-Kennedy endoscopy scores. They even described a worse outcome in the SNOT-22 psychologic subdomain scores for the systemic steroid group (F[4254] = 3.18, η2 = 0.05 [95% CI, 0.02-0.09].

The results of all previous trials are in accordance with our data. However, the longest follow-up was 6 months. As a consequence, we included patients in our study who had 1) a short-term follow-up up to 9 month and 2) a long-time follow-up of up to 24 months. Interestingly, our data showed a strong and significant effect of surgery. There was a significant change after surgery in the following outcome measures: NPS, the LKS, sinonasal symptoms, SNQoL, GQoL, and smell scores. For the biomarkers, there were also significant changes after surgery. All outcome measures improved after surgery, and levels remained relatively stable up to the end of the follow-up period of 2 years. This improvement is interesting considering that even the topical steroid was discontinued after 3 months. However, this study´s purpose is not to discuss the value of postoperative topical steroid sprays or nasal rinses, although selected data of short-term randomized controlled trials may point towards the fact that its value is decreasing and differences to placebo groups tend to be subtle (50, 57–60).

The physiological actions of prednisolone are well understood. Prednisolone binds to cytosolic receptors forming steroid-receptor complexes, which are activated and translocated into the nucleus. In the nucleus, the complex activates specific nuclear receptors, resulting in an altered gene expression and inhibition of proinflammatory cytokine production (61). Consequently, with systemic prednisolone, we were expecting less edema and crusting in the postoperative phase, an improvement in the healing stages (62) and an increased patency of the sinuses as a result of the above. Consequently, we expected differences in short-term as well as long-term follow-up. However, our data confirmed previous studies to the effect that this was not true for a short-term follow-up and, moreover, our data showed that there was no difference in the long-term follow-up of two years either.

This was the first RCT to analyze recurrence rates over a time period of 2 years. Due to the anti-inflammatory effects of prednisolone and their effect on the coagulation and fibrinolysis pathway (63), we would have expected a difference in recurrence rates. The coagulation and fibrinolysis pathway is one of the most significantly altered pathways in CRSwNP, leading to fibrin deposition and edema. Hence, the attenuation of the coagulation and fibrinolysis pathway may have led to lesser recurrence rates in the steroid group. However, steroid effects are described as dose-dependent and more research is necessary in order to entirely understand the pathophysiology of these mechanisms.

Depending on the condition, the intake of systemic prednisolone has been shown to improve patients´ QoL (64, 65). In our study, additional systemic steroids showed no benefit, and we attribute the improvement in sinonasal symptoms and QoL to the combination of FESS and topical steroids.

Regarding the outcome of the 16-item smell identification test, previous RCTs showed that preoperative oral steroids, topical steroids, as well the combination of the two, lead to an improvement in subjective and objective olfactory outcomes in CRSwNP patients (66). However, oral steroids after FESS failed to improve the olfaction outcome at the 6-month follow-up (67). As this study by Wright et al (67) only included 26 CRSwNP patients, a general conclusion is difficult. Interestingly, studies that showed an improvement in subjective and objective olfaction scores, did not follow their patients up for longer than 8 weeks (56, 68, 69). Our data did not show any benefit after primary FESS in the short-term period up to 8 weeks or at any other follow-up time point until the two-year follow-up. However, it is possible that the additional effect of oral steroids was masked by the strong effect of the surgery.

Several mucus protein biomarkers were selected and analyzed over time. Selection was based on previous studies of our group and evaluation in the literature. PAPP-A, CST1/2 and Periostin were included as they were able to monitor disease severity over time in previous studies and predict early CRSwNP recurrences (22, 32). Pappalysin-1 is a secreted protease that plays an important role in polyp growth as it indirectly releases insulin-like growth factor-1 in the proximity of the insulin-like growth factor receptor 1 (70). Cystatin-SA/SN are cysteine protease inhibitors that enhance eosinophil activation and recruitment through induction of IL-5 and suppress allergic rhinitis symptoms by inhibiting allergenic protease activities and protecting the nasal tight junction barrier in an allergen-specific manner (71, 72). Periostin is a secreted extracellular matrix protein that is important for extracellular matrix restructuring, tissue remodeling, and epithelial-mesenchymal transition, all of which can be related to tissue healing, development, and disease. Thus, it functions as a mediator, balancing appropriate and inappropriate responses to tissue damage (23, 73). SerpinE1 and SerpinF2 are serine protease inhibitors that were included as representatives for the coagulation and fibrinolysis pathway, one of the most significantly altered signaling pathways in CRSwNP as mentioned above. MMP9 was included as a downstream marker of Th2 cytokine signaling that can proteolytically cleave inflammatory mediators (74). MMP9 is a matrix metalloproteinase whose physiological function is the breakdown of the extracellular matrix and thus is involved amongst others in angiogenesis, wound healing and migration. In CRSwNP, the expression of MMP9 is promoted by Il-17A by activating the NF-κB signal pathway (29). IgE and tissue eosinophilia were included as representative markers for Th2 inflammation. IgE are antibodies secreted by plasma cells that, apart from other things, play a role in the IgE-dependent regulation of mast cell function, allergic inflammation and tissue remodeling (75). As Th2 inflammation is more prone to respond to steroids compared to non-Th2 inflammation (1), we performed a subgroup analysis for patients with high tissue eosinophilia. The results of the biomarker analysis showed that periostin, PAPP-A, CST-2, SerpinF2 and eosinophilia showed similar courses to NPS. However, as the NPS, none of the biomarkers showed any significant difference between the steroid and the placebo group. This was also true for the subgroup analysis with high and low tissue eosinophilia. In this subgroup analysis, the subjective sinonasal symptom sense of smell showed a significant interaction with differences at some time points in favor of the placebo group. However, after the p-value was corrected for multiple hypotheses, there was no longer a significant difference. The psychophysical Sniffin´ Sticks test as an objective counterpart did not show a significant interaction either.

Limitations of the study include the strong additional effect of surgery. This may always be the case for the study design of postoperative additional steroids; however, slight postoperative differences may be masked by the effect of surgery. Additionally, the power analysis was as usual made for the primary outcome measure only. The high number of study variables and the associated need for multiple corrections may have prevented the study to detect differences in our study cohort. Therefore, it is possible that the study is still underpowered and that a larger sample size would unmask differences between the steroid and the placebo group. However, mostly effect sizes were small or moderate which means that even a (slightly) larger sample size would not have been detected any differences. To answer this question, future multi-centered studies with a larger sample size are necessary.

Furthermore, we have to look critically at the dropout rate. Only 80/106 patients finished the medication regime and were included into our intention-to-treat analysis. Whereas for some patients of both groups, the dropout was not associated with the disease (e.g. diagnosis of malignancy, diagnosis of inverted papilloma, diabetes) other patients dropped out because of long travel to hospital site or did not show up anymore for no reason. They also did not answer the phone calls or letters. This holds the potential bias that the remaining study group consists of a more uncontrolled group than the dropout group. Although **Table 3** showed that they are similar at baseline, one has to keep this issue in mind. Additionally, here again, the strong effect of surgery may have caused the patients to feel so much better that they did not comply with medication. One may also ask why a follow-up period of 2 years was chosen. Only due to the long-term follow-up could the question as to whether there are differences in early recurrences within the 2-year follow-up between the two groups be answered, and follow-up must be ongoing to assess later recurrences or the need for revision surgery > 2 years. Additionally, additional systemic steroids may have a short-term effect, e.g., on LKS, QoL or smell scores that may level out or even reverse over time and can only be detected with a longer follow-up.

Finally, it is important to mention that in our study only patients without previous surgery were selected in order keep the cohort as homogeneous as possible. Consequently, results may only be applied to CRSwNP patients with primary FESS and not for recurrent surgeries.

For the postoperative setting, the European Position Paper on Rhinosinusitis and Nasal Polyps 2020 (1) did not recommend the use of steroids based on these short-term follow-up studies. Our data confirm these short-term results. Additionally, our data imply that there is no added benefit of systemic steroids in a postoperative setting for patients with primary FESS with a long-term follow-up of 2 years. In our study this was true for all endoscopic, subjective and objective outcome measures. Even in the high eosinophilia group, no benefit could be seen for the steroid group. For now, systemic steroids do not seem to have an additional benefit over FESS plus topical steroids alone in CRSwNP patients undergoing primary surgery. However, although the study design is robust, future multi-centered randomized controlled trials with a larger sample size and a lower dropout rate have to verify the results.

## 5 Conclusions

The addition of postoperative systemic steroids after primary FESS did not show a benefit over topical steroid nasal spray alone with respect to NPS, SNQOL, LKS, GQOL, sinonasal symptoms, smell scores, and biomarkers over a short-term follow-up of up to 9 months and a long-term follow-up of up to 24 months in CRSwNP patients. Simultaneously, the addition of postoperative systemic steroids had no impact on recurrence rates and the need for revision surgery. Functional endoscopic surgery did, however, show a strong effect on all outcome measures, which remained relatively stable up to the endpoint at 2 years. Future studies with a larger sample size have to confirm those results.

## Data availability statement

The original contributions presented in the study are included in the article/**Supplementary Material**. Further inquiries can be directed to the corresponding author.

## Ethics statement

The studies involving human participants were reviewed and approved by Institutional review boards of the Friedrich-Alexander University Erlangen‐Nürnberg (FAU), Philipps University Marburg, Christian-Albrecht University Kiel (CAU), Charité Berlin, Marienhospital Stuttgart and the University of Regensburg (IRB No. 3201 and IRB Nos. No: 4_20; No: 269_17). The patients/participants provided their written informed consent to participate in this study.

## Author contributions

SM and HI collected the data, analyzed the data and wrote the manuscript. OW and AN performed the experiments and reviewed the manuscript. MT, WH, HO, HS, SW, AT, TK, SH, TH, PA, AF, BS, H-WB, MK, HB, KM, PG, AA collected the data and reviewed the manuscript. All authors contributed to the article and approved the submitted version.

## Supplementary material

Supplemental files Line plots of all primary and secondary outcome measures for each of the 9 time points. Appendix: *Protein Biomarker Analysis Using Enzyme Linked Immunosorbent Assay (ELISA).* For PAPP-A, the nasal mucus was measured undiluted. The Human Pappalysin-1 DuoSet No. DY2487-05, R&D SYSTEMS (Bio-Techne GmbH, Wiesbaden, Germany) was used. For Periostin, the nasal mucus was diluted 1:800 with Reagent Diluent (included in the Kit). The Human Periostin DuoSet No. DY3548B, R&D SYSTEMS (Bio-Techne GmbH, Wiesbaden, Germany) was used. For SerpinF2, the nasal mucus was diluted 1:2 with Reagent Diluent. The Human Serpin F2 DuoSet No. DY1470-05, R&D SYSTEMS (Bio-Techne GmbH, Wiesbaden, Germany) was used. For SerpinE1, the nasal mucus was diluted 1:3 with 0.1% BSA in PBS. The Human Serpin E1 ELISA Pair Set, No. SEK10296, Sino Biological Inc. (Beijing, China) was used. For CST2, the nasal mucus was diluted 1:5 with 0.1% BSA in PBS. The Human Cystatin SA/CST2 ELISA Pair Set, No. SEK11567, Sino Biological Inc. (Beijing, China) was used. For CST1, the nasal mucus was diluted 1:200 with Reagent Diluent. The Human CST1 ELISA No. SEK11568, Sino Biological Inc. (Beijing, China) was used. For MMP9, the nasal mucus was diluted 1:800 with Reagent Diluent. The Human MMP9 DuoSet No. DY911, R&D SYSTEMS (Bio-Techne GmbH, Wiesbaden, Germany) was used. For IgE, the nasal mucus was diluted 1:2 with Reagent Diluent. The Human IgE ELISA No. 88-50610, Thermo Fisher (Thermo Fisher Scientic Inc, Waltham, Massachusetts, USA) was used.
