# Supplementary material for: Effect of postoperative systemic prednisolone on short-term and long-term outcomes in chronic rhinosinusitis with nasal polyps: A multi-centered randomized clinical trial
Source: Front Immunol. 2023 Mar 8;14:1075066. doi: 10.3389/fimmu.2023.1075066 (PMC10032209; doi:10.3389/fimmu.2023.1075066)

Supplementary File

NPS

Linear mixed model results

	df1	df2	F	p	R²
Time	8	433.856	81.825	0.000	0.485
Group	1	59.215	0.311	0.579	0.001
Interaction	8	433.856	2.201	0.026	0.010

Descriptive statistics for both groups across time

	M_Placebo	SD_Placebo	N_Placebo	M_Cortisone	SD_Cortisone	N_Cortisone	t	df	p	Holm_p	Cohen d
0	5.68	2.20	28	4.70	2.30	33	1.70	58.14	0.094	0.751	0.44
0.75	0.72	1.02	25	0.42	0.85	31	1.18	46.56	0.244	1.000	0.32
1.5	0.35	0.93	23	0.70	1.30	27	-1.12	46.82	0.266	1.000	-0.31
3	0.77	1.34	26	0.84	1.70	31	-0.17	54.81	0.863	1.000	-0.05
6	0.71	1.33	28	1.07	1.61	28	-0.91	52.15	0.369	1.000	-0.24
9	0.58	0.90	26	0.83	1.42	29	-0.79	48.06	0.433	1.000	-0.21
12	0.43	0.57	28	0.91	1.26	33	-1.97	46.25	0.055	0.498	-0.48
18	0.62	1.02	26	1.18	1.85	28	-1.40	42.74	0.169	1.000	-0.37
24	0.96	1.43	27	1.13	1.88	31	-0.38	55.07	0.704	1.000	-0.10

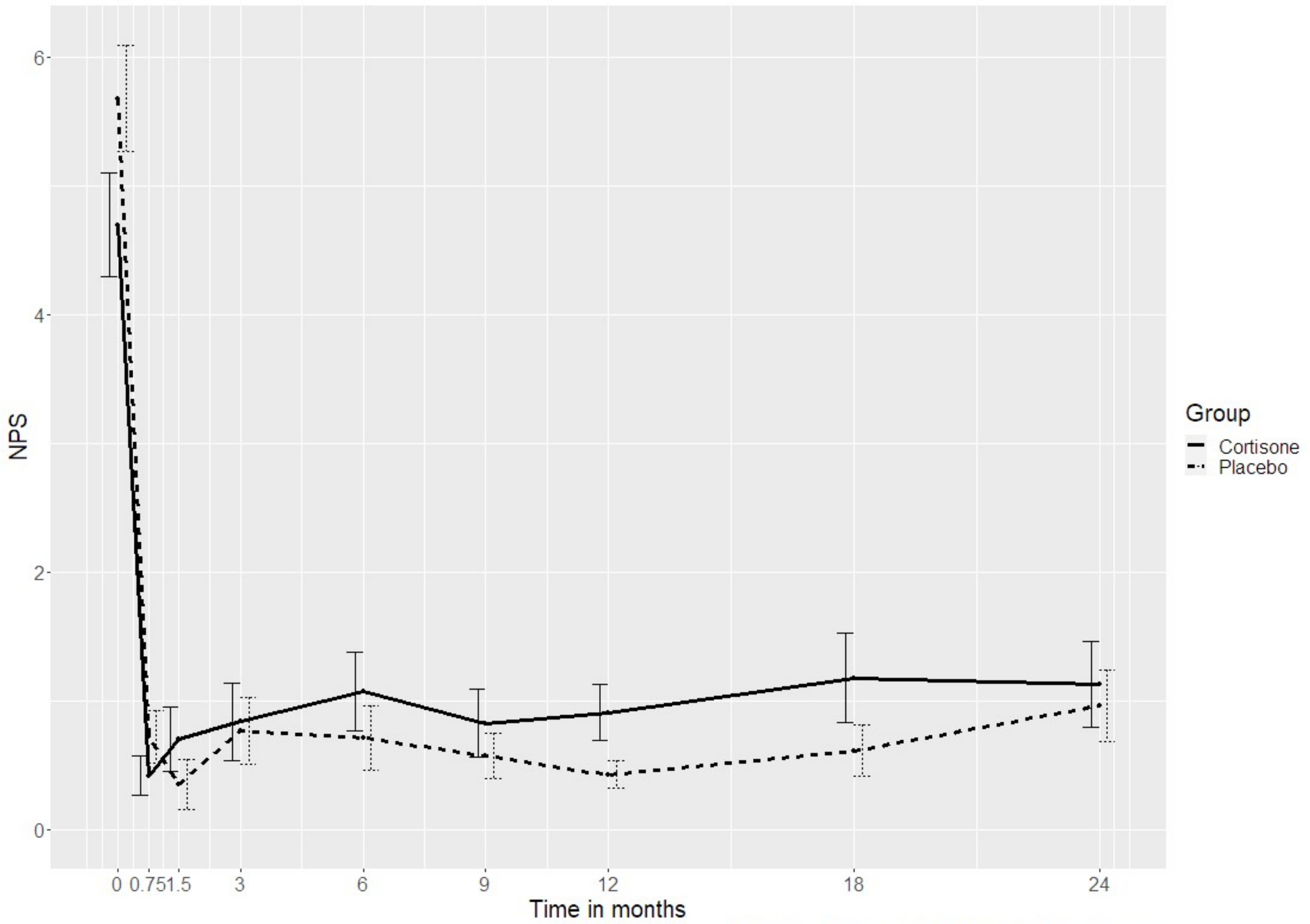

LKS

Linear mixed model results

	df1	df2	F	p	R²
Time	8	472	45.932	0.000	0.359
Group	1	59	1.769	0.189	0.005
Interaction	8	472	0.911	0.507	0.004

Descriptive statistics for both groups across time

	M_Placebo	SD_Placebo	N_Placebo	M_Cortisone	SD_Cortisone	N_Cortisone
0	7.33	2.91	27	7.00	2.63	34
0.75	4.78	3.11	27	5.09	2.44	34
1.5	0.44	0.75	27	0.44	0.50	34
3	2.67	3.01	27	2.94	2.81	34
6	1.52	2.08	27	2.74	2.02	34
9	1.56	2.14	27	2.62	2.37	34
12	1.74	2.12	27	2.76	2.41	34

	M_Placebo	SD_Placebo	N_Placebo	M_Cortisone	SD_Cortisone	N_Cortisone
18	2.56	2.44	27	2.65	2.77	34
24	2.78	2.74	27	2.91	2.68	34

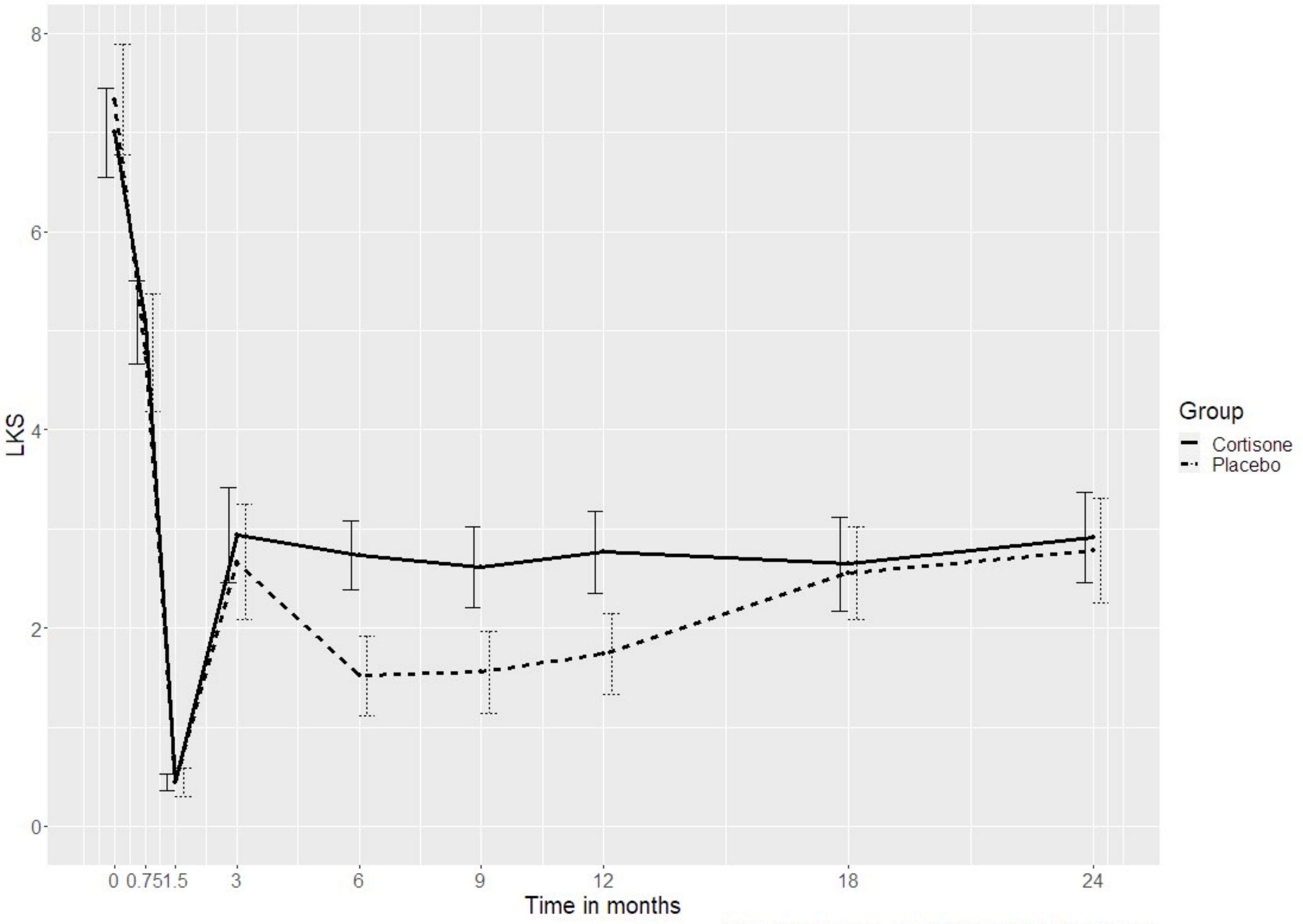

RSDI - Overall

Linear mixed model results

	df1	df2	F	p	R^2
Time	8	465.479	30.301	0.000	0.177
Group	1	67.170	0.529	0.470	0.004
Interaction	8	465.479	0.966	0.462	0.004

Descriptive statistics for both groups across time

	M_Placebo	SD_Placebo	N_Placebo	M_Cortisone	SD_Cortisone	N_Cortisone
0	62.55	23.78	38	67.73	23.41	41
0.75	48.94	20.49	36	51.95	16.07	39
1.5	44.45	17.02	33	43.29	12.81	35
3	43.03	17.59	33	40.47	14.37	38

	M_Placebo	SD_Placebo	N_Placebo	M_Cortisone	SD_Cortisone	N_Cortisone
6	41.58	16.13	36	40.94	13.33	36
9	42.53	18.26	30	42.91	13.60	32
12	39.88	13.37	32	42.00	12.68	34
18	39.23	14.09	30	45.39	20.77	31
24	38.90	14.84	30	42.97	19.07	33

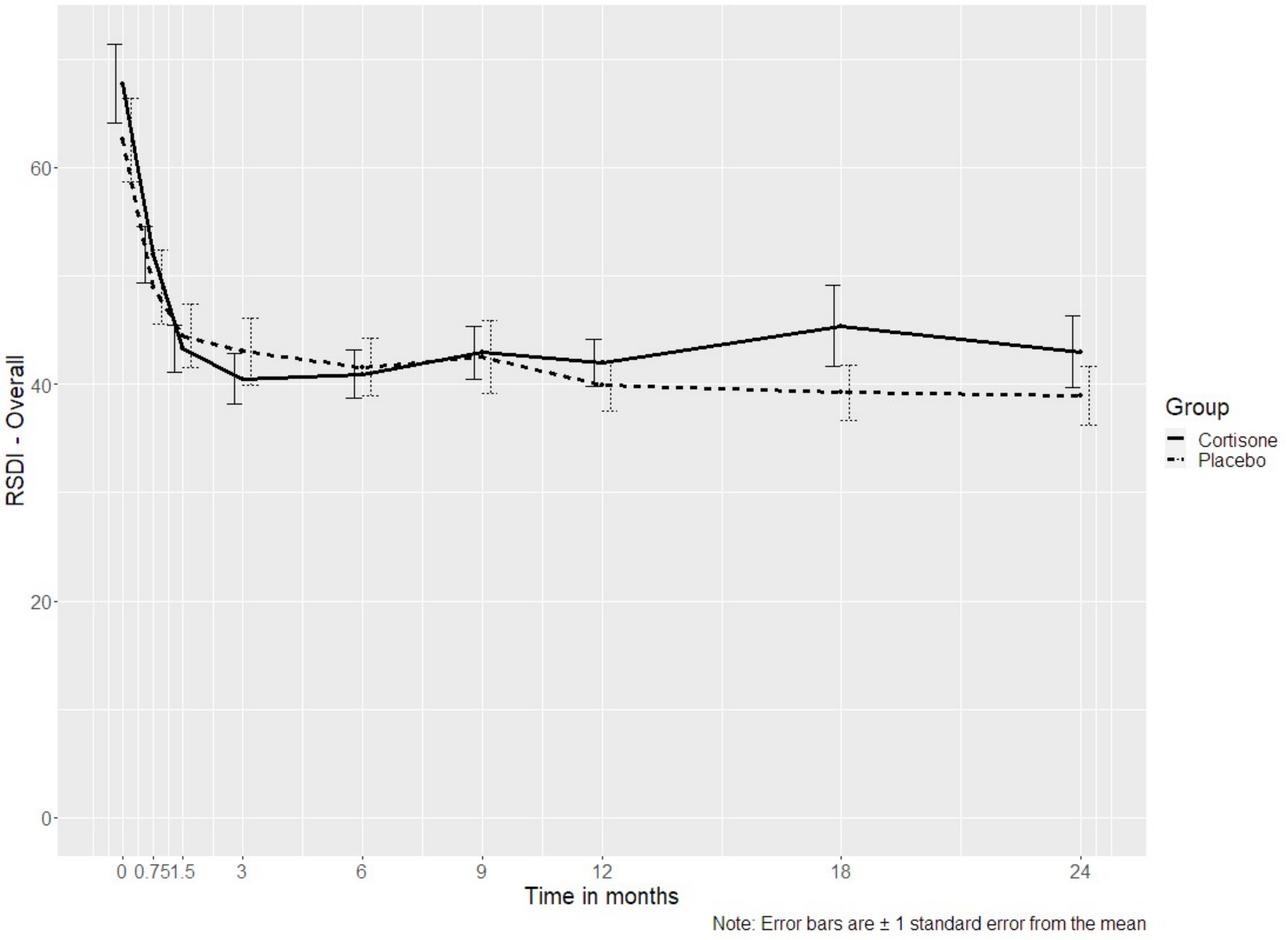

RSDI - Functional

Linear mixed model results

	df1	df2	F	p	R^2
Time	8	465.748	25.349	0.000	0.155
Group	1	67.340	0.597	0.442	0.004
Interaction	8	465.748	1.137	0.337	0.006

Descriptive statistics for both groups across time

	M_Placebo	SD_Placebo	N_Placebo	M_Cortisone	SD_Cortisone	N_Cortisone
0	20.66	7.67	38	21.90	7.64	41

	M_Placebo	SD_Placebo	N_Placebo	M_Cortisone	SD_Cortisone	N_Cortisone
0.75	15.97	6.34	36	17.51	5.92	39
1.5	14.39	5.73	33	14.20	4.54	35
3	14.45	6.11	33	13.32	5.51	38
6	13.97	5.93	36	13.19	4.47	36
9	13.77	5.53	30	14.19	5.03	32
12	13.03	4.29	32	14.06	4.79	34
18	13.20	5.04	30	15.16	7.37	31
24	13.03	5.33	30	14.18	6.59	33

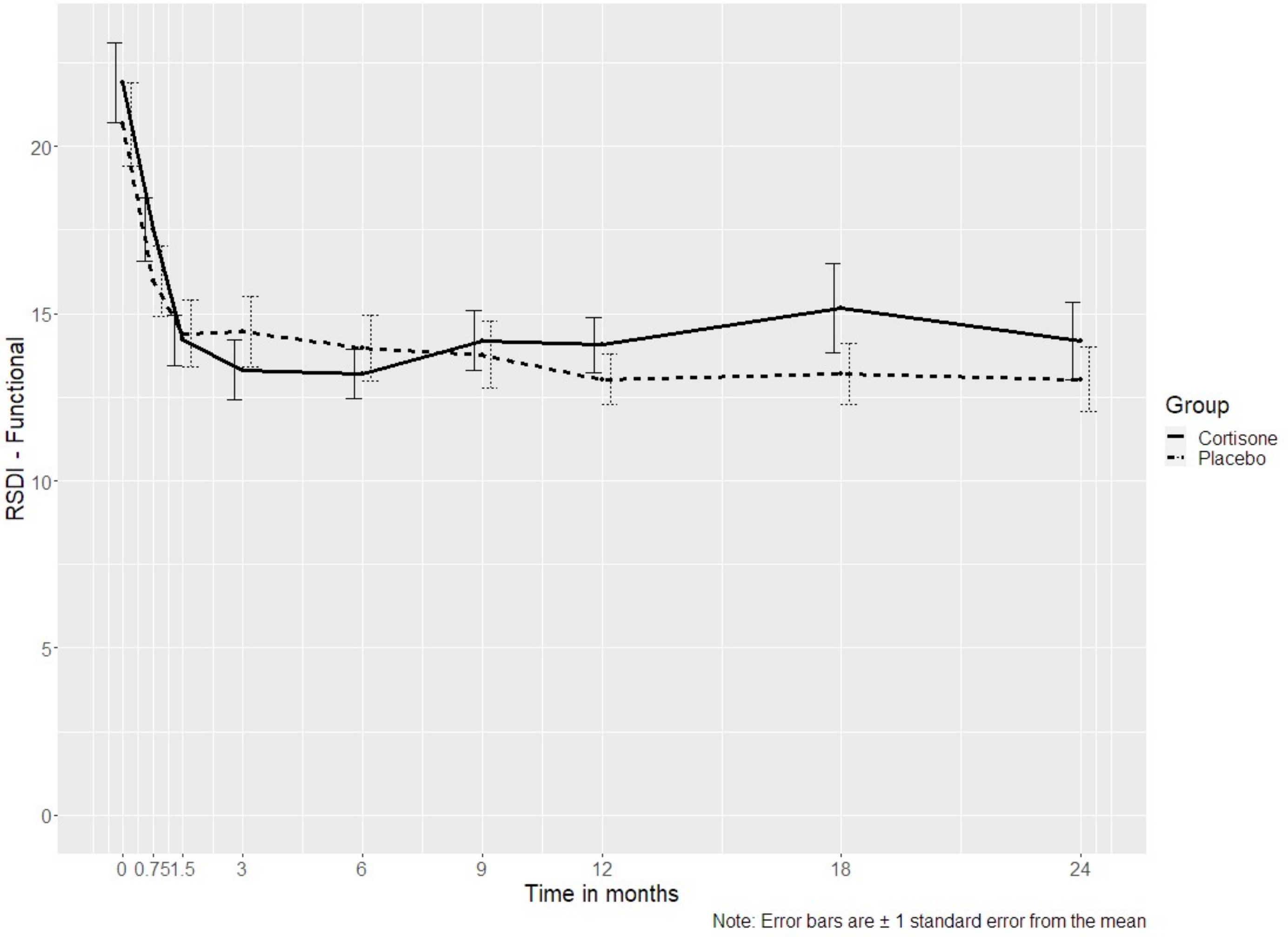

RSDI - Emotional

Linear mixed model results

	df1	df2	F	p	R^2
Time	8	465.123	14.932	0.000	0.099
Group	1	66.675	0.106	0.746	0.001
Interaction	8	465.123	0.744	0.653	0.004

Descriptive statistics for both groups across time

	M_Placebo	SD_Placebo	N_Placebo	M_Cortisone	SD_Cortisone	N_Cortisone
0	17.45	7.95	38	18.93	8.42	41
0.75	14.42	6.30	36	14.56	4.90	39
1.5	13.73	5.73	33	13.09	4.43	35
3	13.27	5.42	33	12.16	4.23	38
6	12.92	4.78	36	12.31	4.35	36
9	13.07	5.79	30	13.00	4.11	32
12	12.19	4.25	32	12.44	3.76	34
18	12.37	4.57	30	13.77	7.50	31
24	12.17	4.90	30	13.15	6.77	33

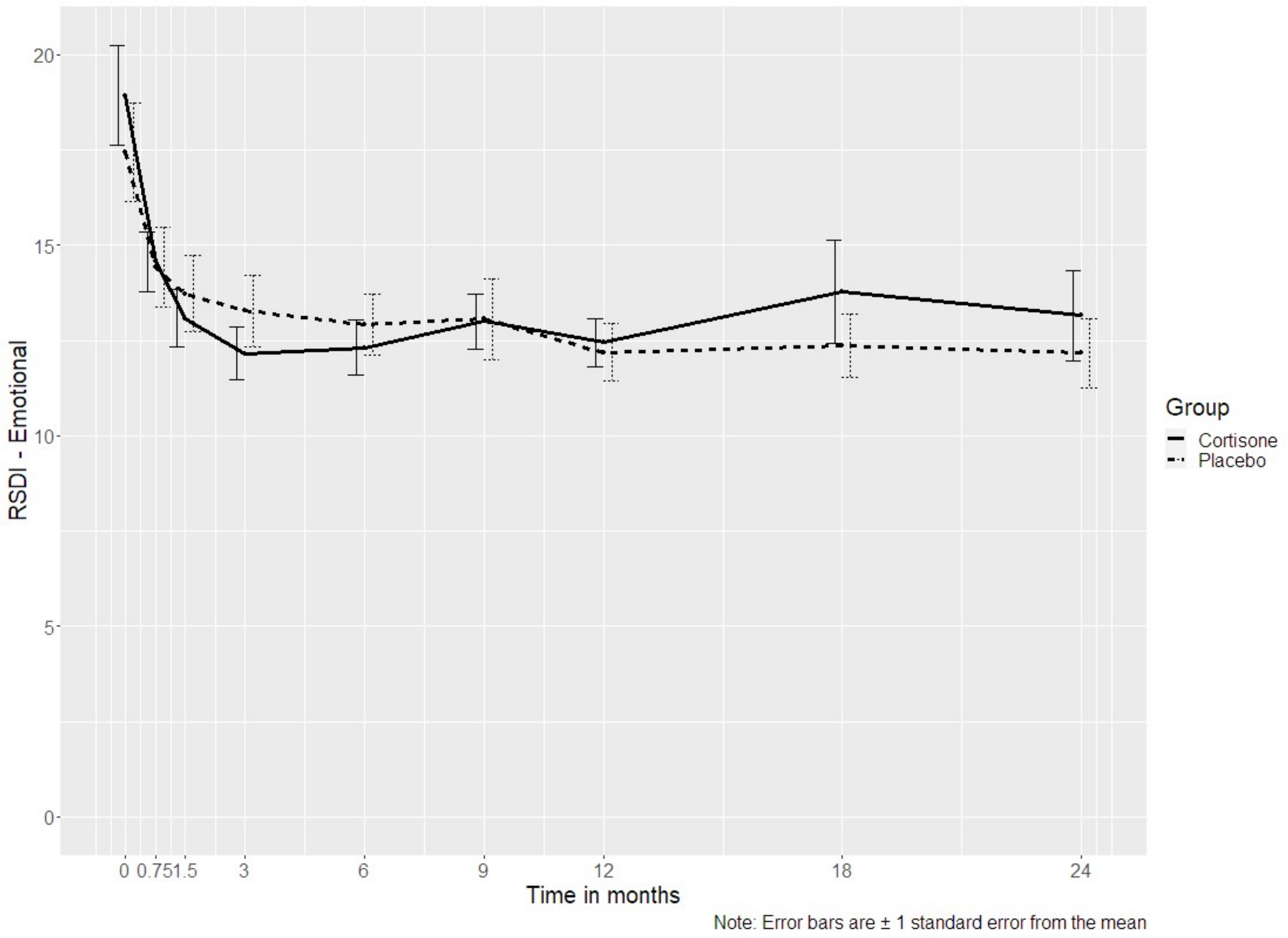

RSDI - Physical

Linear mixed model results

	df1	df2	F	p	R^2
Time	8	465.519	33.220	0.000	0.191

	df1	df2	F	p	R²
Group	1	67.196	0.936	0.337	0.007
Interaction	8	465.519	1.086	0.371	0.005

Descriptive statistics for both groups across time

	M_Placebo	SD_Placebo	N_Placebo	M_Cortisone	SD_Cortisone	N_Cortisone
0	21.26	8.39	38	23.63	8.29	41
0.75	17.08	7.75	36	18.21	6.08	39
1.5	15.09	5.89	33	15.06	4.58	35
3	14.48	6.01	33	14.03	5.04	38
6	13.75	5.73	36	14.25	4.88	36
9	14.47	6.51	30	14.72	5.09	32
12	13.78	5.12	32	14.26	4.32	34
18	12.60	4.10	30	15.13	6.34	31
24	12.73	4.54	30	14.48	6.19	33

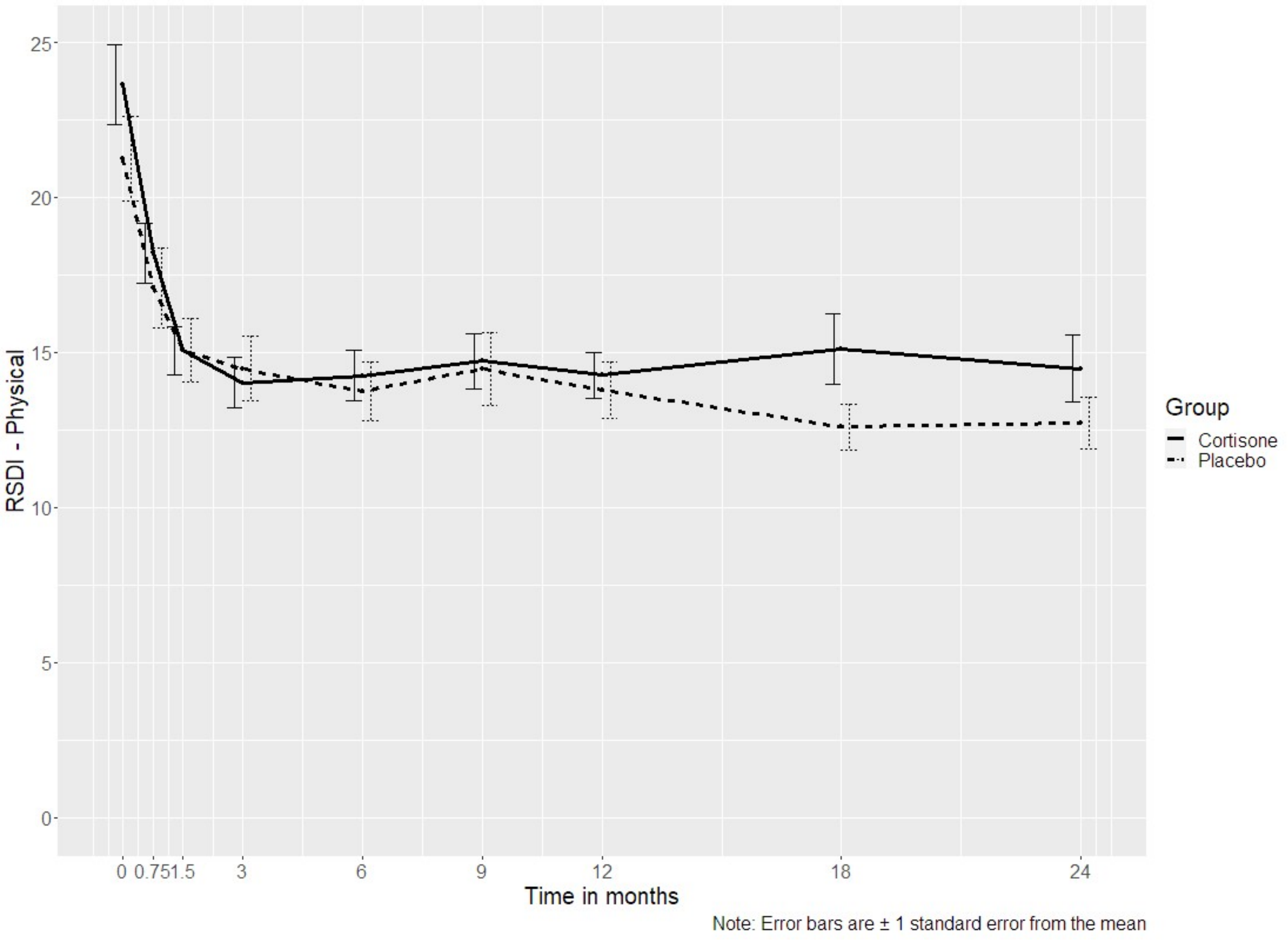

Sinunasal Symptoms - anterior rhinorrhea

Linear mixed model results

	df1	df2	F	p	R^2
Time	8	428.126	12.503	0.000	0.122
Group	1	58.480	0.820	0.369	0.005
Interaction	8	428.126	0.648	0.737	0.005

Descriptive statistics for both groups across time

	M_Placebo	SD_Placebo	N_Placebo	M_Cortisone	SD_Cortisone	N_Cortisone
0	1.46	0.58	28	1.48	0.83	33
0.75	0.68	0.63	25	0.81	0.54	31
1.5	0.61	0.78	23	0.67	0.55	27
3	0.62	0.57	26	0.71	0.78	31
6	0.59	0.57	27	0.96	0.64	28
9	0.54	0.65	26	0.83	0.71	29
12	0.81	0.68	27	0.80	0.71	30
18	0.81	0.75	26	0.89	0.63	28
24	0.81	0.56	27	0.90	0.65	31

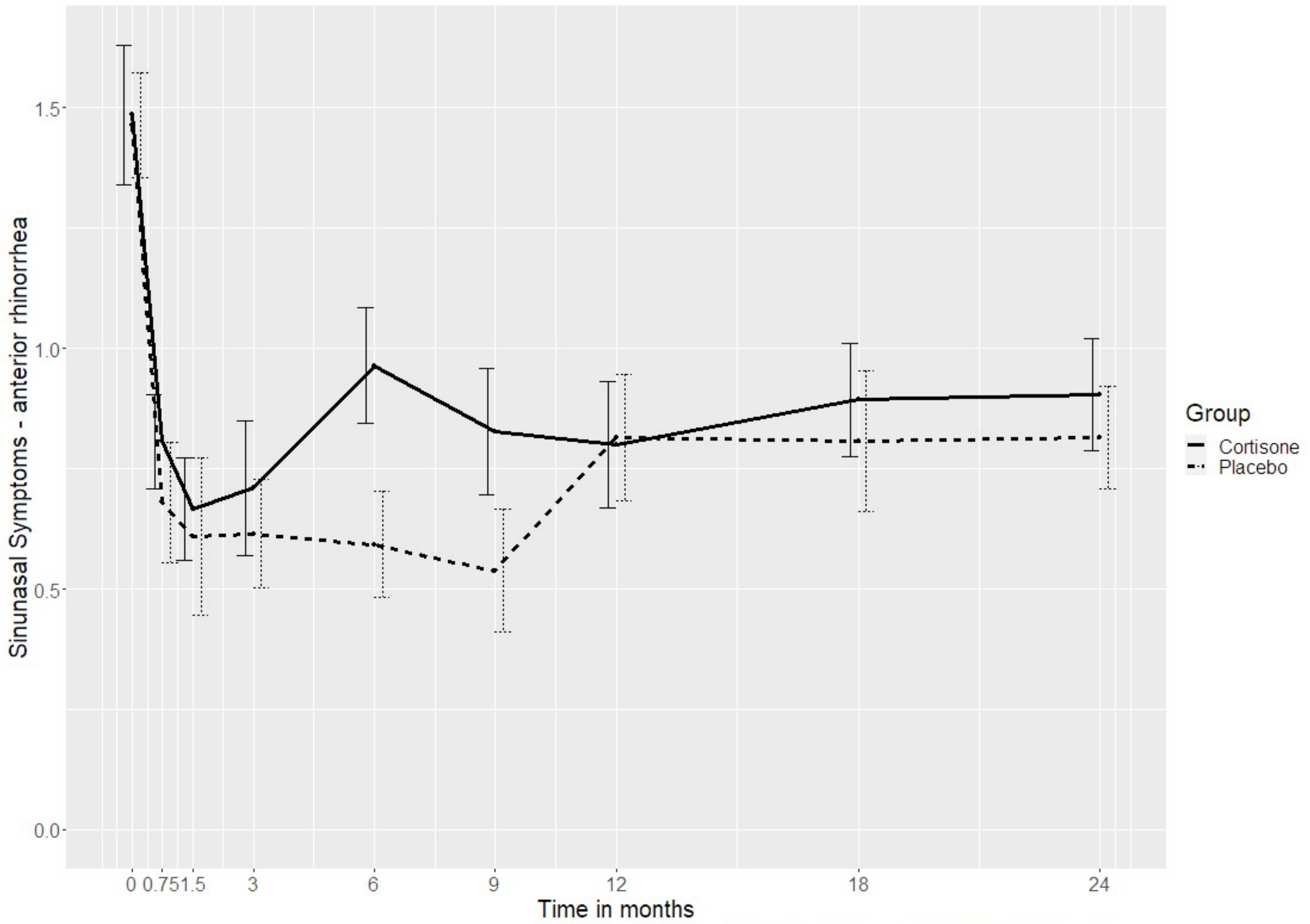

Note: Error bars are ± 1 standard error from the mean

Sinunasal Symptoms - nasal congestion

Linear mixed model results

	df1	df2	F	p	R²
Time	8	429.414	30.125	0.000	0.249
Group	1	59.901	0.243	0.624	0.001
Interaction	8	429.414	0.779	0.621	0.005

Descriptive statistics for both groups across time

	M_Placebo	SD_Placebo	N_Placebo	M_Cortisone	SD_Cortisone	N_Cortisone
0	1.61	0.50	28	1.55	0.62	33
0.75	0.44	0.51	25	0.65	0.49	31
1.5	0.43	0.59	23	0.44	0.51	27
3	0.42	0.58	26	0.39	0.56	31
6	0.52	0.58	27	0.54	0.58	28
9	0.54	0.81	26	0.52	0.57	29
12	0.44	0.58	27	0.50	0.57	30

	M_Placebo	SD_Placebo	N_Placebo	M_Cortisone	SD_Cortisone	N_Cortisone
18	0.46	0.71	26	0.79	0.79	28
24	0.63	0.74	27	0.68	0.65	31

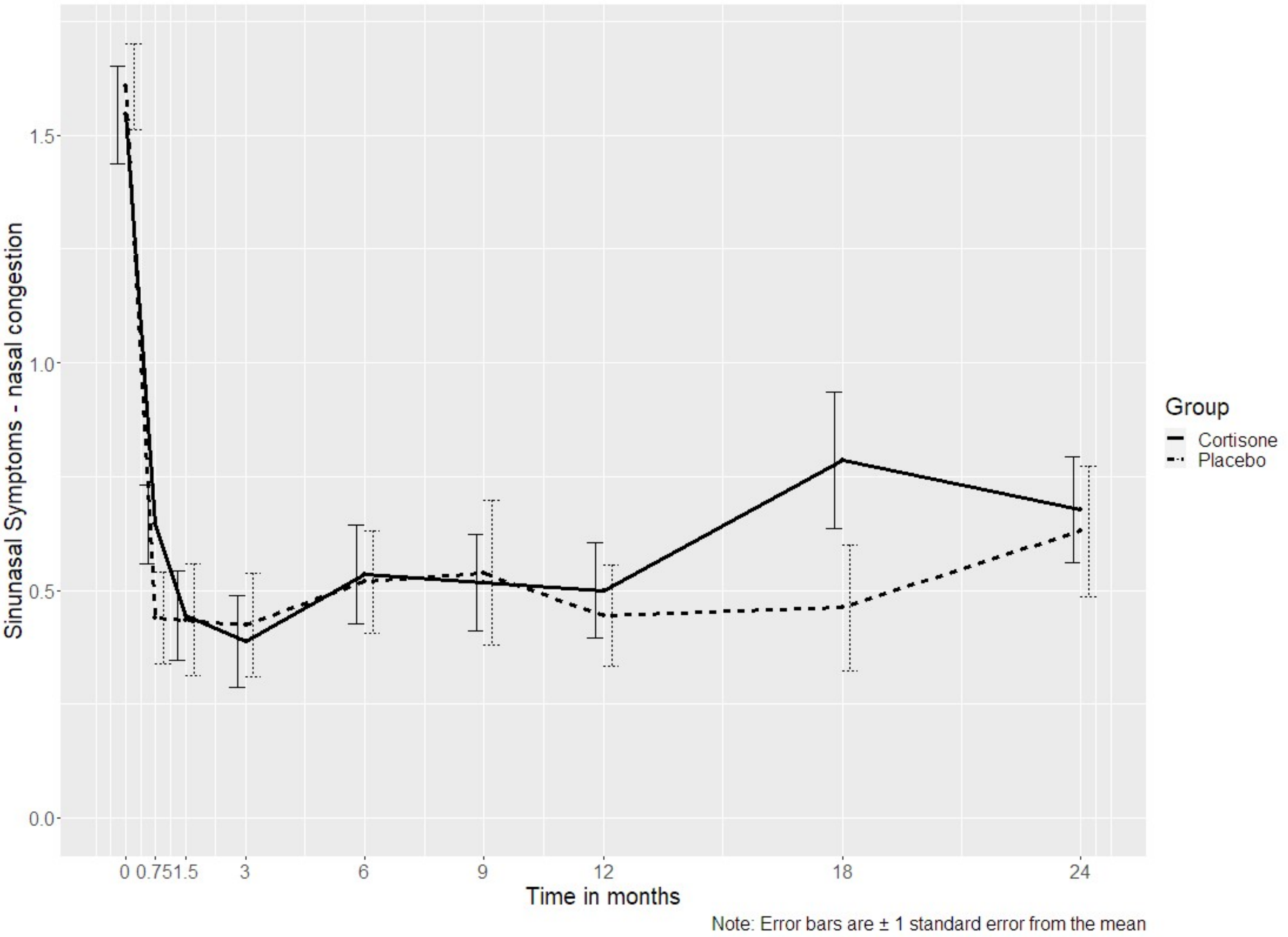

Sinunasal Symptoms - headache

Linear mixed model results

	df1	df2	F	p	R²
Time	8	427.433	6.059	0.000	0.056
Group	1	58.569	0.116	0.735	0.001
Interaction	8	427.433	2.189	0.027	0.018

Descriptive statistics for both groups across time

	M_Placebo	SD_Placebo	N_Placebo	M_Cortisone	SD_Cortisone	N_Cortisone	t	df	p	Holm_p	Cohen d
0	1.04	0.88	28	0.70	0.77	33	1.58	54.14	0.119	0.951	0.41
0.75	0.84	0.94	25	1.13	0.88	31	-1.17	50.00	0.247	1.000	-0.32
1.5	0.52	0.67	23	0.44	0.64	27	0.42	46.12	0.679	1.000	0.12

	M_Placebo	SD_Placebo	N_Placebo	M_Cortisone	SD_Cortisone	N_Cortisone	t	df	p	Holm_p	Cohen d
3	0.54	0.71	26	0.39	0.62	31	0.85	50.06	0.397	1.000	0.23
6	0.44	0.58	27	0.54	0.69	28	-0.53	51.92	0.597	1.000	-0.14
9	0.46	0.58	26	0.62	0.78	29	-0.87	51.48	0.390	1.000	-0.23
12	0.48	0.70	27	0.47	0.68	30	0.08	54.03	0.936	1.000	0.02
18	0.58	0.81	26	0.50	0.64	28	0.39	47.56	0.701	1.000	0.11
24	0.78	0.93	27	0.35	0.71	31	1.92	48.16	0.061	0.547	0.52

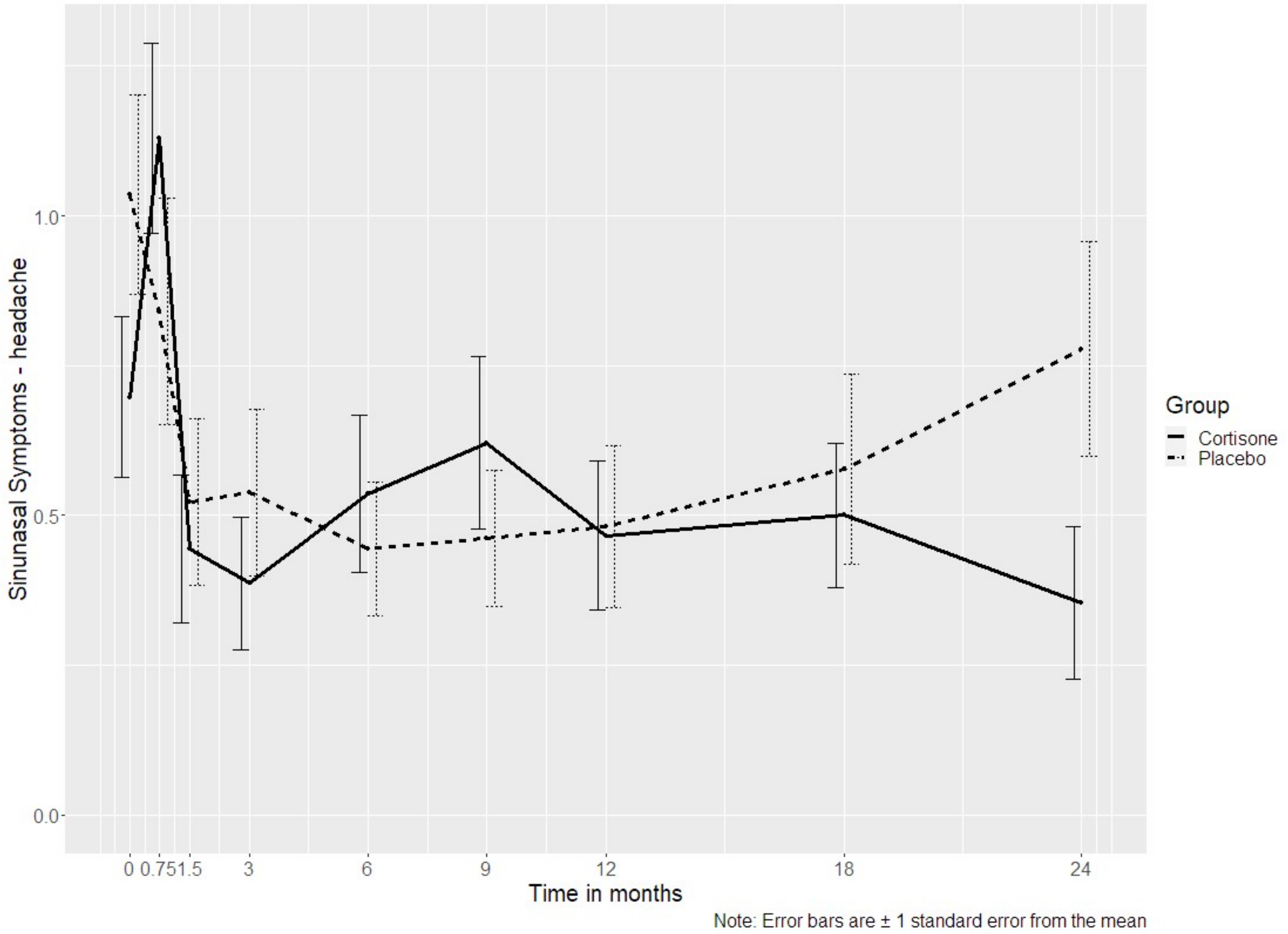

Sinunasal Symptoms - sneezing

Linear mixed model results

	df1	df2	F	p	R^2
Time	8	426.934	7.051	0.000	0.070
Group	1	57.596	0.047	0.829	0.000
Interaction	8	426.934	1.277	0.253	0.012

Descriptive statistics for both groups across time

	M_Placebo	SD_Placebo	N_Placebo	M_Cortisone	SD_Cortisone	N_Cortisone
0	1.21	0.74	28	1.36	0.70	33
0.75	0.84	0.62	25	0.71	0.64	31
1.5	0.78	0.60	23	0.89	0.58	27
3	1.15	0.61	26	0.87	0.56	31
6	0.59	0.84	27	0.79	0.63	28
9	0.58	0.64	26	0.86	0.69	29
12	0.81	0.74	27	0.87	0.51	30
18	0.81	0.90	26	0.86	0.65	28
24	0.81	0.68	27	0.77	0.56	31

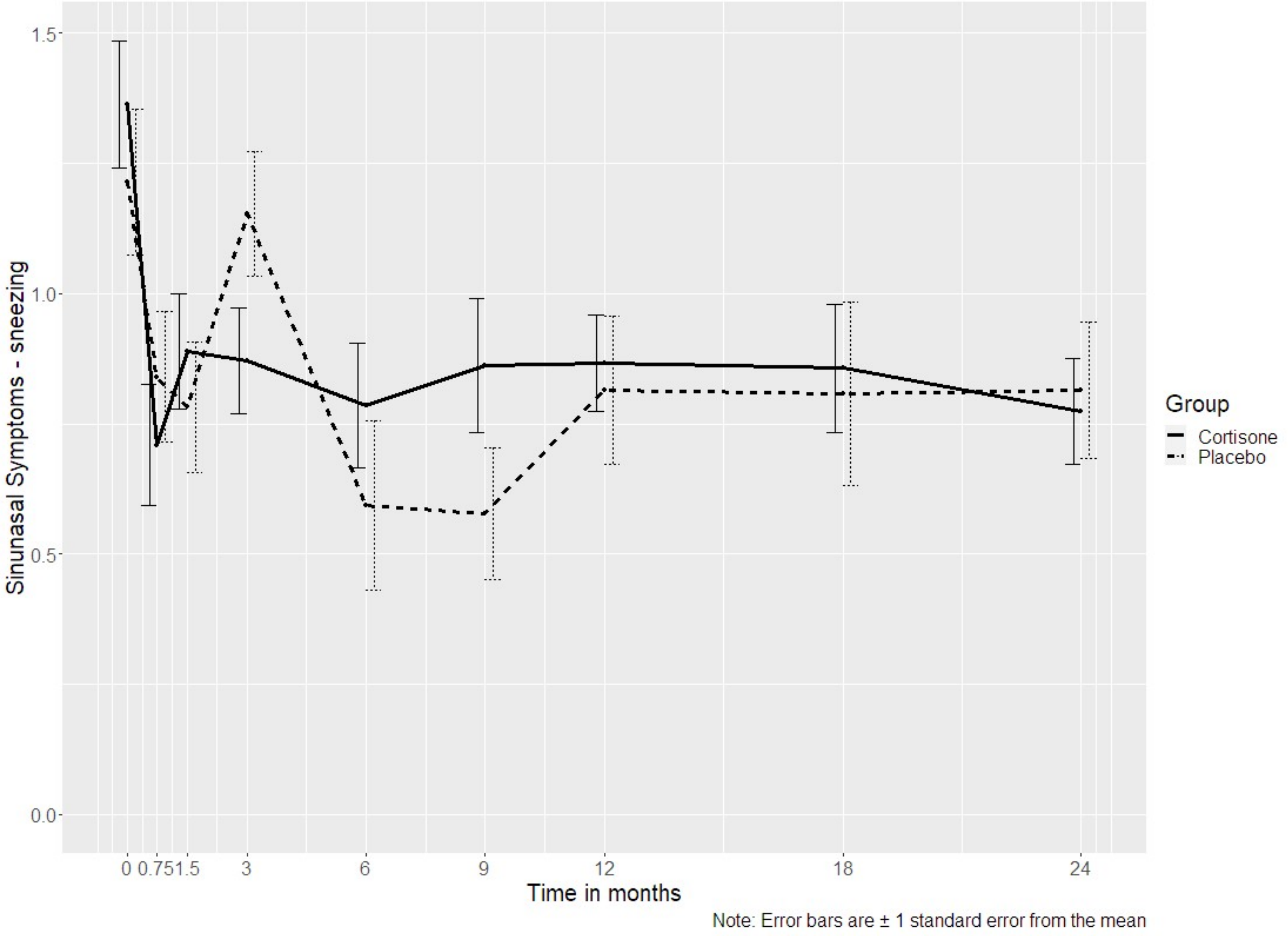

Sinunasal Symptoms - posterior rhinorrhea

Linear mixed model results

	df1	df2	F	p	R²
Time	8	426.060	4.149	0.000	0.035

	df1	df2	F	p	R²
Group	1	58.488	0.521	0.473	0.004
Interaction	8	426.060	0.851	0.558	0.007

Descriptive statistics for both groups across time

	M_Placebo	SD_Placebo	N_Placebo	M_Cortisone	SD_Cortisone	N_Cortisone
0	1.14	0.76	28	1.36	0.99	33
0.75	0.64	0.64	25	0.81	0.60	31
1.5	0.61	0.78	23	0.93	0.92	27
3	0.92	0.93	26	0.90	0.76	30
6	0.63	0.74	27	1.00	0.72	28
9	0.81	0.90	26	0.86	0.74	29
12	0.89	0.85	27	0.83	0.79	30
18	0.77	0.82	26	1.04	0.84	28
24	0.85	0.77	27	0.90	0.75	31

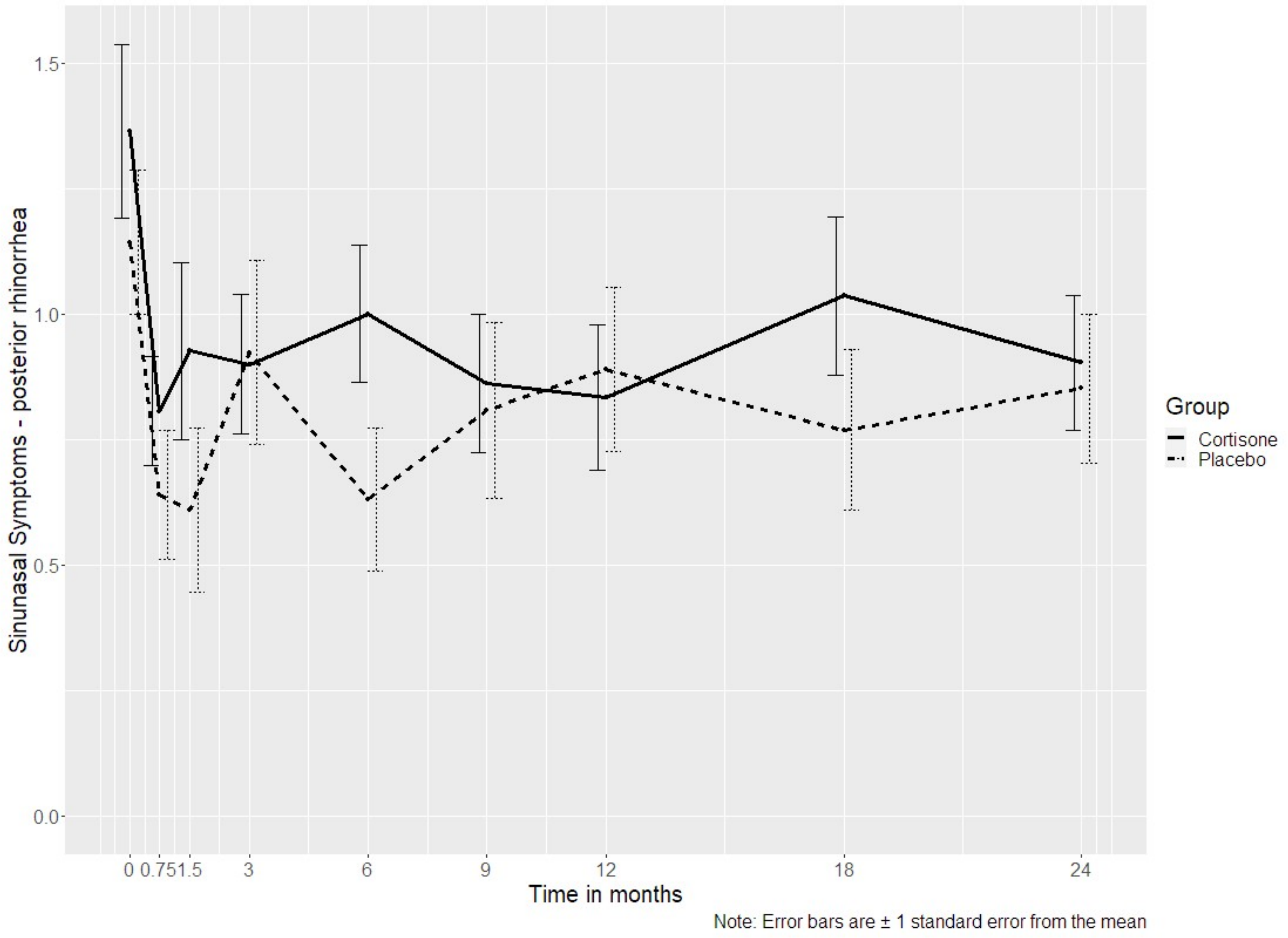

Sinunasal Symptoms - sense of smell

Linear mixed model results

	df1	df2	F	p	R^2
Time	8	426.502	12.238	0.000	0.077
Group	1	58.410	3.198	0.079	0.031
Interaction	8	426.502	0.655	0.731	0.003

Descriptive statistics for both groups across time

	M_Placebo	SD_Placebo	N_Placebo	M_Cortisone	SD_Cortisone	N_Cortisone
0	0.96	0.69	28	1.24	0.75	33
0.75	0.52	0.59	25	0.74	0.73	31
1.5	0.57	0.66	23	0.63	0.74	27
3	0.42	0.58	26	0.58	0.76	31
6	0.33	0.55	27	0.57	0.74	28
9	0.31	0.55	26	0.69	0.71	29
12	0.37	0.69	27	0.57	0.68	30
18	0.35	0.56	26	0.71	0.76	28
24	0.44	0.64	27	0.74	0.77	31

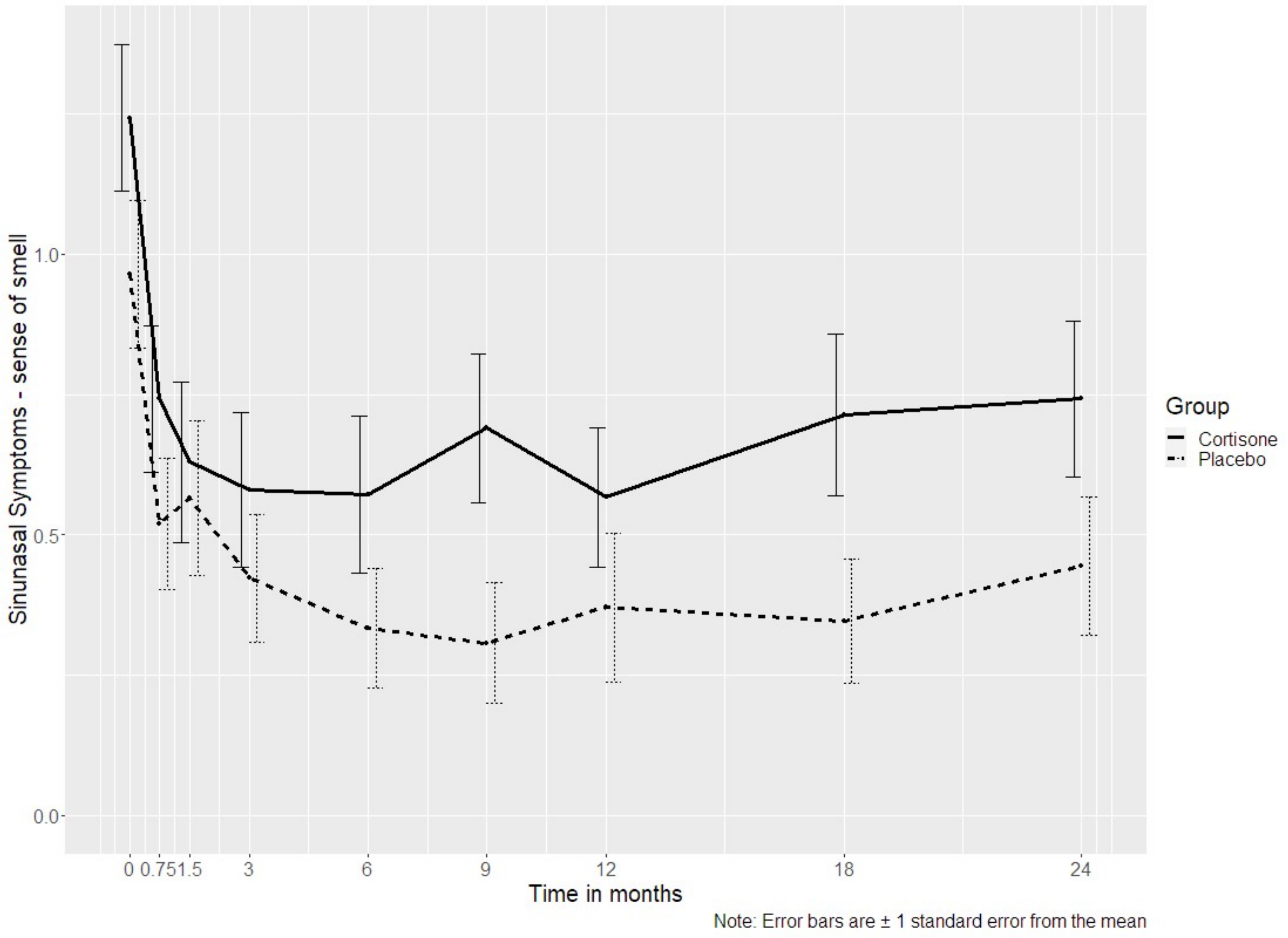

Odor Identification

Overall model (0-24 months)

	df1	df2	F	p	R^2
Time	8	411.668	4.989	0.000	0.034
Group	1	67.944	1.020	0.316	0.010
Interaction	8	411.668	1.084	0.373	0.006

Descriptive statistics for both groups across time

	M_Placebo	SD_Placebo	N_Placebo	M_Cortisone	SD_Cortisone	N_Cortisone
0	7.47	3.64	32	6.86	3.74	37
0.75	8.59	2.56	29	8.72	2.76	32
1.5	9.14	2.47	29	8.83	2.66	29
3	9.21	2.20	28	8.74	2.67	34
6	9.18	2.17	33	8.77	2.85	31
9	9.20	2.07	30	9.31	2.22	26
12	8.54	2.46	28	8.87	2.58	31

	M_Placebo	SD_Placebo	N_Placebo	M_Cortisone	SD_Cortisone	N_Cortisone
18	9.19	2.20	27	8.55	3.09	29
24	8.46	2.82	28	9.22	3.17	32

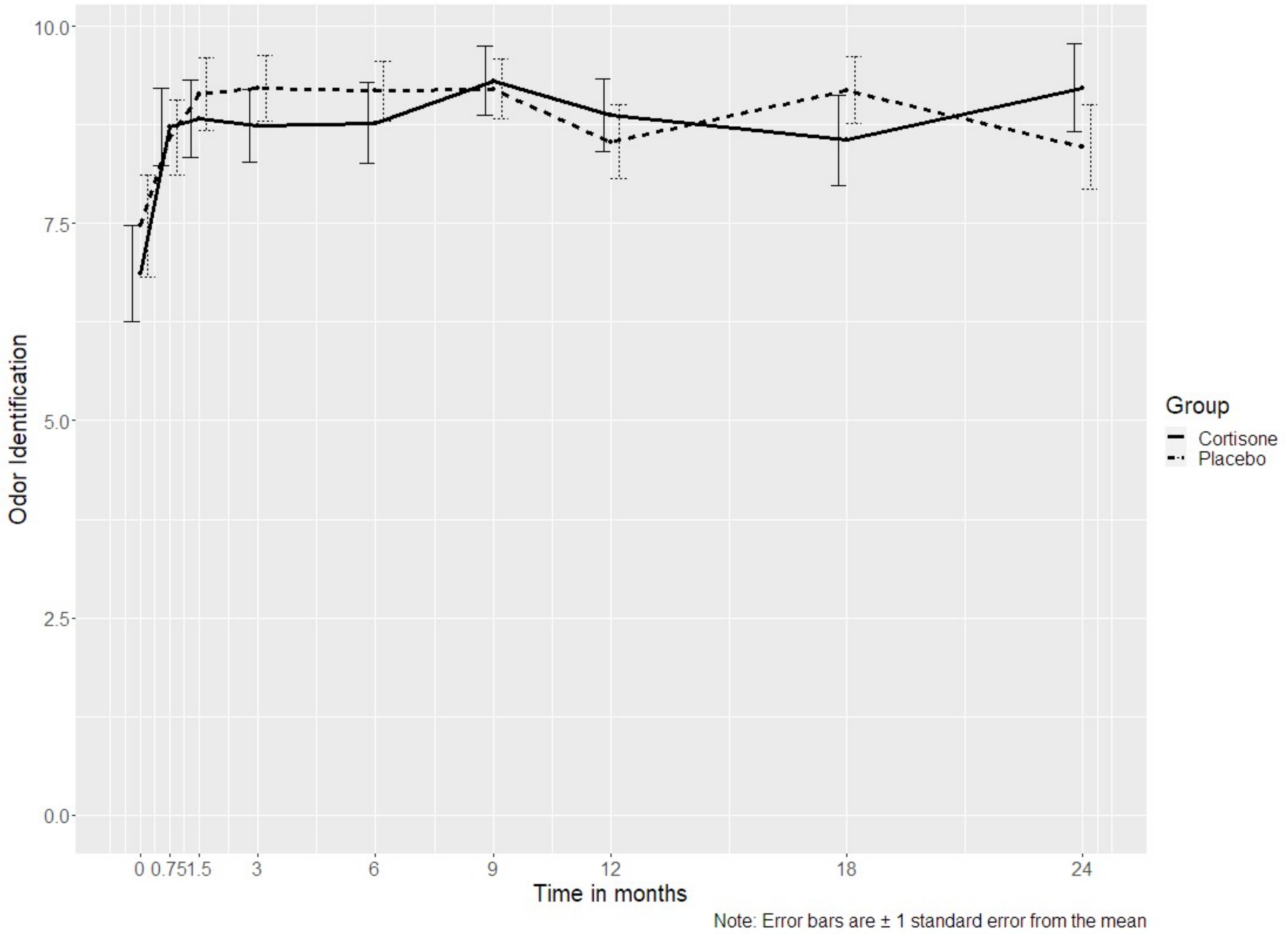

Mucus Serpin E1

Overall model (0-24 months)

	df1	df2	F	p	R²
Time	8	306.327	45.789	0.000	0.426
Group	1	43.570	5.535	0.023	0.020
Interaction	8	306.327	2.040	0.042	0.014

Descriptive statistics for both groups across time

	M_Placebo	SD_Placebo	N_Placebo	M_Cortisone	SD_Cortisone	N_Cortisone	t	df	p	Holm_p	Cohen d
0	160.18	124.71	21	199.80	185.12	22	-0.83	36.95	0.414	0.848	-0.25
0.75	680.50	423.58	19	1015.53	618.25	19	-1.95	31.85	0.060	0.542	-0.63
1.5	354.04	205.59	19	494.20	324.15	19	-1.59	30.46	0.122	0.848	-0.52

	M_Placebo	SD_Placebo	N_Placebo	M_Cortisone	SD_Cortisone	N_Cortisone	t	df	p	Holm_p	Cohen d
3	240.31	149.93	19	326.79	269.45	21	-1.27	31.88	0.213	0.848	-0.39
6	183.82	124.52	22	245.16	140.24	20	-1.49	38.23	0.144	0.848	-0.46
9	163.59	192.43	19	213.56	129.18	21	-0.95	31.03	0.347	0.848	-0.31
12	150.53	104.66	21	217.93	182.27	23	-1.52	35.64	0.137	0.848	-0.45
18	119.66	107.29	20	177.64	123.34	20	-1.59	37.29	0.121	0.848	-0.50
24	87.35	49.97	17	159.02	174.98	22	-1.83	25.30	0.079	0.636	-0.53

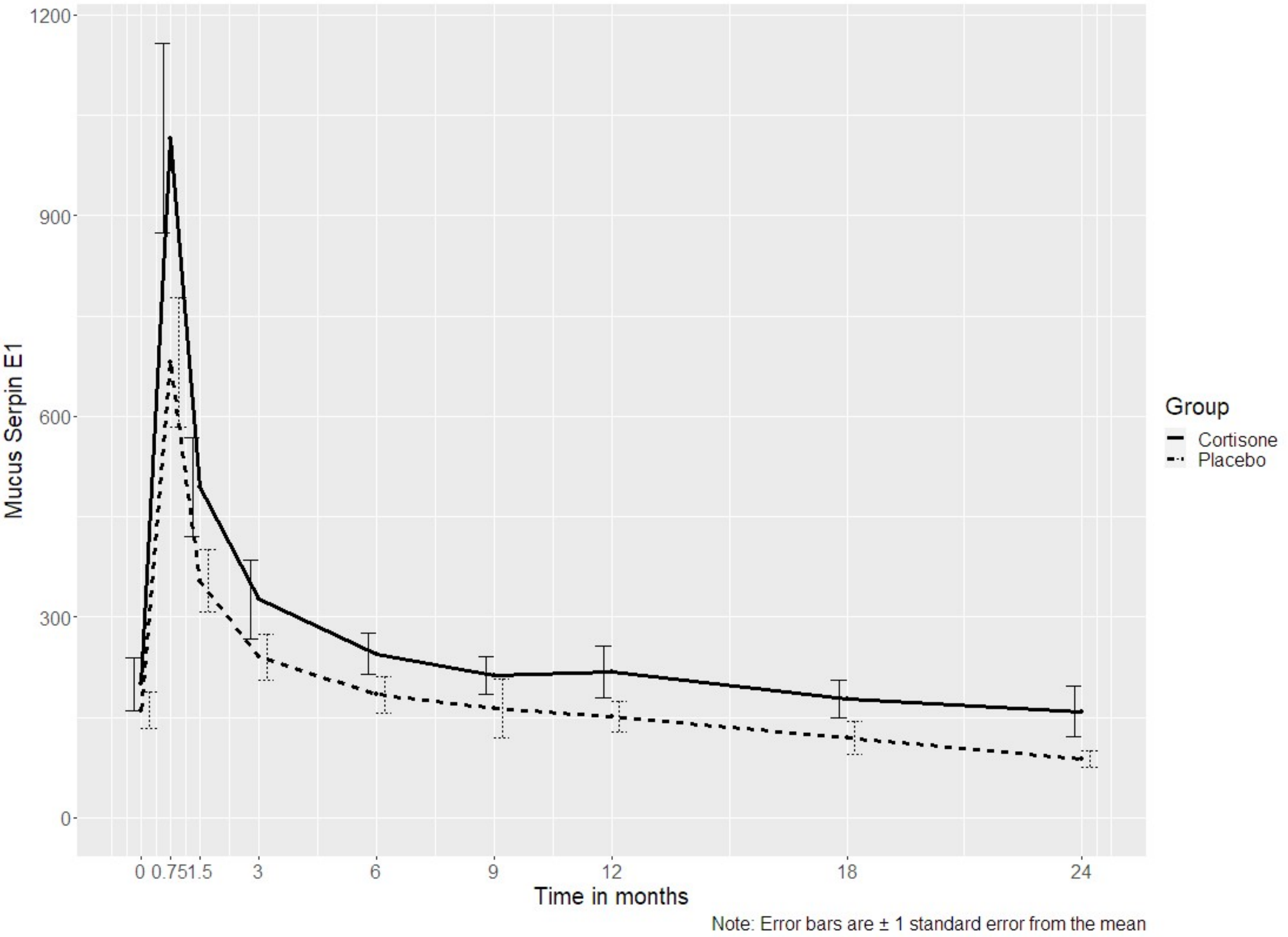

Mucus CST2

Linear mixed model results

	df1	df2	F	p	R^2
Time	8	404.368	5.936	0.000	0.058
Group	1	56.942	1.447	0.234	0.011
Interaction	8	404.368	1.188	0.304	0.011

Descriptive statistics for both groups across time

	M_Placebo	SD_Placebo	N_Placebo	M_Cortisone	SD_Cortisone	N_Cortisone
0	338.32	456.97	27	310.58	353.67	30
0.75	199.64	274.36	24	101.30	124.41	29
1.5	194.71	373.23	25	90.97	127.83	25
3	80.54	74.62	24	131.27	175.84	29
6	161.64	227.85	25	140.13	125.93	27
9	290.12	405.46	24	149.73	148.22	28
12	283.11	343.37	27	176.66	191.14	30
18	264.75	261.73	26	191.79	204.71	28
24	220.19	143.29	21	187.10	197.26	28

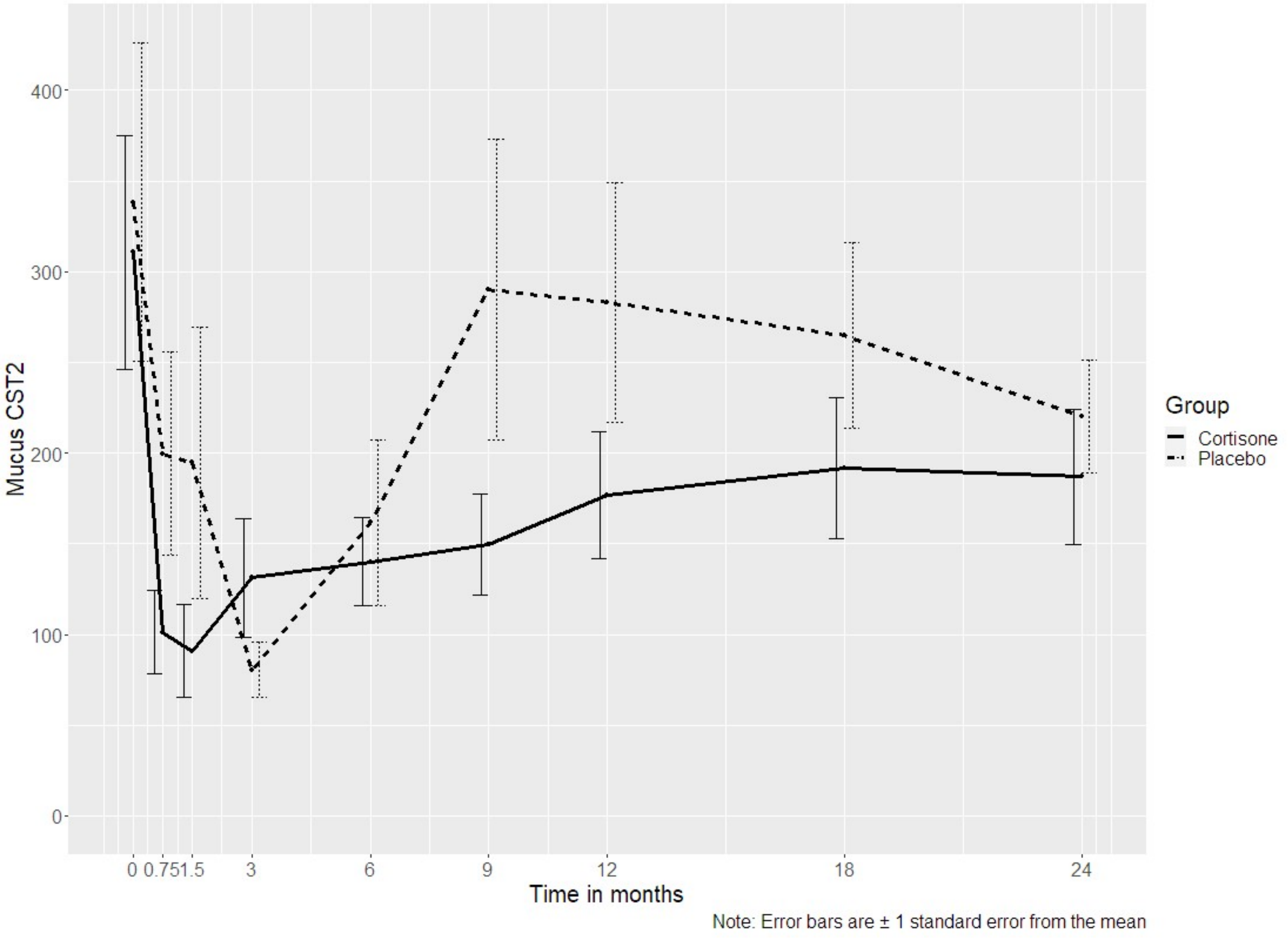

Mucus Serpin F2

Linear mixed model results

	df1	df2	F	p	R^2
Time	8	411.534	15.634	0.000	0.137

	df1	df2	F	p	R^2
Group	1	57.657	0.302	0.585	0.002
Interaction	8	411.534	1.784	0.078	0.014

Descriptive statistics for both groups across time

	M_Placebo	SD_Placebo	N_Placebo	M_Cortisone	SD_Cortisone	N_Cortisone
0	2608.57	1637.93	27	2919.59	1763.74	30
0.75	612.56	790.00	24	636.04	708.80	29
1.5	751.71	924.18	24	1160.80	1428.03	25
3	999.67	1477.61	24	1253.96	1374.45	29
6	1497.11	1879.37	27	1344.18	1362.53	27
9	1858.75	1273.12	25	1663.70	2073.74	28
12	1943.26	1552.62	27	1515.15	1670.05	30
18	2275.06	1496.34	26	1400.85	1361.81	28
24	2180.98	1615.77	26	1636.14	1878.48	28

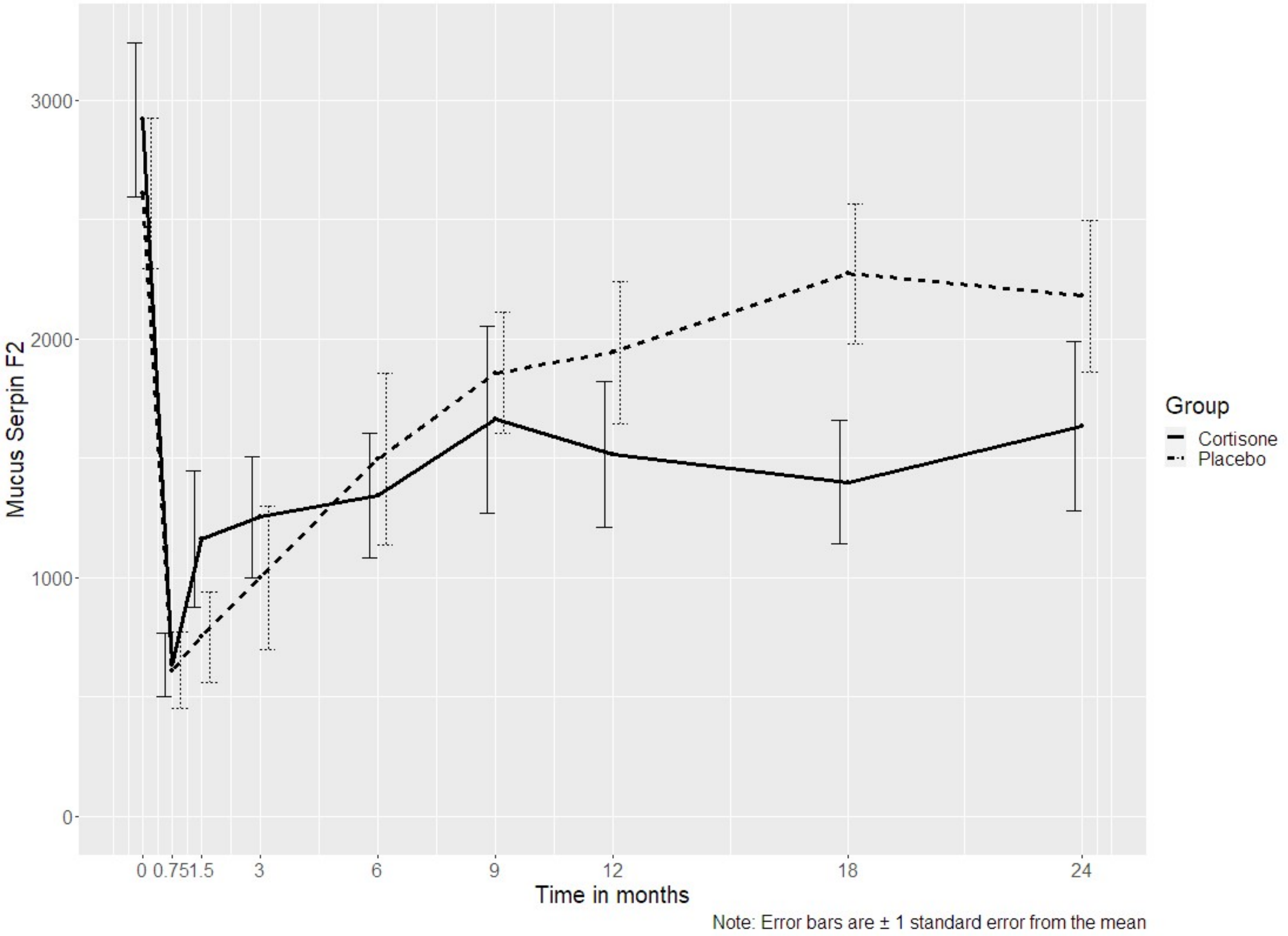

Mucus Periostin

Linear mixed model results

	df1	df2	F	p	R^2
Time	8	407.124	8.886	0.000	0.115
Group	1	58.058	0.026	0.873	0.000
Interaction	8	407.124	0.389	0.926	0.003

Descriptive statistics for both groups across time

	M_Placebo	SD_Placebo	N_Placebo	M_Cortisone	SD_Cortisone	N_Cortisone
0	160.06	181.96	26	184.00	333.58	30
0.75	41.84	48.29	23	36.31	57.73	29
1.5	46.27	36.53	23	52.97	56.68	25
3	52.33	60.75	24	73.05	64.31	29
6	60.33	86.79	26	56.12	52.00	27
9	70.32	52.99	24	64.32	73.11	28
12	61.69	46.77	26	46.49	49.00	30
18	56.85	41.87	25	46.84	53.93	28
24	72.89	83.67	24	42.54	49.65	28

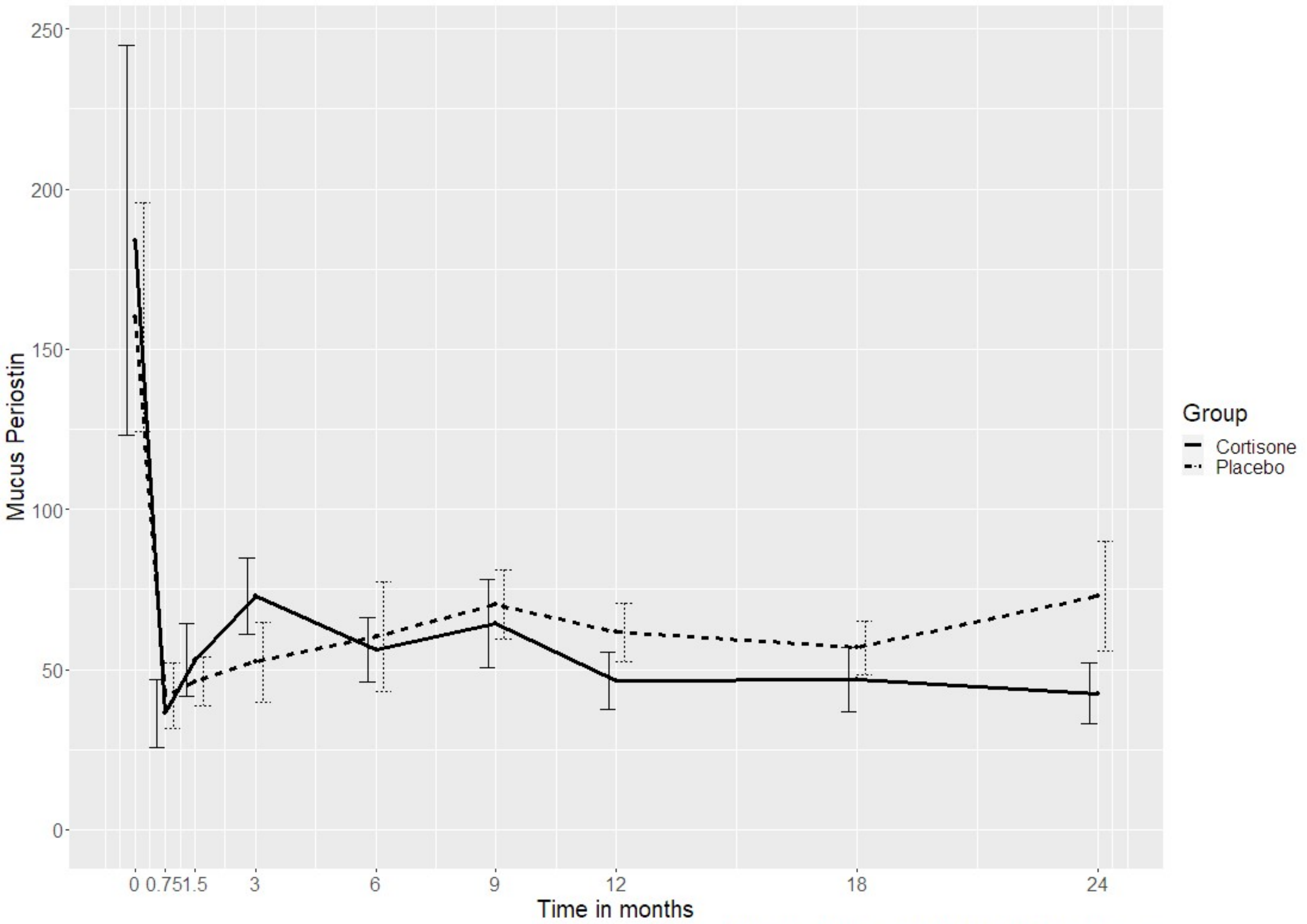

Note: Error bars are ± 1 standard error from the mean

Mucus PAPP-A

Linear mixed model results

	df1	df2	F	p	R²
Time	8	399.45	8.032	0.000	0.027
Group	1	56.08	0.015	0.904	0.000
Interaction	8	399.45	0.337	0.952	0.001

Descriptive statistics for both groups across time

	M_Placebo	SD_Placebo	N_Placebo	M_Cortisone	SD_Cortisone	N_Cortisone
0	109.08	77.79	26	110.97	94.42	29
0.75	73.99	88.37	23	65.14	85.14	29
1.5	62.21	65.17	24	83.80	132.45	25
3	80.05	99.02	24	79.25	122.94	29
6	69.74	74.07	26	71.85	90.23	27
9	74.95	99.02	24	75.02	112.85	28
12	65.35	88.73	26	72.60	119.57	30

	M_Placebo	SD_Placebo	N_Placebo	M_Cortisone	SD_Cortisone	N_Cortisone
18	59.59	55.09	25	58.72	90.10	28
24	61.60	57.63	22	48.76	79.93	28

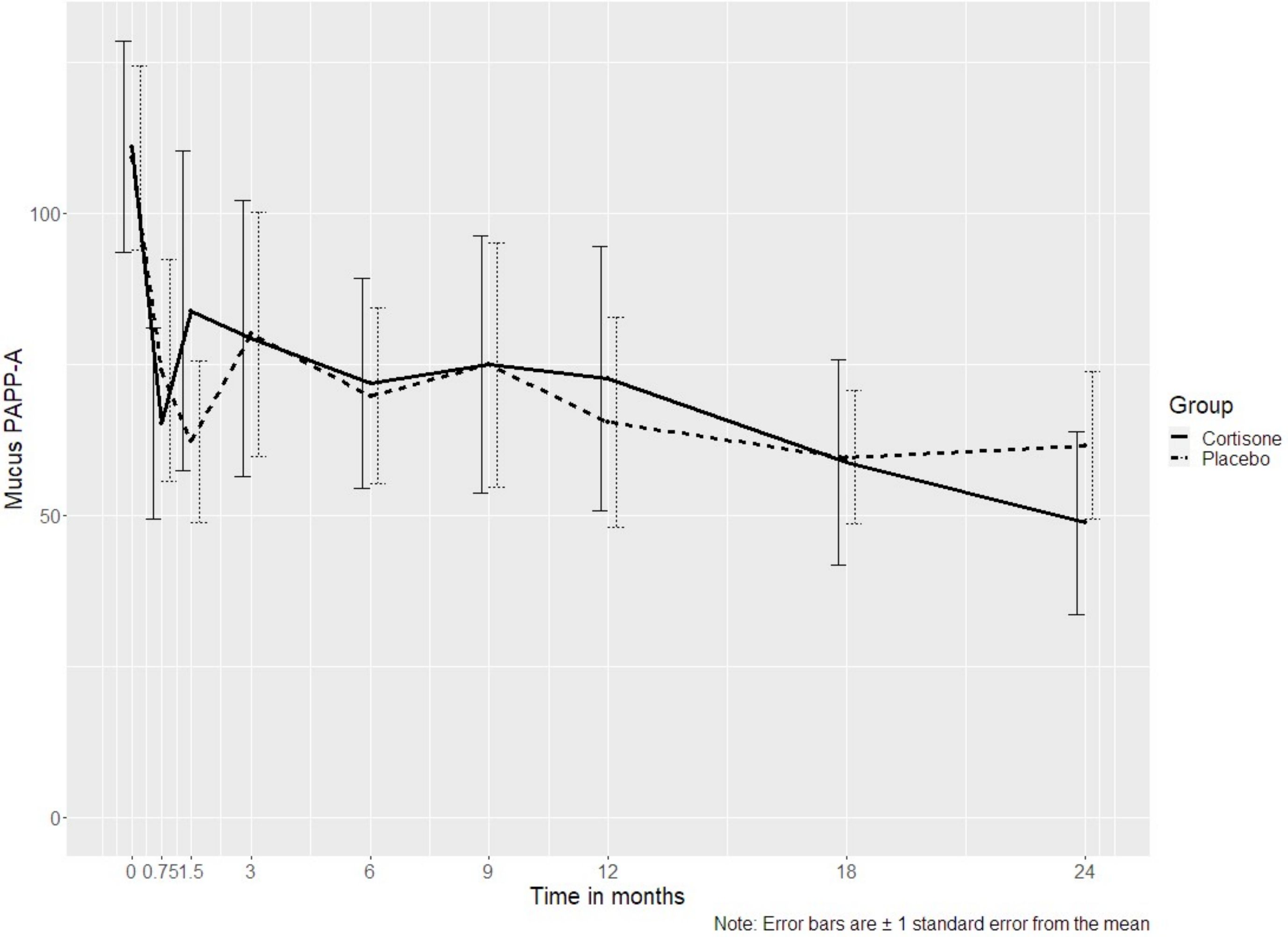

Mucus CST1

Linear mixed model results

	df1	df2	F	p	R²
Time	8	356.260	5.225	0.000	0.056
Group	1	59.887	0.167	0.685	0.002
Interaction	8	356.260	0.486	0.866	0.004

Descriptive statistics for both groups across time

	M_Placebo	SD_Placebo	N_Placebo	M_Cortisone	SD_Cortisone	N_Cortisone
0	11.32	12.43	26	15.87	20.91	31
0.75	6.54	9.33	21	3.07	3.64	27
1.5	4.76	6.05	21	7.02	11.13	27
3	8.62	12.25	24	11.35	17.44	26

	M_Placebo	SD_Placebo	N_Placebo	M_Cortisone	SD_Cortisone	N_Cortisone
6	11.12	15.68	24	14.14	20.94	27
9	15.09	20.57	23	13.61	12.45	28
12	17.64	23.99	21	18.12	21.31	26
18	16.96	21.88	20	19.44	19.72	22
24	18.76	30.58	14	13.38	14.33	16

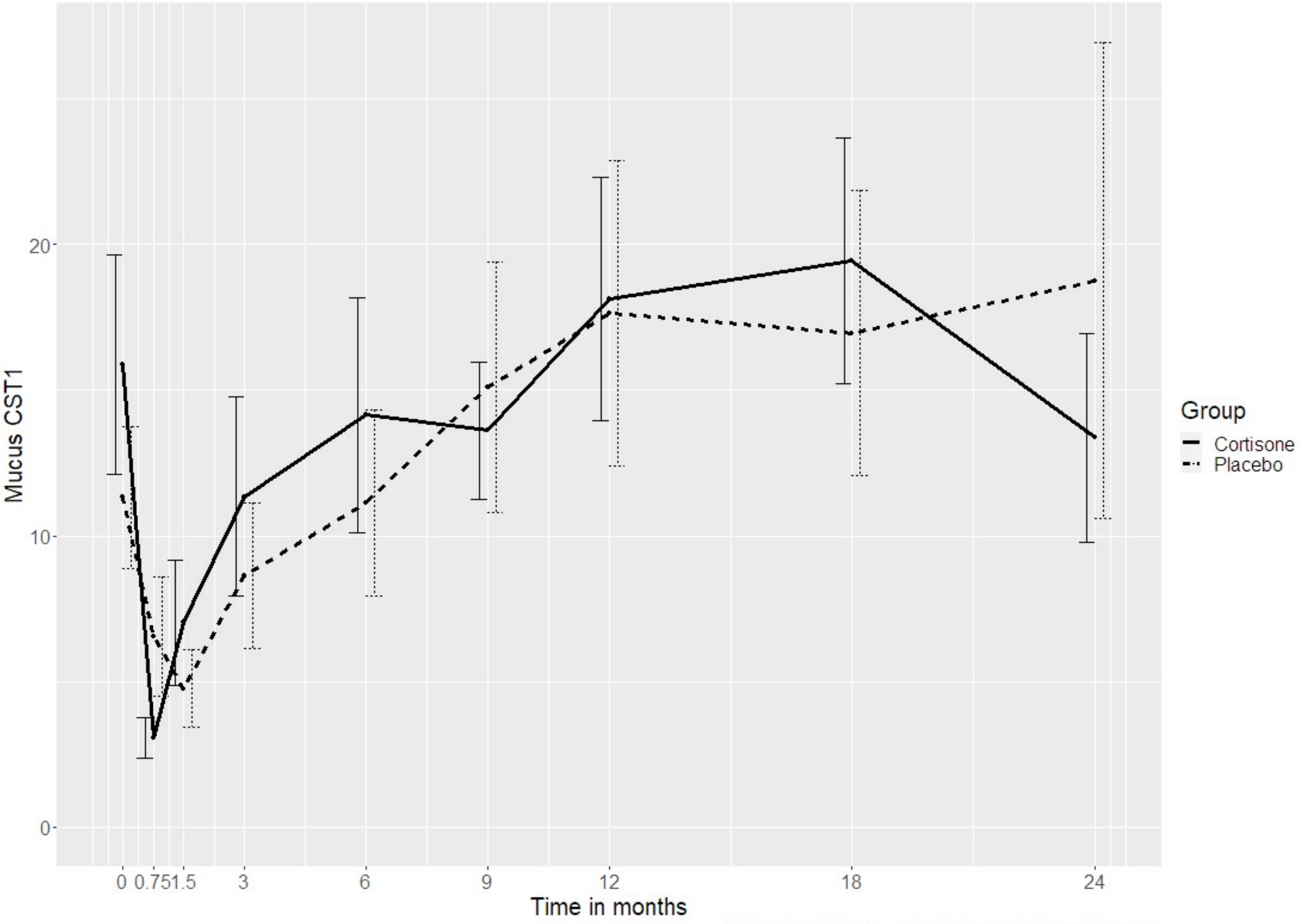

Mucus IgE

Linear mixed model results

	df1	df2	F	p	R^2
Time	8	403.465	2.539	0.010	0.023
Group	1	56.821	1.702	0.197	0.015
Interaction	8	403.465	0.832	0.575	0.007

Descriptive statistics for both groups across time

	M_Placebo	SD_Placebo	N_Placebo	M_Cortisone	SD_Cortisone	N_Cortisone
0	10.52	16.36	27	13.46	19.29	30

	M_Placebo	SD_Placebo	N_Placebo	M_Cortisone	SD_Cortisone	N_Cortisone
0.75	8.24	9.53	28	6.94	6.75	31
1.5	5.32	4.30	27	7.06	9.08	30
3	5.57	6.20	28	8.22	13.31	31
6	6.96	11.84	28	10.88	19.26	31
9	6.13	9.30	26	10.64	17.18	31
12	4.00	3.46	26	11.52	18.51	29
18	3.72	3.27	24	6.18	5.93	23
24	2.92	1.37	15	5.45	8.37	13

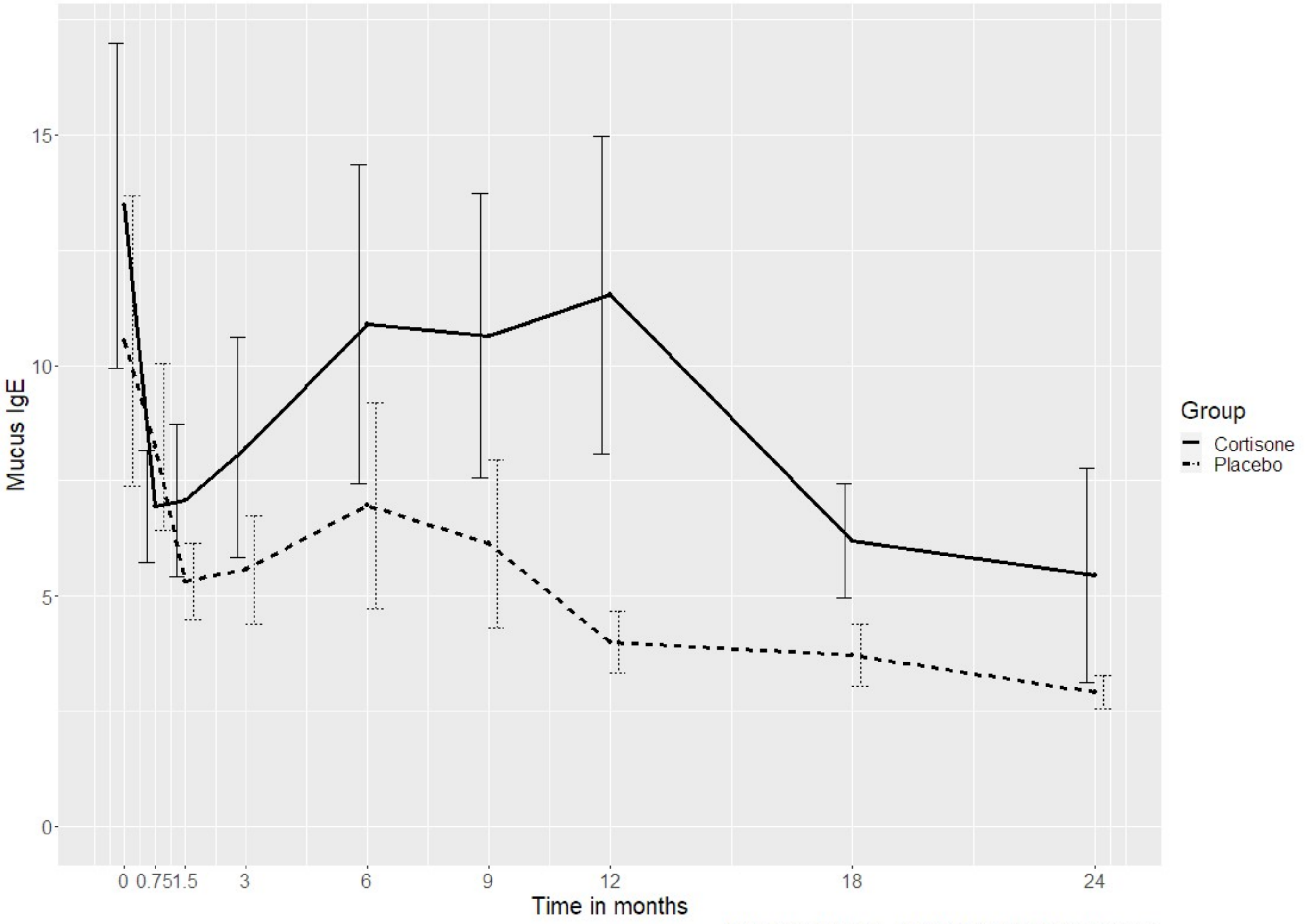

Note: Error bars are ± 1 standard error from the mean

Mucus MMP9

Linear mixed model results

	df1	df2	F	p	R^2
Time	8	409.661	14.039	0.000	0.153
Group	1	58.804	3.995	0.050	0.017
Interaction	8	409.661	0.620	0.761	0.005

Descriptive statistics for both groups across time

	M_Placebo	SD_Placebo	N_Placebo	M_Cortisone	SD_Cortisone	N_Cortisone
0	84.39	126.98	27	138.33	107.25	30
0.75	246.86	135.78	28	313.68	157.96	31
1.5	237.73	143.20	28	251.16	144.22	30
3	199.59	157.89	27	216.52	152.21	31
6	154.34	113.74	28	205.37	133.91	31
9	140.90	97.09	26	167.64	114.09	31
12	144.34	103.66	26	151.35	113.95	29
18	106.20	109.56	24	145.33	118.65	23
24	74.67	39.71	15	159.22	131.31	16

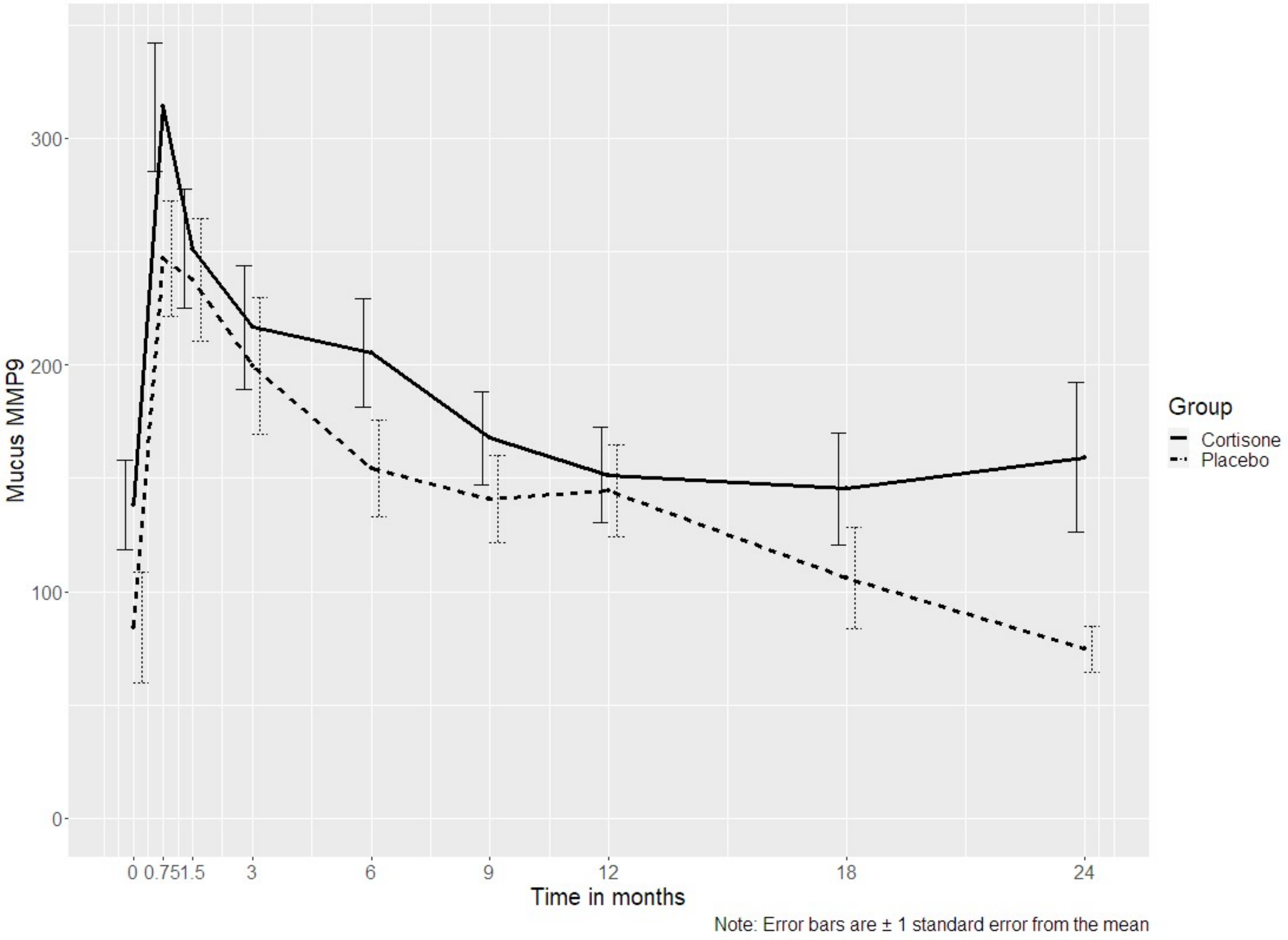

SF36 - General Health Perceptions

Linear mixed model results

	df1	df2	F	p	R²
Time	8	425.729	7.724	0.000	0.040

	df1	df2	F	p	R^2
Group	1	58.955	2.920	0.093	0.032
Interaction	8	425.729	0.340	0.950	0.001

Descriptive statistics for both groups across time

	M_Placebo	SD_Placebo	N_Placebo	M_Cortisone	SD_Cortisone	N_Cortisone
0	63.89	16.43	28	56.73	17.78	33
0.75	70.44	18.27	25	66.52	15.45	31
1.5	76.17	15.99	23	69.93	16.18	27
3	74.81	18.83	26	67.32	19.28	31
6	76.63	18.48	27	69.04	17.86	28
9	75.72	18.80	25	65.83	18.34	29
12	72.96	20.36	27	69.23	18.37	30
18	75.12	21.18	26	67.36	19.61	28
24	72.78	20.14	27	68.23	21.50	31

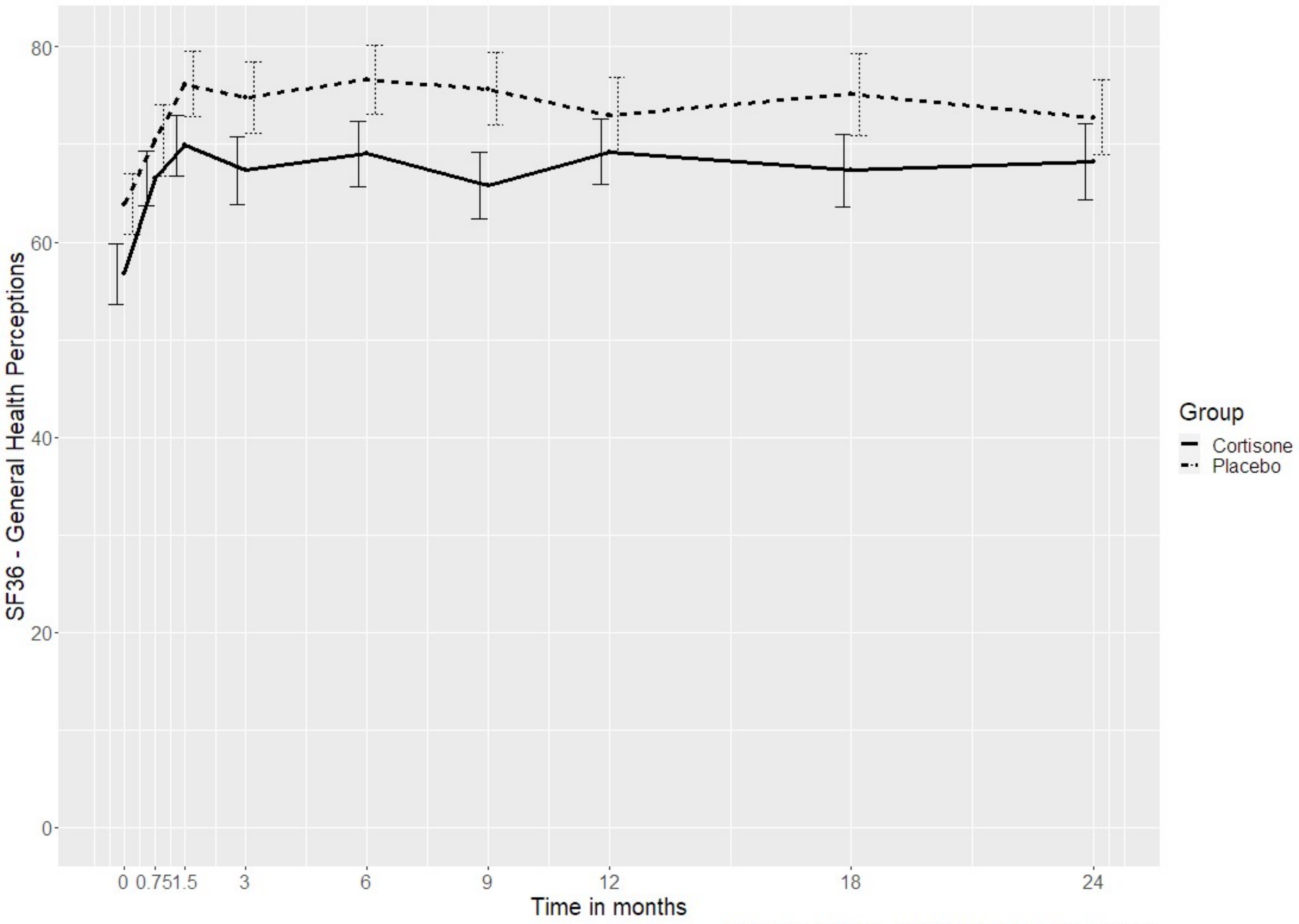

Note: Error bars are ± 1 standard error from the mean

SF36 - Mental health index

Linear mixed model results

	df1	df2	F	p	R^2
Time	8	425.906	5.692	0.000	0.041
Group	1	58.686	1.133	0.291	0.012
Interaction	8	425.906	1.017	0.422	0.007

Descriptive statistics for both groups across time

	M_Placebo	SD_Placebo	N_Placebo	M_Cortisone	SD_Cortisone	N_Cortisone
0	77.86	15.22	28	71.27	16.48	33
0.75	74.72	16.32	25	74.58	13.80	31
1.5	81.91	12.53	23	80.59	11.88	27
3	83.54	11.72	26	81.29	10.45	31
6	81.04	11.45	27	80.29	14.15	28
9	81.76	13.96	25	75.86	16.99	29
12	80.74	15.41	27	82.00	11.00	30
18	81.69	12.71	26	78.43	17.26	28
24	83.56	12.22	27	80.52	14.56	31

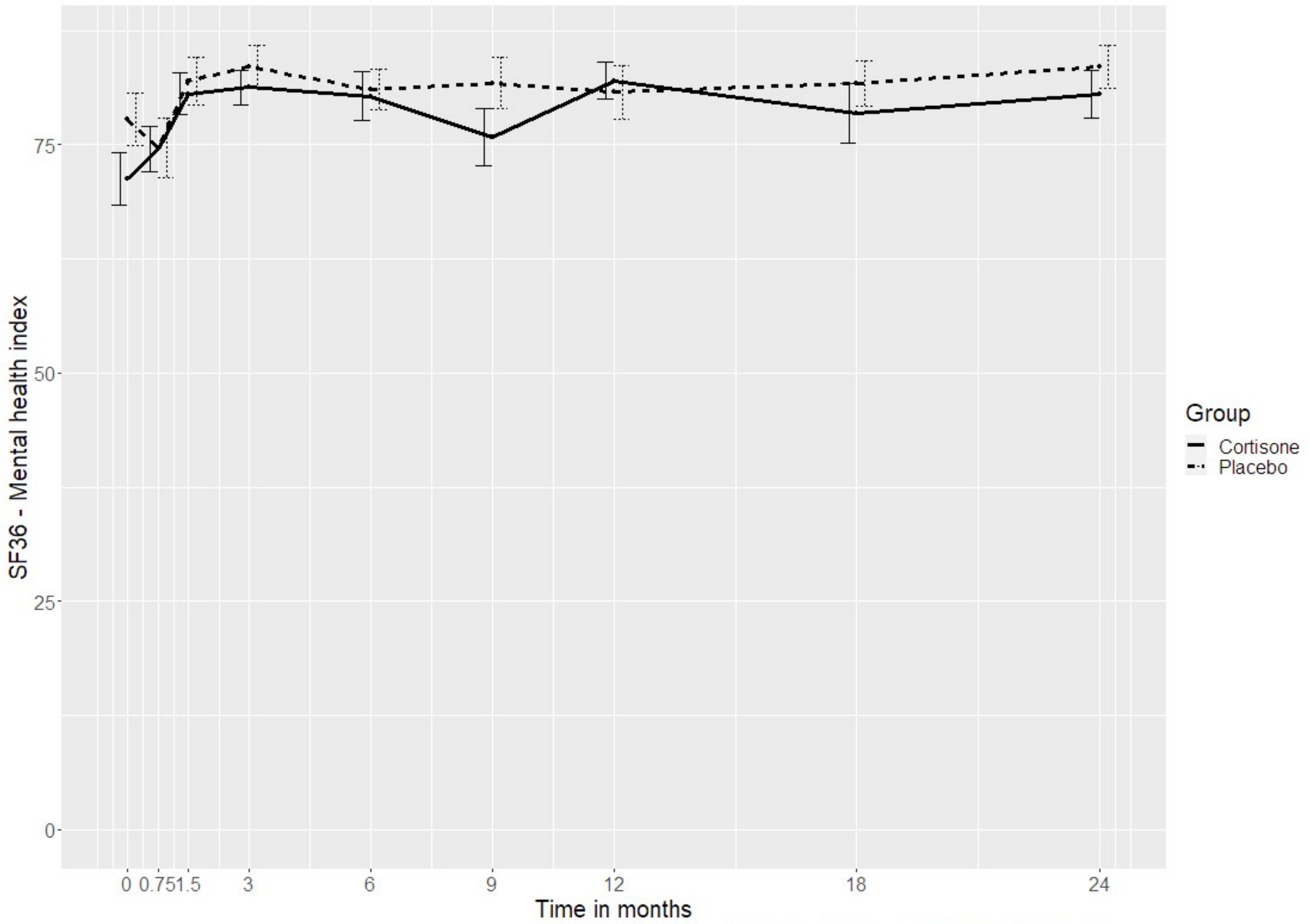

Note: Error bars are ± 1 standard error from the mean

SF36 - Pain Index

Linear mixed model results

	df1	df2	F	p	R^2
Time	8	427.439	24.171	0.000	0.190
Group	1	59.581	0.044	0.834	0.001
Interaction	8	427.439	2.196	0.027	0.015

Descriptive statistics for both groups across time

	M_Placebo	SD_Placebo	N_Placebo	M_Cortisone	SD_Cortisone	N_Cortisone	t	df	p	Holm_p	Cohen d
0	76.93	16.98	28	65.94	22.44	33	2.17	58.30	0.034	0.304	0.55
0.75	58.20	22.02	25	56.97	23.57	31	0.20	52.78	0.841	1.000	0.05
1.5	75.00	25.25	23	80.33	13.74	27	-0.91	32.74	0.372	1.000	-0.27
3	81.88	16.41	26	80.68	14.05	31	0.30	49.59	0.769	1.000	0.08
6	81.22	16.49	27	85.25	8.55	28	-1.13	38.72	0.265	1.000	-0.31
9	84.32	14.81	25	82.00	12.93	29	0.61	48.11	0.546	1.000	0.17

	M_Placebo	SD_Placebo	N_Placebo	M_Cortisone	SD_Cortisone	N_Cortisone	t	df	p	Holm_p	Cohen d
12	80.85	16.65	27	84.23	10.79	30	-0.90	43.75	0.374	1.000	-0.24
18	85.00	13.48	26	80.82	16.28	28	1.03	51.36	0.308	1.000	0.28
24	81.56	15.60	27	82.61	14.83	31	-0.26	54.03	0.793	1.000	-0.07

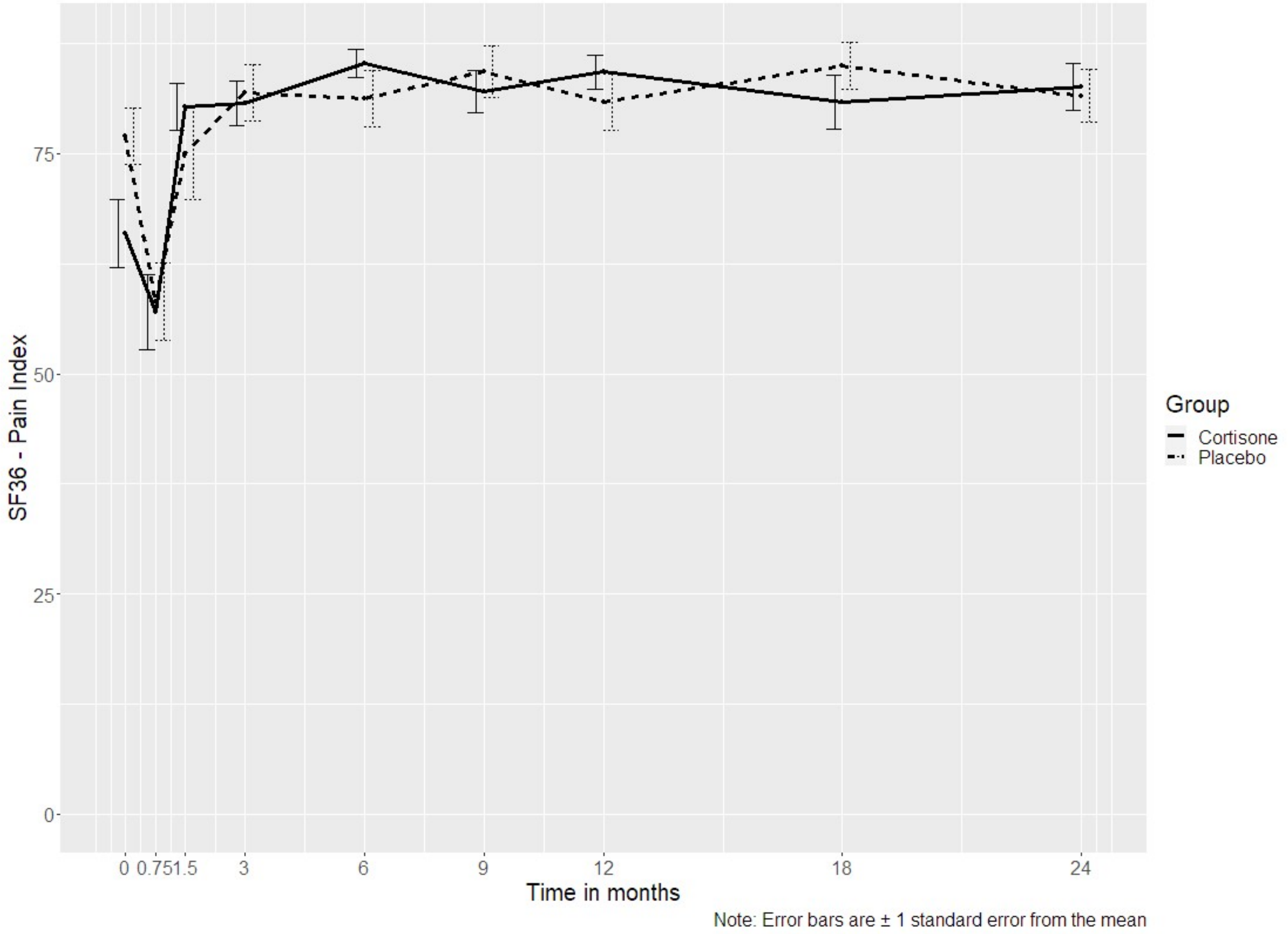

SF36 - Physical Functioning

Linear mixed model results

	df1	df2	F	p	R^2
Time	8	424.850	5.406	0.000	0.031
Group	1	58.975	1.753	0.191	0.020
Interaction	8	424.850	0.620	0.762	0.003

Descriptive statistics for both groups across time

	M_Placebo	SD_Placebo	N_Placebo	M_Cortisone	SD_Cortisone	N_Cortisone
0	88.75	12.81	28	83.94	16.67	33
0.75	90.00	10.70	25	87.26	11.09	31

	M_Placebo	SD_Placebo	N_Placebo	M_Cortisone	SD_Cortisone	N_Cortisone
1.5	93.26	7.78	23	89.63	12.93	27
3	92.50	10.32	26	90.16	17.10	31
6	94.26	9.06	27	89.29	21.03	28
9	95.60	7.54	25	91.61	14.28	28
12	93.89	11.21	27	91.00	12.21	30
18	95.19	10.53	26	87.14	19.55	28
24	94.63	8.98	27	91.94	12.02	31

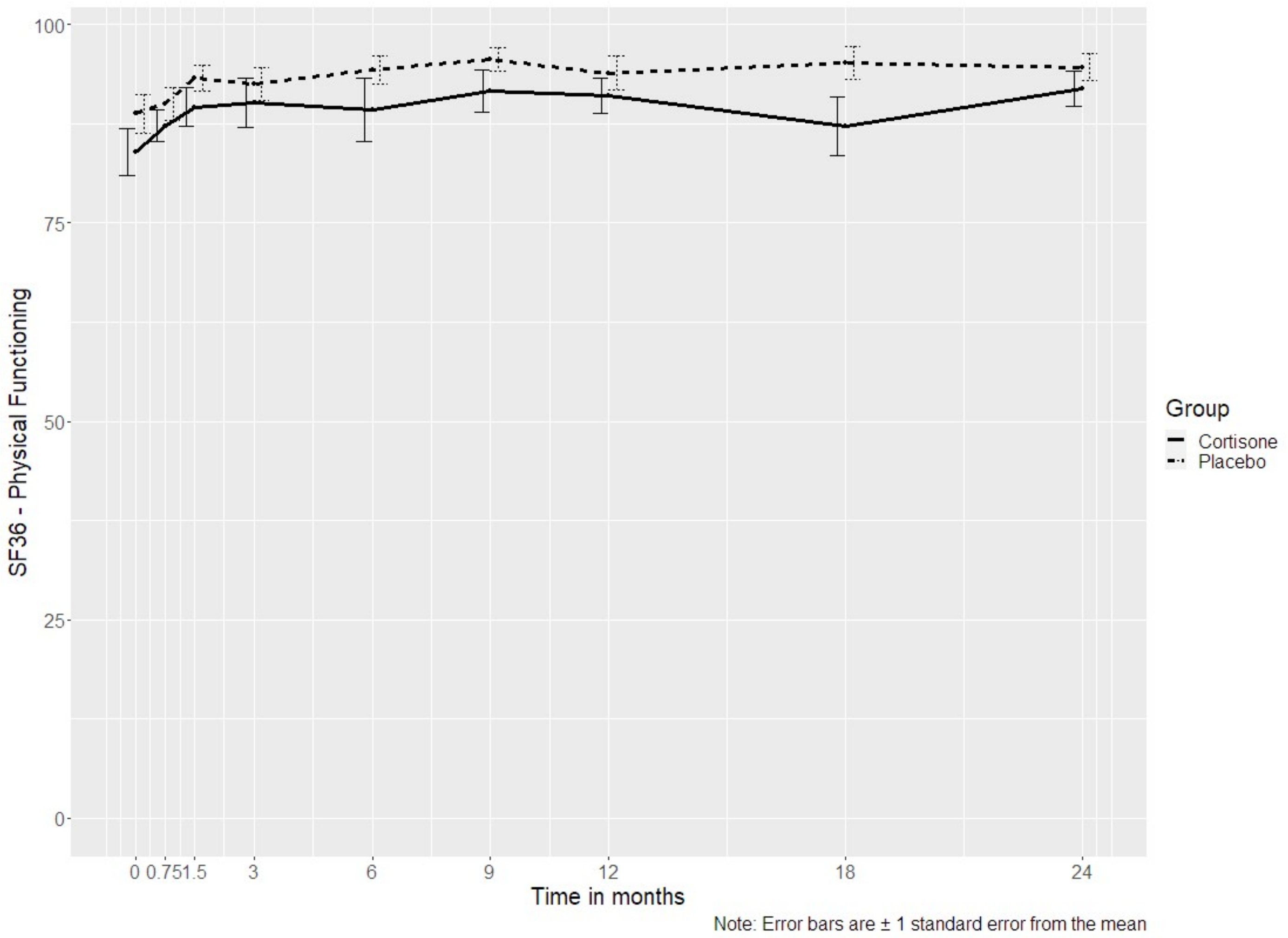

SF36 - Role-Physical

Linear mixed model results

	df1	df2	F	p	R^2
Time	8	427.102	9.363	0.000	0.101
Group	1	59.033	0.286	0.595	0.002
Interaction	8	427.102	1.217	0.287	0.012

Descriptive statistics for both groups across time

	M_Placebo	SD_Placebo	N_Placebo	M_Cortisone	SD_Cortisone	N_Cortisone
0	84.82	25.77	28	69.70	36.85	33
0.75	61.00	41.51	25	63.71	42.74	31
1.5	83.70	32.52	23	80.56	32.77	27
3	91.35	27.33	26	87.10	28.04	31
6	88.89	28.02	27	97.32	7.87	28
9	97.00	10.99	25	92.86	23.43	28
12	88.89	29.69	27	95.00	16.61	30
18	89.42	28.44	26	80.36	35.59	28
24	92.59	19.38	27	89.52	25.64	31

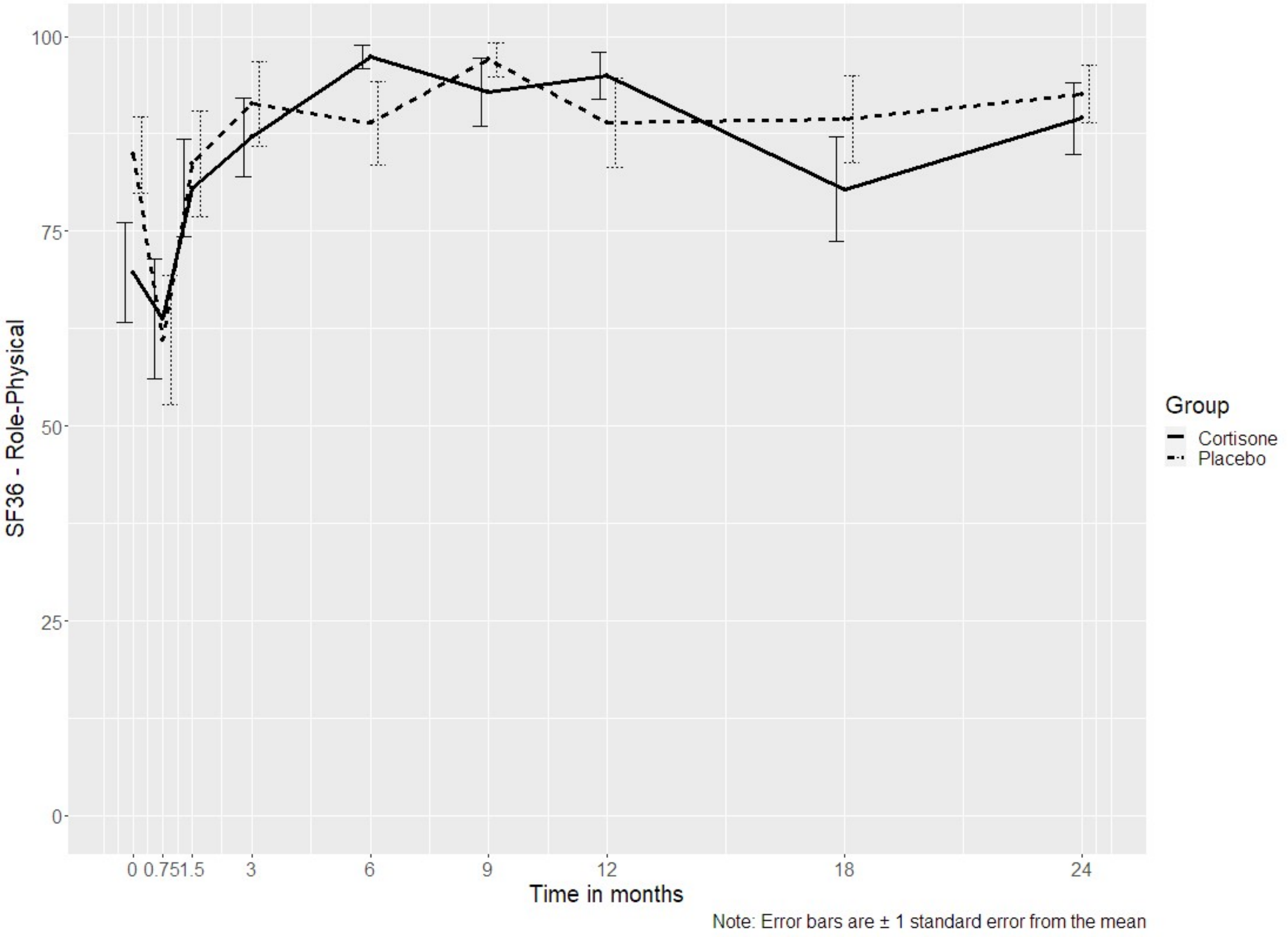

SF36 - Role Emotional

Linear mixed model results

	df1	df2	F	p	R²
Time	8	428.564	9.743	0.000	0.115

	df1	df2	F	p	R²
Group	1	58.675	0.599	0.442	0.004
Interaction	8	428.564	0.842	0.566	0.008

Descriptive statistics for both groups across time

	M_Placebo	SD_Placebo	N_Placebo	M_Cortisone	SD_Cortisone	N_Cortisone
0	85.71	29.30	28	73.74	39.75	33
0.75	66.67	41.94	25	62.37	45.33	31
1.5	85.51	34.56	23	82.72	33.80	27
3	97.44	9.06	26	93.55	21.81	31
6	92.59	23.27	27	96.43	10.50	28
9	97.33	13.33	25	89.66	25.36	29
12	90.12	25.84	27	96.67	10.17	30
18	97.44	13.08	26	89.29	27.30	28
24	92.59	23.27	27	92.47	25.40	31

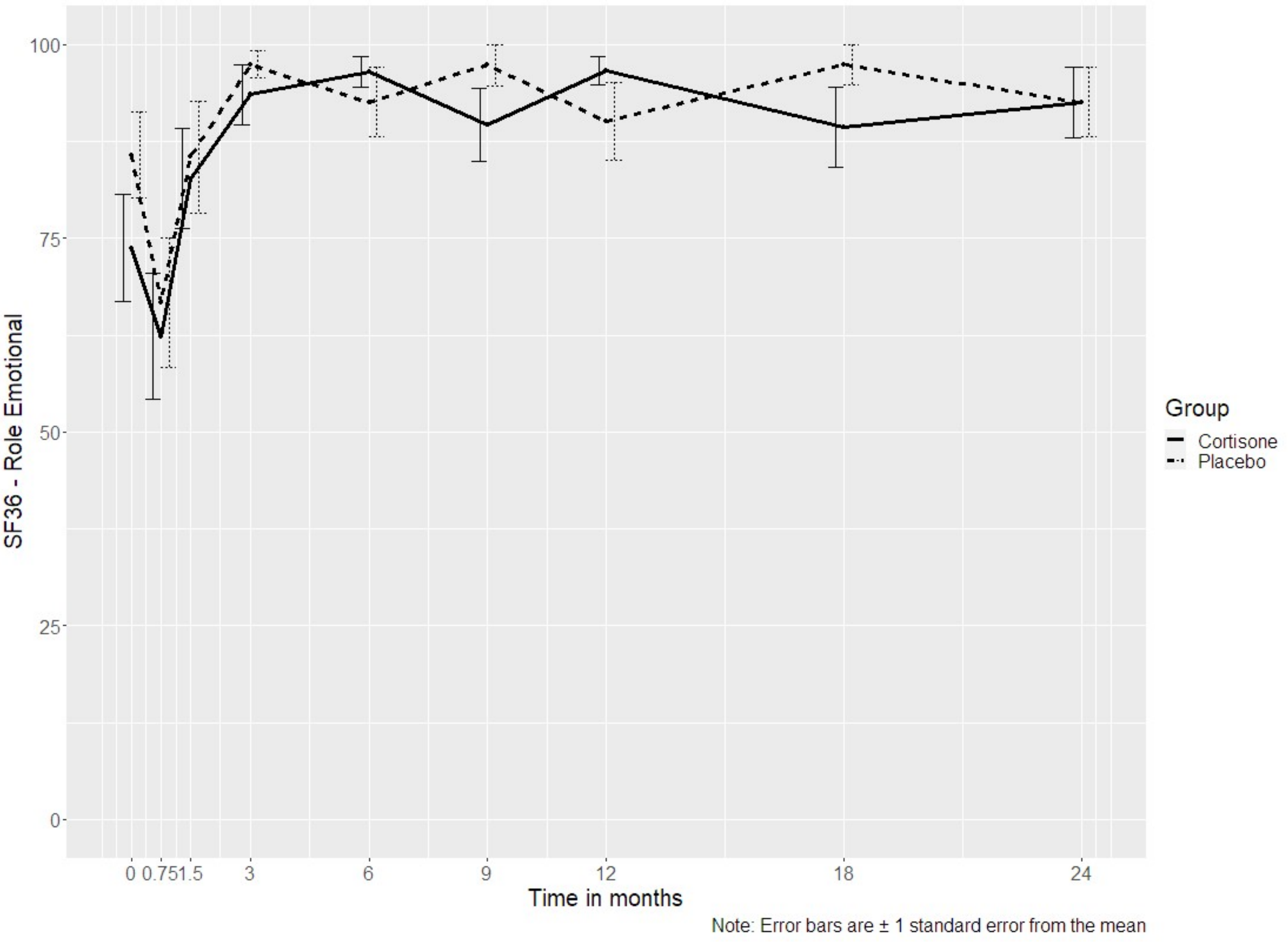

SF36 - Social Functioning

Linear mixed model results

	df1	df2	F	p	R^2
Time	8	427.230	13.321	0.000	0.132
Group	1	58.521	0.305	0.583	0.002
Interaction	8	427.230	1.490	0.159	0.013

Descriptive statistics for both groups across time

	M_Placebo	SD_Placebo	N_Placebo	M_Cortisone	SD_Cortisone	N_Cortisone
0	84.82	19.05	28	77.65	19.70	33
0.75	76.50	20.51	25	77.02	22.15	31
1.5	94.02	12.43	23	91.67	13.43	27
3	91.83	15.79	26	93.15	13.24	31
6	93.52	14.86	27	95.98	9.04	28
9	95.50	12.44	25	92.67	12.28	29
12	87.50	18.34	27	94.17	13.43	30
18	95.67	9.32	26	89.73	15.61	28
24	94.44	10.01	27	92.34	15.03	31

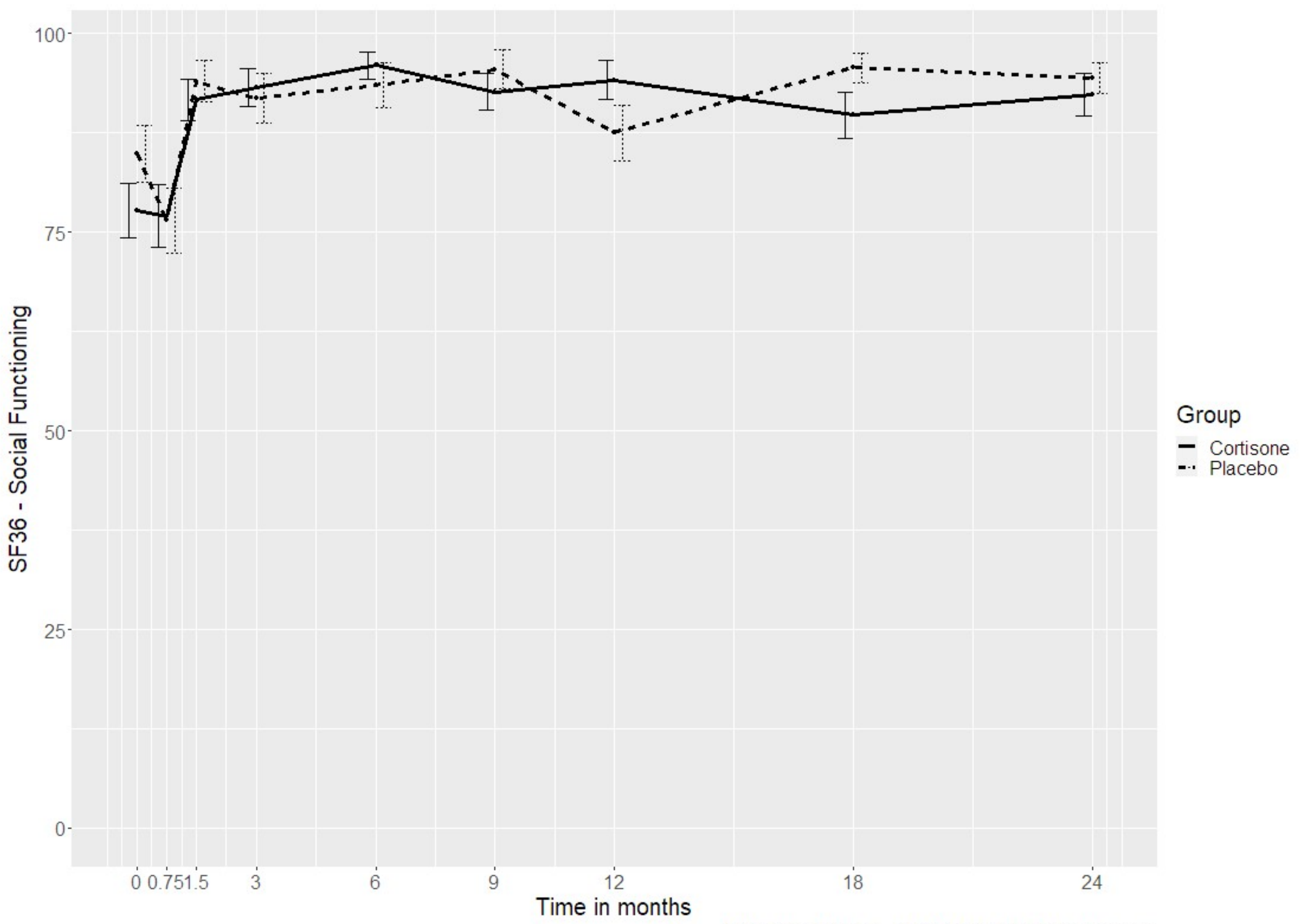

Note: Error bars are ± 1 standard error from the mean

SF36 - Vitality

Linear mixed model results

	df1	df2	F	p	R^2
Time	8	425.787	13.546	0.000	0.071
Group	1	59.003	1.574	0.215	0.019
Interaction	8	425.787	1.006	0.431	0.005

Descriptive statistics for both groups across time

	M_Placebo	SD_Placebo	N_Placebo	M_Cortisone	SD_Cortisone	N_Cortisone
0	66.96	18.48	28	56.06	22.60	33
0.75	61.00	20.82	25	56.61	21.50	31
1.5	72.39	19.42	23	71.48	18.80	27
3	74.42	16.57	26	71.94	16.26	31
6	75.56	16.54	27	73.21	13.96	28
9	73.00	18.26	25	66.03	21.02	29
12	71.85	14.29	27	70.67	20.29	30

	M_Placebo	SD_Placebo	N_Placebo	M_Cortisone	SD_Cortisone	N_Cortisone
18	75.38	18.60	26	68.75	20.84	28
24	73.70	17.46	27	70.65	19.91	31

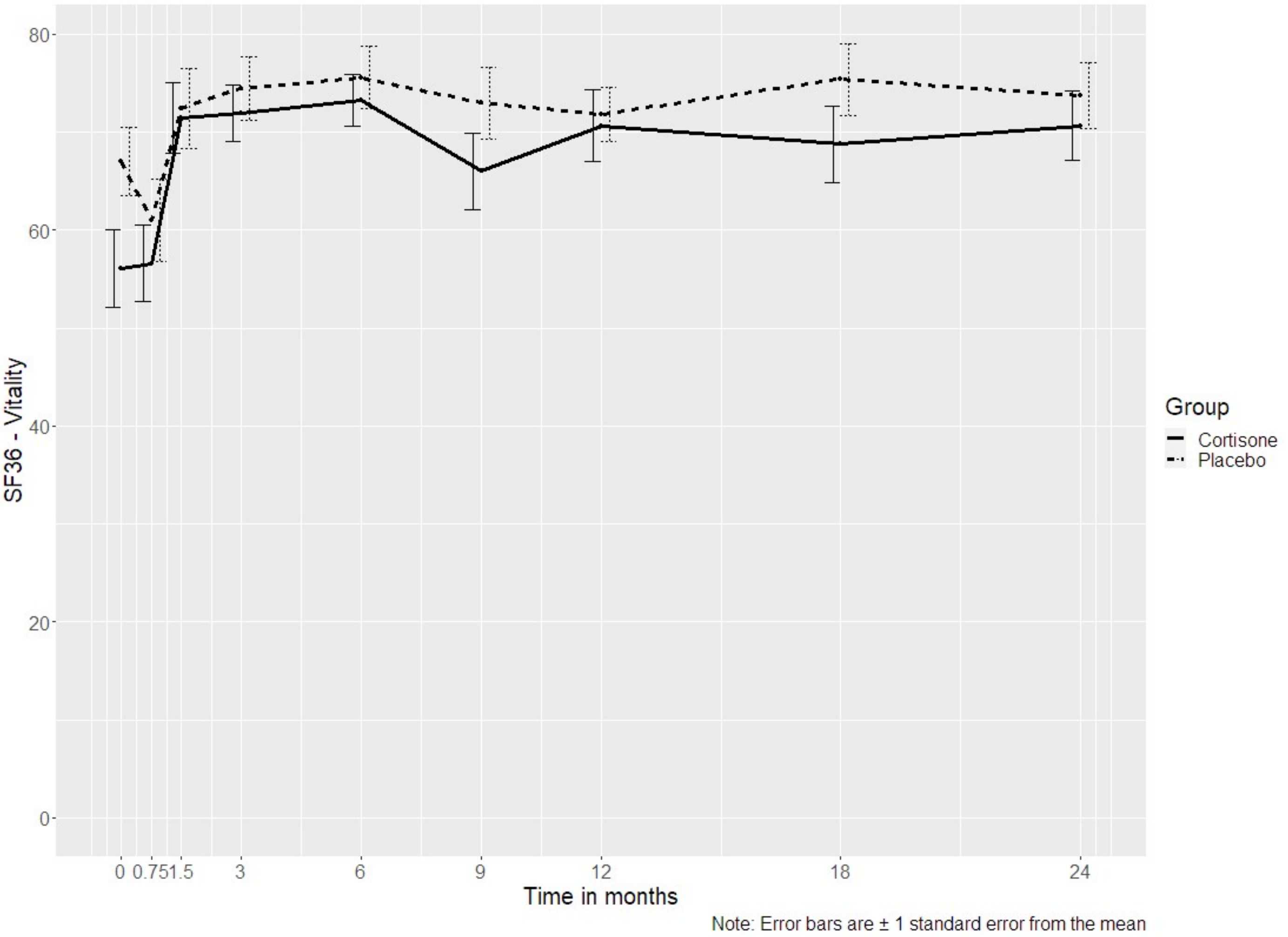

Supplement: Supplemental files — Line plots of all primary and secondary outcome measures for each of the 9 time points. Appendix: Protein Biomarker Analysis Using Enzyme Linked Immunosorbent Assay (ELISA). For PAPP-A, the nasal mucus was measured undiluted. The Human Pappalysin-1 DuoSet No. DY2487-05, R&D SYSTEMS (Bio-Techne GmbH, Wiesbaden, Germany) was used. For Periostin, the nasal mucus was diluted 1:800 with Reagent Diluent (included in the Kit). The Human Periostin DuoSet No. DY3548B, R&D SYSTEMS (Bio-Techne GmbH, Wiesbaden, Germany) was used. For SerpinF2, the nasal mucus was diluted 1:2 with Reagent Diluent. The Human Serpin F2 DuoSet No. DY1470-05, R&D SYSTEMS (Bio-Techne GmbH, Wiesbaden, Germany) was used. For SerpinE1, the nasal mucus was diluted 1:3 with 0.1% BSA in PBS. The Human Serpin E1 ELISA Pair Set, No. SEK10296, Sino Biological Inc. (Beijing, China) was used. For CST2, the nasal mucus was diluted 1:5 with 0.1% BSA in PBS. The Human Cystatin SA/CST2 ELISA Pair Set, No. SEK11567, Sino Biological Inc. (Beijing, China) was used. For CST1, the nasal mucus was diluted 1:200 with Reagent Diluent. The Human CST1 ELISA No. SEK11568, Sino Biological Inc. (Beijing, China) was used. For MMP9, the nasal mucus was diluted 1:800 with Reagent Diluent. The Human MMP9 DuoSet No. DY911, R&D SYSTEMS (Bio-Techne GmbH, Wiesbaden, Germany) was used. For IgE, the nasal mucus was diluted 1:2 with Reagent Diluent. The Human IgE ELISA No. 88-50610, Thermo Fisher (Thermo Fisher Scientic Inc, Waltham, Massachusetts, USA) was used. [file DataSheet_1.pdf]
