# Supplementary material for: Effect of postoperative systemic prednisolone on short-term and long-term outcomes in chronic rhinosinusitis with nasal polyps: A multi-centered randomized clinical trial
Source: Front Immunol. 2023 Mar 8;14:1075066. doi: 10.3389/fimmu.2023.1075066 (PMC10032209; doi:10.3389/fimmu.2023.1075066)

Supplementary File 2

Subgroup analysis

Sense of smell

Linear mixed model results

	df1	df2	F	p	R²
Time	8	307.096	11.924	0.000	0.103
Group	1	42.081	2.539	0.119	0.029
Interaction	8	307.096	2.113	0.034	0.018

Descriptive statistics for both groups across time

	M_Placebo	SD_Placebo	N_Placebo	M_Cortisone	SD_Cortisone	N_Cortisone	t	df	p	Holm_p	Cohen d
0	1.25	0.64	20	1.08	0.76	25	0.82	42.87	0.419	1.000	0.24
0.75	0.44	0.51	18	0.83	0.65	23	-2.10	39.00	0.042	0.317	-0.64
1.5	0.36	0.50	14	0.62	0.67	21	-1.33	32.52	0.194	0.912	-0.43
3	0.21	0.54	19	0.65	0.65	23	-2.42	40.00	0.020	0.181	-0.74
6	0.28	0.67	18	0.57	0.68	21	-1.36	36.21	0.182	0.912	-0.44
9	0.39	0.61	18	0.48	0.67	23	-0.45	38.01	0.657	1.000	-0.14
12	0.21	0.63	19	0.65	0.71	23	-2.13	39.80	0.040	0.317	-0.65
18	0.32	0.67	19	0.67	0.58	21	-1.76	35.75	0.086	0.518	-0.56
24	0.53	0.77	19	0.58	0.65	24	-0.26	35.32	0.799	1.000	-0.08

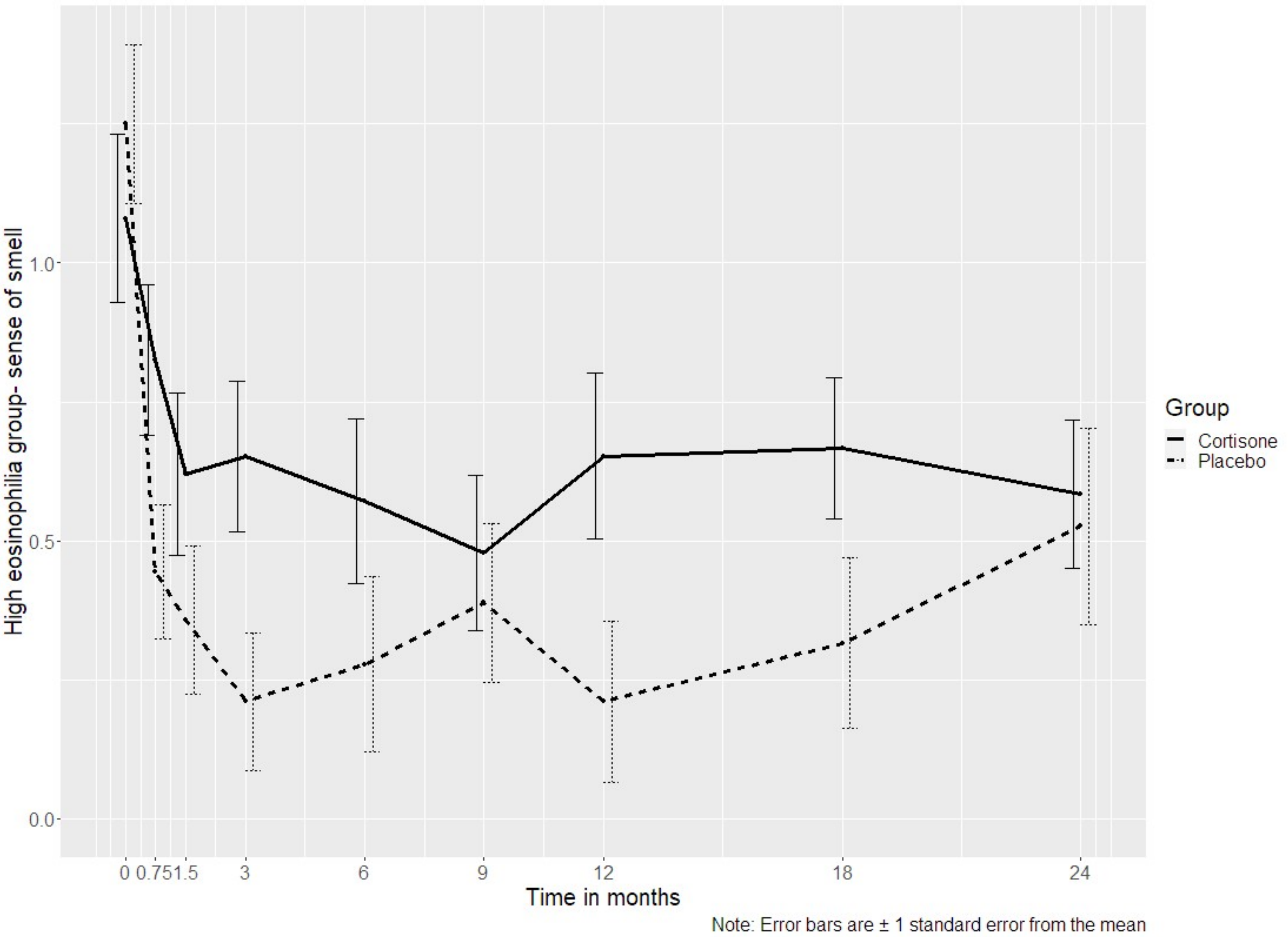

Odor identification

Linear mixed model results

	df1	df2	F	p	R^2
Time	8	271.274	3.339	0.001	0.031
Group	1	41.725	0.106	0.746	0.002
Interaction	8	271.274	1.470	0.168	0.012

Descriptive statistics for both groups across time

	M_Placebo	SD_Placebo	N_Placebo	M_Cortisone	SD_Cortisone	N_Cortisone
0	8.15	3.30	20	7.09	3.93	22
0.75	8.06	2.36	17	8.47	2.85	17
1.5	9.62	1.45	16	9.07	2.50	14
3	9.11	2.40	18	8.84	2.63	19
6	9.00	2.30	21	9.50	2.34	16
9	9.29	2.28	21	9.38	2.16	16
12	8.50	2.35	20	9.24	2.33	17

	M_Placebo	SD_Placebo	N_Placebo	M_Cortisone	SD_Cortisone	N_Cortisone
18	9.30	1.81	20	9.00	2.93	18
24	8.90	2.83	20	9.45	3.03	20

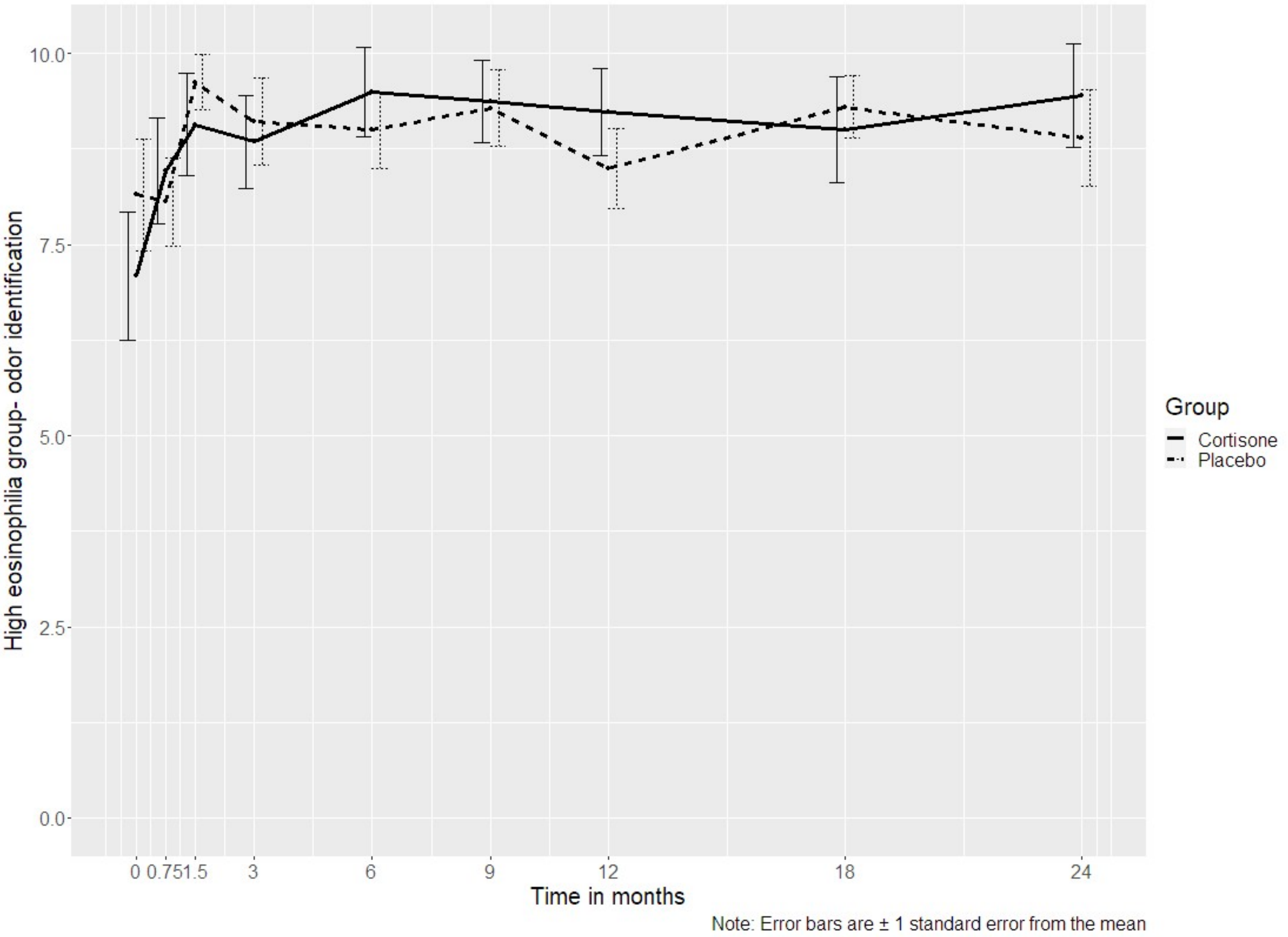

Supplement: Supplementary file 2 [file DataSheet_2.pdf]
